# Temporal trends (1972–2017) and spatial differences of persistent halogenated aromatic hydrocarbons in osprey eggs in Finland

**DOI:** 10.1371/journal.pone.0308227

**Published:** 2024-09-03

**Authors:** Matti Viluksela, Pertti Saurola, Juhani Koivusaari, Matts Finnlund, Anders Bignert, Riikka Airaksinen, Päivi Ruokojärvi, Matti Verta, Hannu Kiviranta, Jouni T. Tuomisto, Panu Rantakokko

**Affiliations:** 1 Finnish Institute for Health and Welfare, Environmental Health Unit, Kuopio, Finland; 2 School of Pharmacy (Toxicology) and Department of Environmental and Biological Sciences, University of Eastern Finland, Kuopio, Finland; 3 Finnish Museum of Natural History, Ringing Centre, University of Helsinki, Helsinki, Finland; 4 Finnish Environment Institute, Helsinki, Finland; 5 Yibin Research Base of the Key Laboratory of Yangtze River Water Environment of the Ministry of Education, Yibin University, Yibin, Sichuan Province, China; VIT University, INDIA

## Abstract

Time trends and regional differences of polychlorinated dibenzo-*p*-dioxins and -furans (PCDD/Fs), polychlorinated biphenyls (PCBs), polychlorinated naphthalenes (PCNs), DDTs, polybrominated biphenyls (PBBs) and polybrominated diphenylethers (BDEs) were studied in unhatched osprey eggs collected by bird ringers in 1972–2017 from four areas in Finland. Two study areas were from Baltic Sea, Northern Quark and Finnish Archipelago Sea, while the two others were inland lake areas, eutrophicated Lake Vanajanselkä affected by industrial emissions, and Pristine SW Lake Area. The highest concentrations of most compound groups were in Lake Vanajanselkä consistent with high emissions, the predominance of bream as a prey, and higher concentrations in bream compared to other prey fish. Concentrations of all chlorinated compounds decreased significantly in all study areas. Average annual decreases were ∑PCDD/F 2.3–4.9%, ∑PCB 2.2–4.2%, ∑PCN 2.6–7.0% and ∑DDT 7.1–9.5%, primarily in line with decreased levels in prey fish. From 1972 PBBs and BDEs increased significantly until 1990s declining rapidly thereafter. PCDD/F congener profile was dominated by 2,3,4,7,8-PeCDF, except in Lake Vanajanselkä by 1,2,3,6,7,8-HxCDD. PCB congener profile was dominated by PCB 153 in all study areas, followed by PCB 180 and PCB 138. Among dioxin-like compounds PCBs contributed 82%, PCDDs 14% and PCDFs 4% to toxic equivalent quantity (∑TEQ). PCB 126 contributed most to ∑TEQ, followed by 1,2,3,7,8-PeCDD. BDE 47 being the dominant BDE congener, followed by BDE 100. ∑DDT concentrations were relatively similar across all study areas, with DDE contributing about 90%. Productivity of chicks per active nest was significantly decreased in Lake Vanajanselkä, and the likely explanation is embryotoxicity of dioxin-like compounds. It is plausible that dioxin-like compounds influenced embryonic survival among highly exposed ospreys prior to 2010, especially in Lake Vanajanselkä and Northern Quark. However, decreased survival due to DDE-induced eggshell thinning seems unlikely after 1985, and BDE levels were below those potentially causing adverse effects.

## Introduction

Persistent organic pollutants (POPs) are anthropogenic carbon-based molecules that are very resistant to physical, chemical and biological degradation. They remain intact in the environment for long periods, become widely distributed over long distances, accumulate in living organisms, and biomagnify in the food web. They are also toxic to wildlife and humans and many of them are endocrine disrupters. They have a wide range of common toxic effects, including reproductive and developmental toxicity, neurotoxicity, immunotoxicity and cancer. Because of their harmful effects and high potency, they are banned or strictly regulated by the Stockholm Convention on Persistent Organic Pollutants [1]. Global and national risk management actions have resulted in a general decrease in environmental levels of most POPs. However, due to their slow degradation they remain a cause of concern, and their body burdens may still reach toxic concentrations especially at high trophic levels.

The Baltic Sea is among the most polluted aquatic ecosystems [2]. Because of slow turnover of water, large catchment area and numerous pollution sources the Baltic Sea ecosystem has been greatly affected by pollution from industrial and municipal waste waters, run-offs from agriculture and forestry, and fallout of air emissions. Therefore the concentrations of several POPs are typically higher than in other aquatic ecosystems [3–6].

Polychlorinated dibenzo-*p*-dioxins and -furans (PCDD/Fs) are the most potent class of POPs and together with polychlorinated biphenyls (PCBs) ubiquitously present in the Baltic environment. Certain fish and fishery products, especially fatty fish, still regularly exceed the maximum levels for these compounds established by the European Commission [7, 8]. In addition to PCDD/Fs also coplanar (non-*ortho* and mono-*ortho* substituted) PCBs are included in the highly potent dioxin-like compounds. The common feature of all dioxin-like compounds is their high affinity to the aryl hydrocarbon receptor (AHR), which mediates their characteristic toxic effects, such as developmental defects, endocrine disruption and immunotoxicity [9, 10]. Because different dioxin-like congeners share the same mechanism of toxicity but differ in their potency and toxicokinetics, their toxicity is standardized to the equivalent amount of 2,3,7,8-tetrachloridibenzo-*p*-dioxin (TCDD), the most potent congener compound of dioxin-like congeners, by multiplying the concentration of a congener by its internationally agreed toxic equivalency factor (TEF) [9, 11]. The total dose of dioxin-like compounds in a mixture or the toxic equivalent quantity (TEQ) is calculated by adding up the equivalent amounts of congeners. The most abundant PCB congeners in the environment do not have dioxin-like activity as they are not coplanar due to di-*ortho* substitution. These non-dioxin-like PCBs (NDL-PCBs) are less potent than dioxin-like PCBs, but they are still very persistent and share several toxicological targets with dioxin-like compounds, such as endocrine effects and liver toxicity [12].

Polybrominated biphenyls (PBBs) are brominated analogs of PCBs that have been used as additive flame retardant e.g. in plastics, textiles and building materials since early 1970s [13]. As additives they are not chemically bound to the materials and can therefore easily migrate out from the products to the environment. PBBs are persistent and bioaccumulating, and their effects largely resemble those of PCBs. Exposure and toxic effects of PBBs have been reviewed by the EFSA CONTAM Panel [14] and the IARC [15]. Polybrominated diphenyl ethers (BDEs) are another group of additive flame retardants that have been produced in large quantities worldwide since 1970s [16, 17]. Their critical toxic effects include developmental neurotoxicity and thyroid hormone disruption. Contrary to most other POPs BDE concentrations in the Baltic environment were still increasing until early 1990s but have decreased thereafter as a consequence of efficient risk management actions [4, 5].

Regardless of their widespread occurrence in the environment, food webs and humans polychlorinated naphthalenes (PCNs) are a rather unfamiliar class of POPs [18–20]. They have been manufactured from about 1910 until 1980s and used as electric insulators, lubricants, wood preservation, fungicides, plastic and rubber additives etc. PCNs are also formed as by-products in the synthesis of various chlorinated as well as in municipal waste incineration. Due to planar configuration and structural similarity with PCDD/Fs and dioxin-like PCBs several PCN congeners have the ability to bind to AHR and to elicit dioxin-like toxicity. Their characteristic toxic effects include developmental toxicity, embryotoxicity, immunotoxicity, hepatotoxicity, dermal lesions and carcinogenicity.

The organochlorine insecticide dichlorodiphenyltrichloroethane (DDT) and its main metabolites and environmental degradation products dichlorodiphenyldichloroethylene (DDE) and dichlorodiphenyldichloroethane (DDD) are legacy POPs that are still found everywhere, although their levels are steadily declining [1]. Their best-known toxic effect is eggshell thinning in birds of prey. DDE has also antiandrogenic activity and DDT is a weak estrogen.

Continuous monitoring of temporal trends and spatial distribution of POPs as well as studying their potentially harmful effects on appropriate indicator species are the tools for understanding their environmental behavior and minimizing their harmful effects. Concentrations and trends of POPs in the Baltic biota have been monitored for several decades [3–6, 21–23]. For example, the Swedish Environmental Protection Agency has established the National Programme for Contaminants in Marine Biota for monitoring temporal trends and spatial variation [24]. In addition to tissues of 4 fish species and blue mussel, this Programme utilizes eggs of the common guillemot (*Uria aalgae*), common tern (*Sterna hirundo*) and Eurasian oystercatcher (*Haematopus ostralegus*) for the analysis. Similarly, eggs of the white-tailed sea eagle (*Haliaeetus albicilla*) have been commonly used for environmental monitoring of these contaminants [3, 4, 6, 21–23].

In this study we chose to use the osprey (*Pandion haliaetus*) to characterize temporal trends and spatial distribution of POPs, and to compare the outcome with relevant published data. One of the largest osprey populations of Europe is nesting in Finland [25]. The osprey is a bird of prey that has proved to be an excellent worldwide sentinel species for studying and monitoring contamination of aquatic environments as originally introduced by Grove et al. [26]. It has several characteristics that make it an ideal sentinel species: (1) it is a top predator feeding exclusively on fish, (2) it has localized feeding habits within a reasonably short distance from the nest, (3) it is a long-lived species (up to 25 years) with a strong nest site fidelity returning year after year to the same nesting area, (4) it adapts to potentially contaminated human landscapes and habituates quite readily to human activities, (5) it tolerates short-term nest disturbance and research activities, (6) it nests spatially distributed at regular intervals along waterways at sea, lakes and rivers, (7) it accumulates lipophilic and persistent contaminants, (8) it has a known sensitivity to most environmental contaminants, and (9) it has a nearly worldwide distribution.

The few disadvantages in using the osprey as a sentinel species include the migratory behavior and difficulties in conducting laboratory studies in captivity. Nevertheless, these properties have not prevented the successful collection of valuable datasets. Although the osprey spends about half of the year outside the nesting area, the accumulation of organochlorines and PCDD/Fs into eggs has been shown to mainly reflect exposure from the nesting area, although the possibility of contamination from hotspots in wintering areas is possible [27, 28]. Ring recovery data indicate that the main wintering area of ospreys ringed in Finland is West Africa [29].

A nation-wide program for monitoring osprey populations in Finland (the Project Pandion) was launched in 1971 by Pertti Saurola of the Finnish Ringing Centre [30, 31]. Since then, all known osprey territories have been checked annually by voluntary bird ringers, and unhatched eggs collected and frozen for possible further analysis. In this study we utilized this material stored at the Finnish Museum of Natural History for the analysis of temporal trends of POPs for 46 years (1972–2017) and spatial differences covering two Baltic Sea locations (the Northern Quark in the Gulf of Bothnia and the Finnish Archipelago Sea in SW Finland) and two inland locations in SW Finland, an urban lake area affected by industrial emissions and a pristine area of small lakes. The focus was also on the contribution of the nesting area to the contaminant profiles of eggs, and when possible, on dependence of breeding success on exposure to POPs.

## Materials and methods

### Study areas

**Fig 1:**
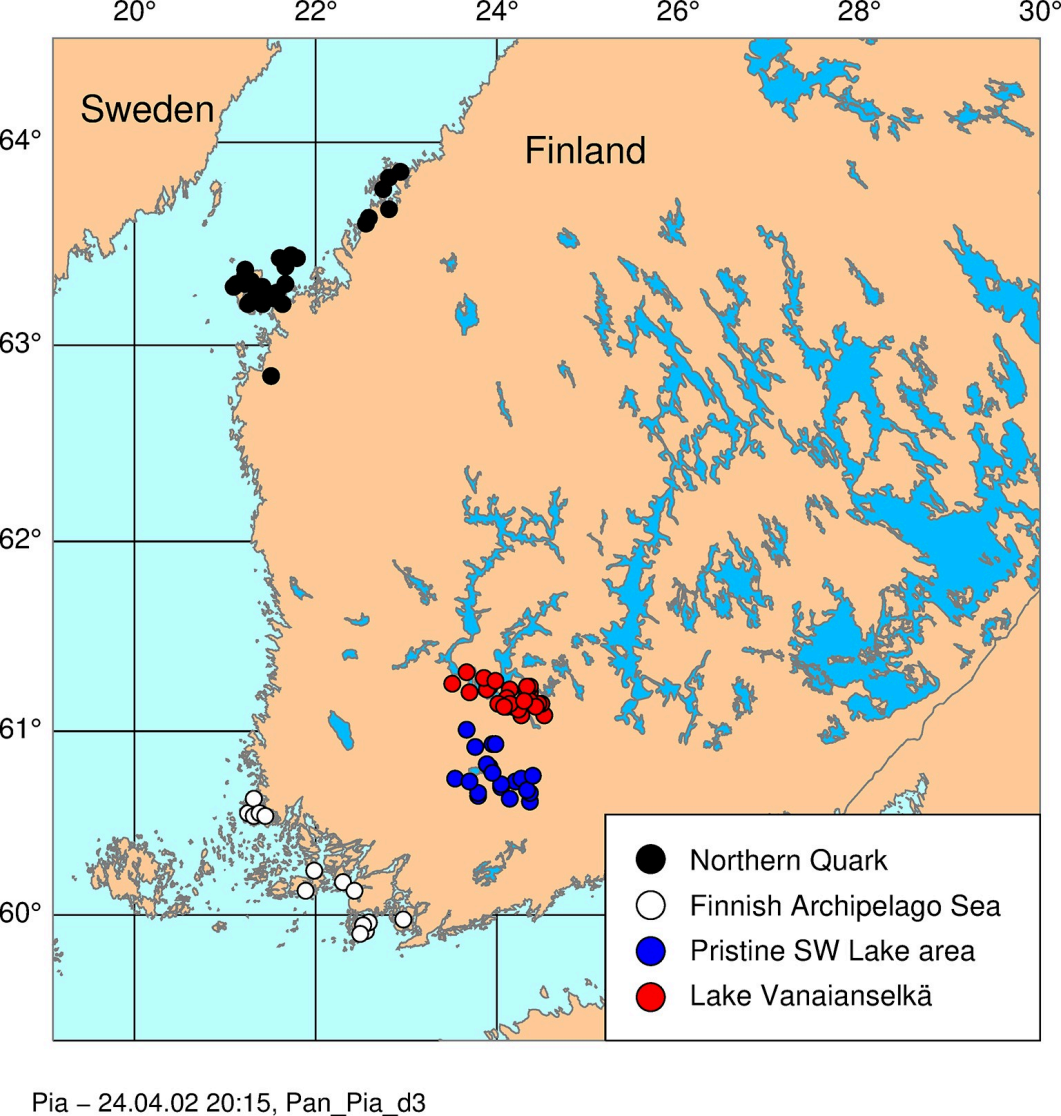
A map indicating study areas and locations of samples. The map was drawn by the PIA application developed by AB based on coordinates from the CIA public domain database.

Two Baltic Sea areas and two inland lake areas were selected for the study based on availability of adequate number of egg samples for time trend analysis. The selected study areas are shown in Fig 1. The Northern Quark is located in the Gulf of Bothnia on the west coast of Finland. The other Baltic Sea study area is the Finnish Archipelago Sea in the SW coast of Finland. The inland study areas in SW Finland are the Lake Vanajanselkä, a large eutrophic-dystrophic lake system affected by long-term direct industrial emissions including PCB emissions originated from coated paper manufacturing more than anywhere else in Finland, and Pristine SW Lake Area, a rural highland area of small lakes and peat bogs. Table 1 indicates the number of samples analyzed in each study area. A complete list of samples indicating the collection year, area and results of analyses is shown in S1 File.

**Table 1:** Number and time frame of collection of osprey eggs analyzed from different study areas.

Study area	Number	Years
Northern Quark	55	1974–2017
Finnish Archipelago Sea	19	1972–2006
Lake Vanajanselkä	46	1972–2017
Pristine SW Lake Area	44	1972–2017
Sum	164	

### Collection of osprey eggs and other observations

Collection of unhatched (addled) eggs was the duty of participating licensed bird ringers when they were checking the occupied territories and ringing the nestlings in the nationwide monitoring Project Pandion led by the Ringing Centre of the Finnish Museum of Natural History and authorized by the Institutional Review Board of the Ministry of Environment (written consent). In Finland, catching and ringing birds are activities regulated by the Nature Conservation Act (1096/1996) and the Hunting Act (615/1993). During the study bird ringing was organized by the Ringing Centre, which also supervises the mandatory training and organizes the licensing exams (https://www.helsinki.fi/en/luomus/observations-and-monitoring/bird-monitoring-and-ringing/bird-ringing). Applicants for a new bird ringing license must be over 18 years of age, have trained in ringing and bird-handling in practice as an assistant to an experienced ringer and received an approved certificate for their training, have completed an approved basic examination that tests their ability to identify species nesting regularly in Finland and the most abundant passage migrants, have submitted an acceptable ringing plan, and be committed to ethical, legal and bird safety guidelines.

The eggs were collected approximately within 4–8 weeks after the normal hatching time (S1 Fig). Variability of embryonic development and possibility for post-hatching microbiological degradation of samples are limitations of the use of unhatched eggs for analysis of non-persistent compounds [32]. Therefore only highly persistent compounds were selected for analyses. Unhatched eggs were weighed, measured and frozen at -20°C for further analysis.

For determination of prey fish species captured by ospreys identifiable leftovers present in nests were counted and recorded during nest inspections and ringing of chicks by M. Finnlund and P. Saurola. Prey fish datasets of 255 samples were available from Northern Quark and 282 samples from the area of Lake Vanajanselkä. Data on breeding success was available from Lake Vanajanselkä and Pristine SW Lake Area. Breeding success was analyzed by recording annually the number of chicks per active nest (in which eggs were laid) and per successful nest (in which large chicks were produced).

Information on the migration routes and wintering areas of ospreys was based on ring encounters extracted from the database of the Finnish Ringing Centre at the Finnish Museum of Natural History. Dependence of migration routes and wintering areas from nesting area was tested by comparing ringing locations in the study areas and locations of ring encounters using the randomization method as described by Lokki & Saurola [33, 34]. The test was carried out separately for encounters from North of Sahara and South of Sahara.

### Chemical analyses

Altogether 164 osprey eggs were homogenized and the concentration of the PCDD/Fs, PCBs, PBBs, BDEs, PCNs as well as DDT and its metabolites (altogether 108 congeners) were analyzed at the Finnish Institute for Health and Welfare with the high-resolution gas chromatography/high resolution mass spectrometer (HRGC/HRMS). The laboratory (T077) is accredited by the Finnish Accreditation Services (FINAS) since 1996. The scope of accreditation includes PCDD/Fs, PCBs and BDEs. The laboratory is also the National Reference Laboratory (NRL) for Halogenated POPs in Feed and Food in Finland. S2 File contains a detailed description of analytical methods, TEF values used for calculation of PCDD/F and PCB TEQs as well as the IUPAC names of numbered PCB, PBB, BDE and PCN congeners.

Analytical results are presented in pg/g wet weight (ww) (PCDD/Fs, dioxin-like PCBs) or in ng/g ww (NDL-PCBs, PBBs, BDEs, PCNs, DDTs). PCDD/F and PCB TEQs were calculated using the WHO_2005_ TEFs [11]. Values below limit of quantification (LOQ) were treated as zero, but for calculation of geometric means they were replaced by the lowest quantified value divided by 2 if <30% of values of the study area were zero.

### Statistical analyses

Trend analyses were carried out using the PIA statistical application developed by Anders Bignert [35] in three steps: (1) Log-linear regression analyses were carried out for the entire study period. The regression line indicates the annual percentage change and r^2^ is the coefficient of determination with a p-value for a two-sided test (H_0_: slope = 0). To avoid exaggerated influence of a single or a few data points at the end of the line (2) the Mann-Kendall trend test [36, 37] was carried out as a non-parametric alternative to the regression analysis resulting in Kendall’s tau (τ) and the corresponding p-value. (3) To identify non-linear trends a 5-point running mean smoother was applied and analysis of variance (ANOVA) was used to test if the smoother explains significantly more than (a) the overall mean concentration (a straight line) and (b) the log-linear regression line, considering the loss of degrees of freedom related to the smoother [38].

In the trend figures, the trend for the whole study period is presented as a red regression line if p < 0.05 (two-sided regression analysis). The smoothed green line is plotted if p < 0.05 (ANOVA) for non-linear trend components. A broken line segment indicates a gap in the time series with a missing year. Abbreviations: n = total n of observations; n(yrs) = total n of years; r^2^ = coefficient of determination (r^2^) together with a p-value for a two-sided test (H0: slope = 0), i.e. a significant value is interpreted as a true change, provided that the assumptions of the regression analysis are fulfilled.

Statistical analysis on breeding success was carried out using Paired T-test and on comparing proportions of prey fish species using Fischer’s Exact test.

## Results

**Fig 2:**
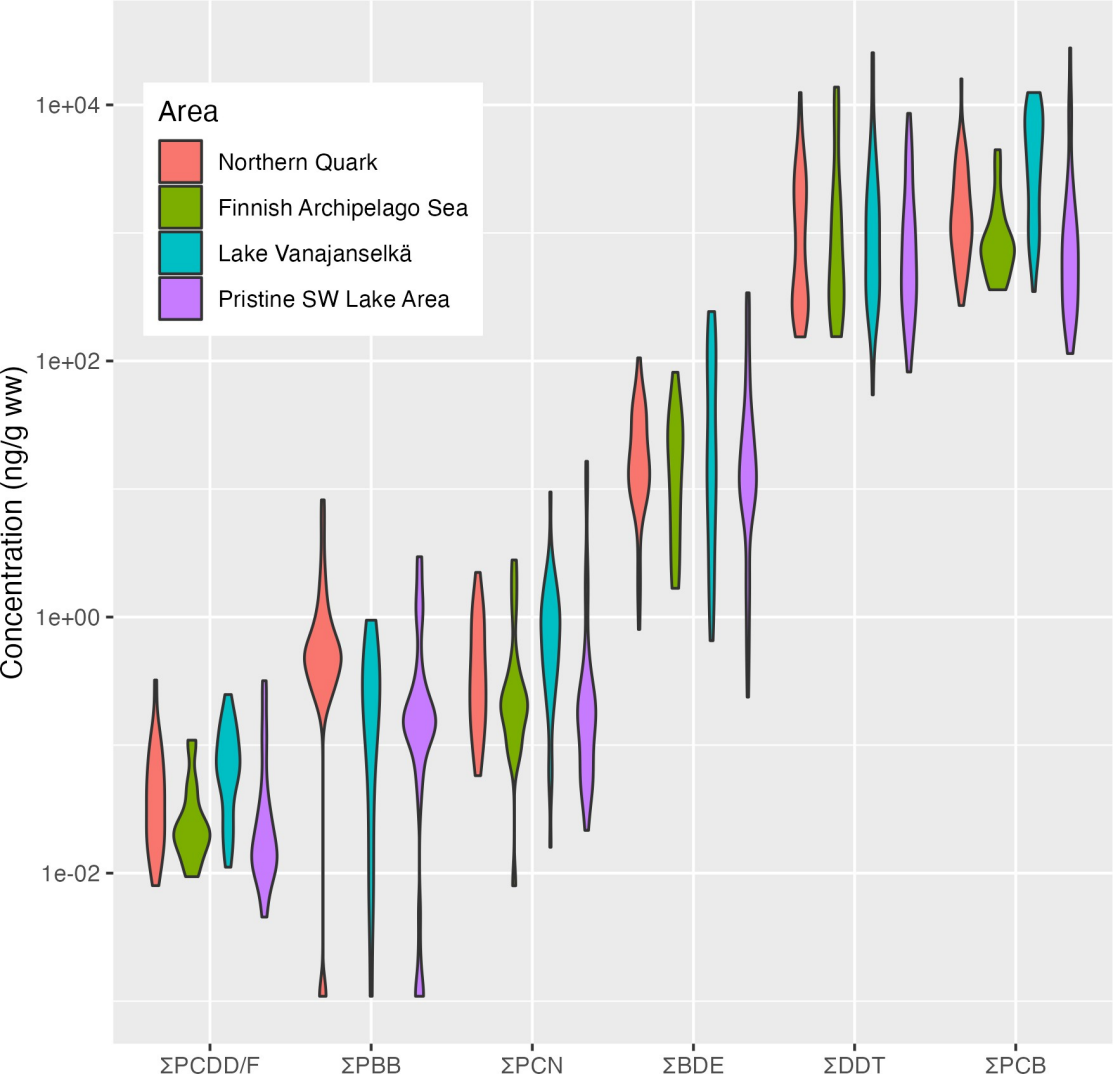
Sum concentrations of POP groups analyzed in osprey eggs from different study areas on a logarithmic scale. The width in the violin graph shows the frequency of observations. The magnitude of concentrations varies up to six orders of magnitude between POP groups and two orders of magnitude within groups.

Sum concentrations of POP groups analyzed from all collected osprey eggs are shown in Fig 2. The data indicates the magnitude differences of concentrations among POP groups. For illustrating the major time related changes, the study period (1972–2017) was divided into the first (1972–1986), second (1987–2001) and third (2002–2017) trimester of the study. Average annual changes of sum concentrations are shown in Table 2. To facilitate comparison with other studies geometric means (GMs) and ranges of POP sum concentrations (and some important congeners) of relevant parts of this study and other studies are shown in Tables 3 and 4.

**Table 2:** Average annual change, 95% confidence interval and significance for sum concentrations of different compound groups. Sampling years 1972–2017 except for PBBs 1972–2005 and for PCNs and DDTs 1972–2006. Statistical significance based on log-linear regression analysis, non-parametric Mann-Kendall trend test (Kendall’s tau) and 5-point running mean smoother (ANOVA) for non-linear trends.

Compound group	Study area	Annual change (%)	95% CI	Log-lin. r^2^ p	Non-param. tau p	Non-lin. smooth. p
∑PCDD/F	All areas	-3.6	-4.6 - -2.5	p<0.001	p<0.001	
	Northern Quark	-4.9	-6.3 - -3.6	p<0.001	p<0.001	p = 0.026
	Finnish Archipelago Sea	-3.7	-6.0 - -1.4	p = 0.004	p = 0.003	
	Lake Vanajanselkä	-2.3	-4.0 - -0.5	p = 0.012	p = 0.009	p = 0.039
	Pristine SW Lake Area	-2.8	-5.4 - -0.1	p = 0.042	p = 0.001	
∑PCDD/F TEQ	All areas	-4.0	-5.0 - -3.0	p<0.001	p<0.001	
	Northern Quark	-5.1	-6.3 - -3.9	p<0.001	p<0.001	p<0.001
	Finnish Archipelago Sea	-3.9	-6.2 - -1.4	p = 0.004	p = 0.003	
	Lake Vanajanselkä	-2.7	-4.5 - -0.83	p = 0.006	p = o.006	
	Pristine SW Lake Area	-3.6	-5.8 - -1.4	p = 0.002	p<0.001	
∑PCB	All areas	-3.5	-4.8 - -2.2	p<0.001	p<0.001	
	Northern Quark	-4.2	-5.6 - -2.7	p<0.001	p<0.001	
	Finnish Archipelago Sea	-3.3	-5.7 - -0.8	p = 0.013	p = 0.004	
	Lake Vanajanselkä	-2.2	-4.4–0.0	p = 0.54	p = 0.41	
	Pristine SW Lake Area	-3.7	-6.6 - -0.7	p = 0.018	p = 0.008	
∑PCB TEQ	All areas	-4.0	-5.2 - -2.8	p<0.001	p<0.001	
	Northern Quark	-4.4	-5.7 - -3.1	p<0.001	p<0.001	p = 0.024
	Finnish Archipelago Sea	-3.7	-6.2 - -1.2	p = 0.007	p = 0.006	
	Lake Vanajanselkä	-2.8	-4.9 - -0.6	p = 0.013	p = 0.012	
	Pristine SW Lake Area	-4.2	-6.7 - -1.5	p = 0.003	p<0.001	
∑TEQ	All areas	-4.0	-5.2 - -2.8	p<0.001	p<0.001	
	Northern Quark	-4.6	-5.8 - -3.3	p<0.001	p<0.001	p = 0.021
	Finnish Archipelago Sea	-3.7	-6.2 - -1.3	p = 0.006	p = 0.002	
	Lake Vanajanselkä	-2.8	-4.9 - -0.8	p = 0.009	p = 0.012	
	Pristine SW Lake Area	-4.1	-6.5 - -1.5	p = 0.003	p<0.001	
∑PBB	All areas	5.6	3.1–8.1	(+++)	(+)	p<0.001
	Northern Quark	-1.4	-3.4–0.6			p = 0.107
	Lake Vanajanselkä	8.7	5.7–11.8	(+++)	(+++)	p = 0.012
	Pristine SW Lake Area	6.5	2.8–10.4	(+++)	(+)	p = 0.011
∑BDE	All areas	6.6	5.0–8.3	(+++)	(+++)	p<0.001
	Northern Quark	3.9	1.9–6.0	(+++)	(++)	p<0.001
	Finnish Archipelago Sea	7.4	3.4–11.5	(++)	(+)	p = 0.006
	Lake Vanajanselkä	10.0	6.8–13.2	(+++)	(+++)	p<0.001
	Pristine SW Lake Area	6.3	2.2–10.5	(++)	(+)	p<0.001
∑PCN	All areas	-4.0	-6.1 - -1.9	p<0.001	p<0.001	
	Northern Quark	-2.7	-6.0–0.77	p = 0.117	p = 0.126	
	Finnish Archipelago Sea	-3.7	-7.6–0.24	p = 0.064	p = 0.030	
	Lake Vanajanselkä	-7.0	-16.1–3.1	p = 0.137	p = 0.063	
	Pristine SW Lake Area	-5.7	-9.8 - -1.3	p = 0.013	p = 0.002	
∑DDT	All areas	-8.1	-9.2 - -7.0	p<0.001	p<0.001	
	Northern Quark	-9.1	-10.5 - -7.7	p<0.001	p<0.001	
	Finnish Archipelago Sea	-9.5	-12.6 - -6.4	p<0.001	p<0.001	
	Lake Vanajanselkä	-7.1	-9.5 - -4.6	p<0.001	p<0.001	
	Pristine SW Lake Area	-7.2	-10.0 - -4.4	p<0.001	p<0.001	

Key: For increasing trends (+) p<0.05; (++) p<0.01; (+++) p<0.001.

**Table 3:** Comparison of ∑PCDD/F, PCDD/F TEQ, ∑PCB, PCB TEQ and ∑TEQ concentrations in eggs analyzed in this study and other relevant studies (pg/g ww except for ∑PCB ng/g ww, geometric mean and range, unless otherwise stated).

Species	Country, area	Year	n	∑PCDD/F	PCDD/F TEQ	∑PCB	PCB TEQ	∑TEQ	Reference
Osprey	Finland, all areas	1972–2017	164	35.3 4.54–322	12.1 1.14–109	1381 114–27851	57.1 4.85–885	70.0 5.99–938	This study
Osprey	Finland, all areas	1972–1986 trimester 1	55	53.0 11.1–160	19.8 3.83–66.3	2169 230–27851	96.6 8.35–885	118 12.2–938	This study
Osprey	Finland, Northern Quark	1972–1986 trimester 1	20	67.2 18.5–301	26.6 8.25–55.9	2383 755–5859	112 36.8–235	139 45.0–266	This study
Osprey	Finland, Northern Quark	1987–2001 trimester 2	14	49.4 14.6–322	15.9 5.89–91.2	1412 317–15966	62.4 15.8–436	78.8 23.7–527	This study
Osprey	Finland, Finnish Archipelago Sea	1987–2001 trimester 2	6	32.0 17.0–109	13.1 6.96–48.6	1084 548–4464	48.7 24.0–216	61.9 31.9–265	This study
Osprey	Finland, Lake Vanajanselkä	1987–2001 trimester 2	14	100.0 47.4–248	34.4 14.4–92.2	4453 677–11145	174 30.0–568	211 44.5–646	This study
Osprey	Finland, Pristine SW Lake Area	1987–2001 trimester 2	26	24.1 4.54–318	7.60 2.06–109	887 129–10452	34.1 129–10452	41.9 7.44–395	This study
White-tailed sea eagle	Sweden, Baltic Proper	1992–2004	8	238 145–567		28440 13860–58260	2166	1772 984–3468	Nordlöf et al., 2012 [39]
White-tailed sea eagle	Denmark, Greenland	2000	3	24.4 20.3–30.2		4197 3515–5127	272	247 198–312	Nordlöf et al., 2012 [39]
Osprey	Spain, Menorca	1994–2000	9	6.27 2.64–14.22		4159 942–15027		55.7 16.0–136	Jiménez et al., 2007 [40]
Osprey	USA, Oregon, Willamette River	1993	10	1713	14.5 8.78–23.99				Henny et al., 2009 [41]
Osprey	USA, Oregon, Willamette River	2001	4–11	309		245 and 1460			Henny et al., 2009 [41]
Osprey	USA, lower Columbia River	1997–1998	29		13.9	1435	50.0	62.5	Henny et al., 2008 [42]
Osprey	USA, lower Columbia River	2004	40		6.44 1.40–52.3	783 159–8419	36.7 6.64–866	43.8 9.86–910	Henny et al., 2008 [42]
Osprey	USA, Wisconsin, polluted area	1992–1996	9–18		71–119^*a*^ 52–171	1586–1666^*a*^ 809–2745	31–41^*a*^ 23–59		Woodford et al., 1998 [43]
Osprey	USA, Wisconsin, reference area	1992–1996	3–6		4–8^*a*^ 0.4–27	478–482^*a*^ 200–1694	24 9–47		Woodford et al., 1998 [43]
Thick-billed murre	Canada, Prince Leopold Island	1975–2014	10-15/y	9.0–29.4^*b*^	5.32–20.5^*b*^		6.97–44.8^*b*^^*c*^	12.3–65.3^*b*^^*c*^	Braune & Mallory; 2017 [44]
Northern fulmar	Canada, Prince Leopold Island	1975–2014	15/y	15.5–112^*b*^	9.75–65.4^*b*^		5.77–49.4^*b*^^*c*^	15.5–115^*b*^^*c*^	Braune & Mallory; 2017 [44]
White-tailed sea eagle	Sweden, Baltic coast	1970–1974	27			55880^*c*^ 47752–66040			Helander et al., 2002 [45]
White-tailed sea eagle	Sweden, Baltic coast	1995–1997	5			19812^*c*^ 13208–29972			Helander et al., 2002 [45]
Osprey	Norway, Oslo Fjord and inland	1991–1997	5			5458^*d*^ ND^*e*^–12927			Herzke et al., 2002 [32]
Osprey	Canada, Fraser River	1991–1997	51			104–436^*a*^ 45.4–1104			Elliott et al., 2000 [46]
Osprey	USA, Columbia River	1991–1997	60			343–2360^*a*^ 149–11952			Elliott et al., 2000 [46]
Osprey	Canada, Great Lakes Basin	1991–1995	77			1650–7089^*a*^ 251–26542			Martin et al., 2003 [47]
Osprey	USA, Chesapeake Bay, polluted area	2000–2001	45			3600–9280^*a*^ 1350–19300			Rattner et al., 2004 [48]
Osprey	USA, Chesapeake Bay, reference area	2000–2001	30			4300–4910^*a*^ 2310–12400			Rattner et al., 2004 [48]
Osprey	USA, Delaware River and Bay	2002	39			1440–8680^*a*^ 469–14500			Toschik et al., 2005 [49]
White-tailed sea eagle	Norway, Oslo Fjord and inland	1991–1997	7			8955^*d*^ 3078–13038			Herzke et al., 2002 [32]
Guillemot	Sweden, Baltic, Stora Karlsö	1969				46080 39480–53880^*f*^			Bignert et al., 1998 [22]
Guillemot	Sweden, Baltic, Stora Karlsö	1995	30?			4440 3840–5160^*f*^			Bignert et al., 1998 [22]

^*a*^range of geometric means

^*b*^range of annual means

^*c*^non-ortho PCBs only

^*d*^mean or range of means

^*e*^ND = non-detectable

^*f*^95% confidence interval

**Table 4:** Comparison of ∑PBB, ∑BDE, ∑PCN and ∑DDT concentrations and concentrations of some congeners in eggs analyzed in this study and other relevant studies (ng/g ww, geometric mean and range, unless otherwise stated).

**A. ∑PBB**								
**Species**	**Country, area**	**Year**	**n**	**∑PBB**	**PBB 153**	**PBB 154**	**PBB 155**	**Reference**
Osprey	Finland, all areas	1972–2005	115	0.162 ND^*a*^–8.23	0.029^*b*^ ND-0.761	0.036^*b*^ ND-8.23	0.087^*b*^ ND-9.52	This study
Osprey	Finland, all areas	1972–1986 trimester 1	42	0.058 ND-1.79				
Osprey	Finland, all areas	1987–2001 trimester 2	54	0.357 ND-8.23				
Osprey	Finland, all areas	2001–2005 trimester 3	19	0.166 ND-0.531				
Osprey	Finland, Northern Quark	1987–2001 trimester 2	14	0.756 0.174–8.23	0.130 0.031–0.615	0.142 0.050–0.704	0.322 0.055–6.57	This study
Osprey	Norway, Oslo Fjord, inland	1991–2000	5	~1^*b*^	0.2^*b*^ ND-12			Herzke et al., 2005 [50]
White-tailed sea eagle	Norway, coast	1992–2000	12	15^*b*^	9.4^*b*^ 4–34			Herzke et al., 2005 [50]
White-tailed sea eagle	Norway, coast	1992–2000	7		6.1^*b*^ 3.8–34	3.2^*b*^ 1.9–12	3.6^*b*^ <0.1–11.5	Vetter et al., 2008 [51]
White-tailed sea eagle	Sweden, different areas	1992–2005	44		1.0–6.0^*c*^ 0.45–21.5			Nordlöf et al., 2010 [52]

**B. ∑BDE**						
**Species**	**Country, area**	**Year**	**n**	**∑BDE**	**BDE 47**	**Reference**
Osprey	Finland, all areas	1972–2017	164	15.0 0.24–341	9.85 0.21–237	This study
Osprey	Finland, Northern Quark	1987–2001 trimester 2	14	32.9 13.6–90.2	21.1 13.6–90.2	This study
Osprey	Finland, Finnish Archipelago Sea	1987–2001 trimester 2	6	27.5 8.78–81.6	19.7 6.59–56.8	This study
Osprey	Finland, Lake Vanajanselkä	1987–2001 trimester 2	14	92.0 19.9–243	62.7 13.2–162	This study
Osprey	Finland, Pristine SW Lake Area	1987–2001 trimester 2	26	27.4 5.84–341	18.1 3.69–237	This study
Osprey	Norway, Oslo Fjord, inland	1993–2000	5	103^*b*^ 21–254	55.1^*b*^ 10–135	Herzke et al., 2005 [50]
White-tailed sea eagle	Norway, coast	1992–2000	12	184^*b*^ 46–804	110^*b*^ 23–411	Herzke et al., 2005 [50]
Osprey	Sweden	1966–2013	?		16^*b*^ 0.31–190	Geng et al., 2016 [53]
White-tailed sea eagle	Sweden, different areas	1992–2005	44	36.0–215^*c*^ 10.5–680	14.0–130^*c*^ 4.0–420	Nordlöf et al., 2010 [52]
Osprey	USA, Chesapeake Bay, polluted areas	2000–2001	9	195–725^*c*^ 154–928	114–480^*c*^ 95.8–647	Rattner et al., 2004 [48]
Osprey	USA, Chesapeake Bay, reference areas	2000–2001	6	176–253^*c*^ 87.0–397	114–156^*c*^ 54.4–190	Rattner et al., 2004 [48]
Osprey	USA, Delaware River and Bay	2002	15	82.2–572^*c*^ 70.9–820	46.6–276^*c*^ 43.0–453	Toschik et al., 2005 [49]
Osprey	USA, Columbia, Yakima and Willamette Rivers	1991–2000	89	97.7–897^*c*^ 48.7–1880	54.0–562^*c*^ 30.1–1020	Henny et al., 2009 [54]

**C. ∑PCN**					
**Species**	**Country, area**	**Year**	**n**	**∑PCN**	**Reference**
Osprey	Finland, all areas	1972–2006	82	0.193 0.012–16.5	This study
Osprey	Finland, all areas	1972–1986 trimester 1	25	0.340 0.012–16.46	This study
Osprey	Finland, all areas	1987–2001 trimester 2	32	0.200 0.022–9.77	This study
Osprey	Finland, all areas	2001–2006 trimester 3	25	0.104 0.024–1.22	This study
White-tailed sea eagle	Sweden, Baltic Proper	1985–1989	2	6.10–6.60^*d*^	Järnberg et al., 1997 [55]
Guillemot	Sweden, Gotland	1974–1987	50^*e*^	10.0–26.2^*d*^	Järnberg et al., 1997 [55]
Thick-billed murre	Canada, Prince Leopold Island	1975–2014	9–15^*e*^/y	0.36–1.00^*f*^	Braune & Muir, 2017 [56]
Thick-billed murre	Canada, Prince Leopold Island	1975–1998	9–15^*e*^/y	0.75–1.00^*f*^	Braune & Muir, 2017 [56]
Thick-billed murre	Canada, Prince Leopold Island	2003–2006	15^*e*^/y	0.49–0.74^*f*^	Braune & Muir, 2017 [56]

**S. ∑ DDT, DDE, DDD, DDT**								
**Species**	**Country, area**	**Year**	**n**	**∑DDT**	***p*,*p’*-DDE**	***p*,*p’*-DDD**	***p*,*p’*-DDT**	**Reference**
Osprey	Finland, all areas	1972–2006	147	804 54.4–25561	713 54.4–24105	71.7 ND-2782	ND ND-117	This study
Osprey	Finland, all areas	1972–1986 trimester 1	54	2391 344–25561	2023 276–24105	296 44.3–2782	ND ND-117	This study
Osprey	Finland, all areas	1987–2001 trimester 2	60	570 132–5053	514 114–4247	48.4 6.28–799	ND ND-22.0	This study
Osprey	Finland, all areas	2002–2006 trimester 3	33	253 54.4–570	234 54.4–526	14.5 ND-72.4	ND ND	This study
Osprey	Sweden	1966–2013	?		540^*b*^ 39–31000	25^*b*^ 1.5–2100		Geng et al., 2016 [53]
Osprey	Finland, Northern Quark	1971–1972	6		3000–27000^*d*^			Koivusaari et al., 1972 [21]
White-tailed sea eagle	Sweden, Baltic coast	1970–1974	27	36068 29972–42672^*e*^				Helander et al., 2002 [45]
White-tailed sea eagle	Sweden, Baltic coast	1995–1997	5	5588 3556–8182^*e*^				Helander et al., 2002 [45]
Osprey	Spain, Menorca	1994–2000	9		341 57–1016		17.83 9–50	Jiménez et al., 2007 [40]
Osprey	USA, Wisconsin	1992–1996	21		197–521^*c*^ 79–939		1.6–5^*c*^ 0.5–21	Woodford et al., 1998 [43]
Osprey	Canada, Great Lakes Basin	1991–1995	77		1285–2904^*d*^ 256–8636	72.0–308^*d*^ 1.30–2853	3.75–53.8^*d*^ ND-545	Martin et al., 2003 [47]
Osprey	Canada, Fraser River	1991–1997	51		706–5430^*a*^ 246–13900	14.7–663^*c*^ 3.0–1230	ND-27.6^*c*^ ND-682	Elliott et al., 2000 [46]
Osprey	USA, Columbia River	1991–1997	60		1010–13800^*a*^ 320–22900	34.7–1376^*c*^ 3–1485	2.7–89.7^*c*^ ND-263	Elliott et al., 2000 [46]
Osprey	USA, lower Columbia River	1997/8	29		4872	199	19.8	Henny et al., 2008 [42]
Osprey	USA, lower Columbia River	2004	40		1521 985–2294	69.7 23.9–281	4.92 0.74–43.9	Henny et al., 2008 [42]
Osprey	USA, Oregon, Willamette River	1993 and 2001	10, 11		2347 and 1353	98.5 and 29.4	25.3 and 2.08	Henny et al., 2009 [41]
Osprey	USA, Chesapeake Bay, polluted areas	2000–2001	45		443–1160^*c*^ 258–1920	86–129^*c*^ 53–240		Rattner et al., 2004 [48]
Osprey	USA, Chesapeake Bay, reference areas	2000–2001	30		404–466^*c*^ 238–984	39–40^*c*^ 15–131		Rattner et al., 2004 [48]
Osprey	USA, Delaware River and Bay	2002	39		352–1770^*c*^ 172–4610	17–182^*c*^ 6–323	3–4^*c*^ ND-16	Toschik et al., 2005 [49]

^*a*^ND = non-detectable

^*b*^median

^*c*^range of geometric means

^*d*^mean and range

^*e*^pooled samples

^*f*^range of annual means

### PCDD/Fs

**Fig 3:**
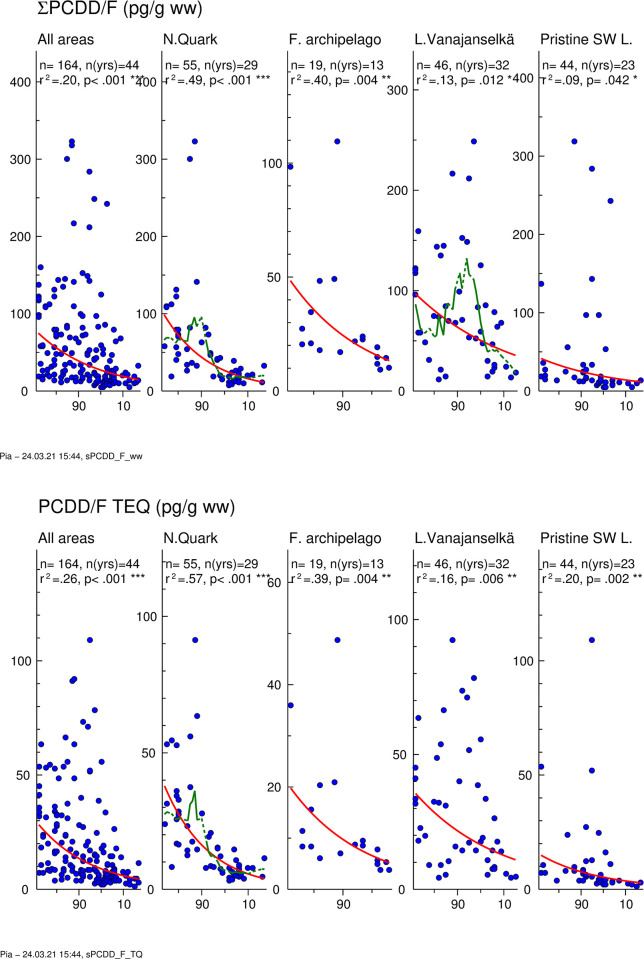
Temporal trend (1972–2017) of ∑PCDD/F (upper graph) and PCDD/F TEQ (lower graph) in osprey eggs in the whole dataset and in different study areas. Blue circles indicate individual data, and a red regression line is shown if p<0.05 (two-sided regression analysis). A smoothed green line is shown for non-linear trend components if p < 0.05 (ANOVA). There was a significant decrease in the whole dataset (annual decrease 3.6%) and in all study areas (annual decrease 2.3–4.9%).

**Fig 4:**
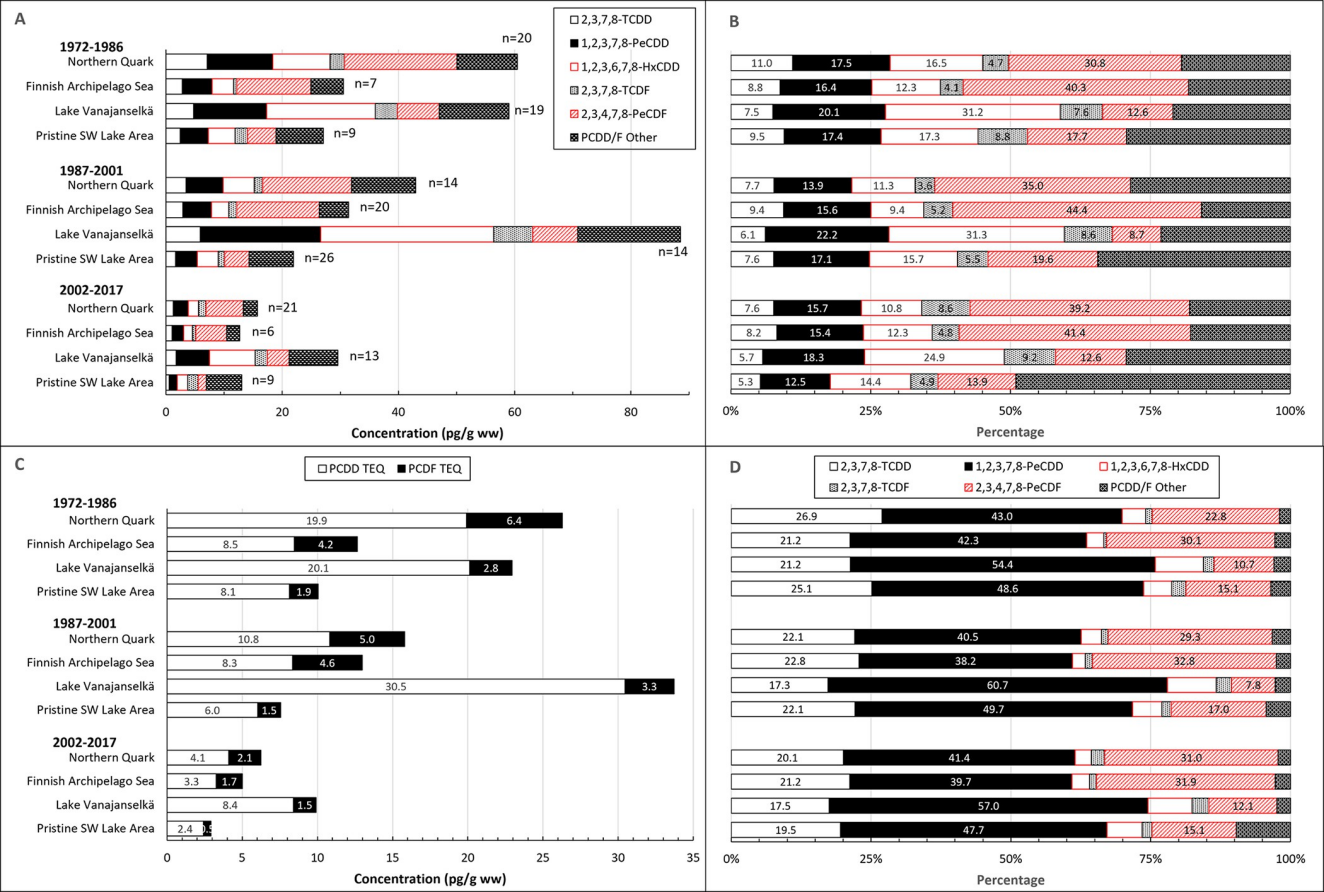
Geometric mean concentrations of 5 most abundant PCDD/F congeners and the sum of other congeners (A), and PCDD and PCDF TEQ (C) in different study areas during the first (1972–1986), second (1987–2001) and third (2002–2017) trimester of the study. Right panel shows the average relative proportion of PCDD/F congeners of ∑PCDD/F concentrations (B) and PCDD/F congener TEQs of PCDD/F TEQ (D). Congener profile was quite similar in both Baltic Sea areas: 2,3,4,7,8-PeCDF was the dominant congener followed by 1,2,3,7,8-PeCDD and 1,2,3,6,7,8-HxCDD. In Lake Vanajanselkä 1,2,3,6,7,8-HxCDD was the main congener followed by 1,2,3,7,8-PeCDD. In Pristine SW Lake Area 4 congeners (2,3,4,7,8-PeCDF, 1,2,3,7,8-PeCDD, 1,2,3,6,7,8-HxCDD and OCDD) had nearly similar proportions. 1,2,3,7,8-PeCDD contributed most to the PCDD/F TEQ in all study areas followed by 2,3,4,7,8-PeCDF in the Baltic areas and by 2,3,7,8-TCDD in the lake areas.

Temporal trends of ∑PCDD/F are presented in Fig 3. GMs of absolute concentrations and average relative concentrations of the 5 most abundant PCDD/F congeners and TEQs in different study areas are shown in Fig 4. Individual data on ∑PCDD/F and PCDD/F-TEQs of all samples from different years and study areas are shown in S2 Fig.

∑PCDD/F and PCDD/F TEQ levels of all samples (1972–2017) followed the same pattern in all study areas, and the order of GMs was Lake Vanajanselkä 61.3 and 20.8 pg/g ww, respectively > Northern Quark 36.7 and 13.5 pg/g ww > Finnish Archipelago Sea 24.1 and 9.59 pg/g ww > Pristine SW Lake Area 22.3 and 6.65 pg/g ww (Fig 4 and S2 Fig). The highest concentrations of individual eggs were in Northern Quark and Pristine SW Lake Area. There was a statistically significant decreasing trend in the whole dataset for both ∑PCDD/F and PCDD/F TEQ (annual decrease 3.6 and 4.0%, respectively) and in all study areas (Fig 3, Table 2). The fastest decrease was in Northern Quark and slowest in Lake Vanajanselkä.

PCDD/F congener profile was greatly dependent on study area (Fig 4). 2,3,4,7,8-PeCDF was the dominant congener in both Baltic Sea areas followed by 1,2,3,7,8-PeCDD > 1,2,3,6,7,8-HxCDD. The profile did not change much over the study period except that the proportion of 2,3,4,7,8-PeCDF showed some increases and 1,2,3,6,7,8-HxCDD decreases. Congener profiles were different in inland study areas. In Lake Vanajanselkä 1,2,3,6,7,8-HxCDD was the main congener followed by 1,2,3,7,8-PeCDD > 2,3,4,7,8-PeCDF, and in Pristine SW Lake Area 2,3,4,7,8-PeCDF, 1,2,3,7,8-PeCDD, 1,2,3,6,7,8-HxCDD and OCDD were the most abundant congeners with rather similar proportions.

PCDDs dominated the PCDD/F TEQ in all study areas, and their proportion of PCDD/F TEQ was higher in the lake areas (Lake Vanajanselkä 86.3%, Pristine SW Lake Area 80.0%) than in the Baltic Sea areas (Northern Quark 69.7%, Finnish Archipelago Sea 65.3%). 1,2,3,7,8-PeCDD contributed most to PCDD/F TEQ in all study areas, and its proportion was higher in Lake Vanajanselkä and Pristine SW Lake Area than in both Baltic Sea areas. Contribution of 2,3,4,7,8-PeCDF was the second in Northern Quark and Finnish Archipelago Sea followed by 2,3,7,8-TCDD. The contribution order of these two congeners was reversed in the lake areas. Relative TEQ proportions were quite constant over the study period.

Contribution of dioxin-like activity to ∑PCDD/F is shown in S3 Fig, which indicates linear regression between PCDD/F TEQ and ∑PCDD/F in different study areas. PCDD/F TEQs contributed most to ∑PCDD/F in Finnish Archipelago Sea (R^2^ = 0.98) followed by Lake Vanajanselkä (R^2^ = 0.88) and Northern Quark (R^2^ = 0.81). The contribution was poor in Pristine SW Lake Area (R^2^ = 0.44).

### PCBs

**Fig 5:**
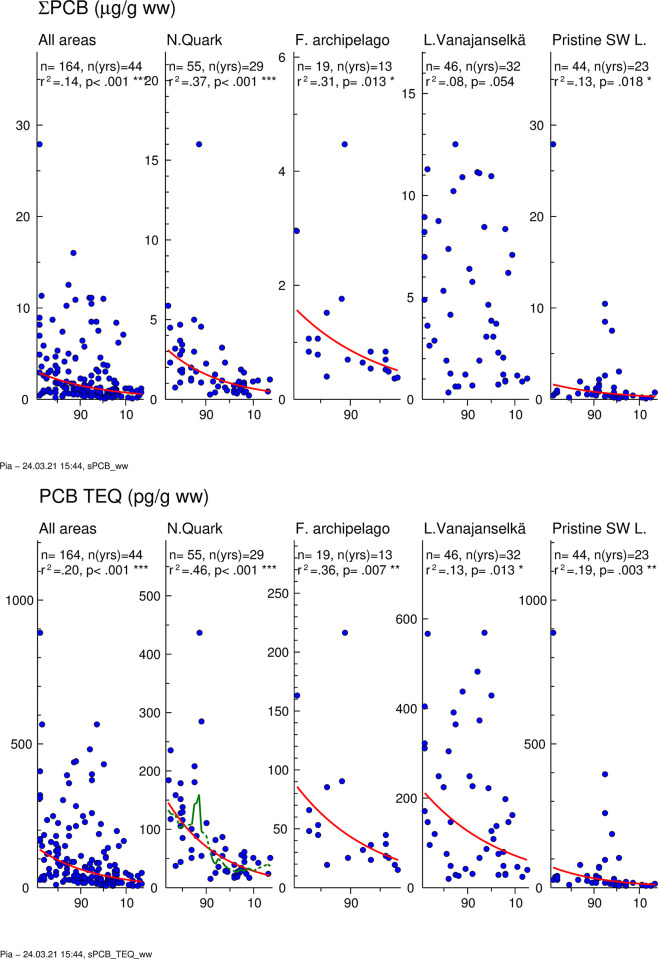
Temporal trend (1972–2017) of ∑PCB (upper graph) and PCB TEQ (lower graph) in osprey eggs in the whole dataset and in different study areas. Blue circles indicate individual data, and a red regression line is shown if p<0.05 (two-sided regression analysis). A smoothed green line is shown for non-linear trend components if p < 0.05 (ANOVA). Both ∑PCB and PCB TEQ decreased significantly in the whole dataset (annual decrease 3.5 and 4.0%, respectively) and in all study areas (annual decrease 2.2–4.4%).

**Fig 6:**
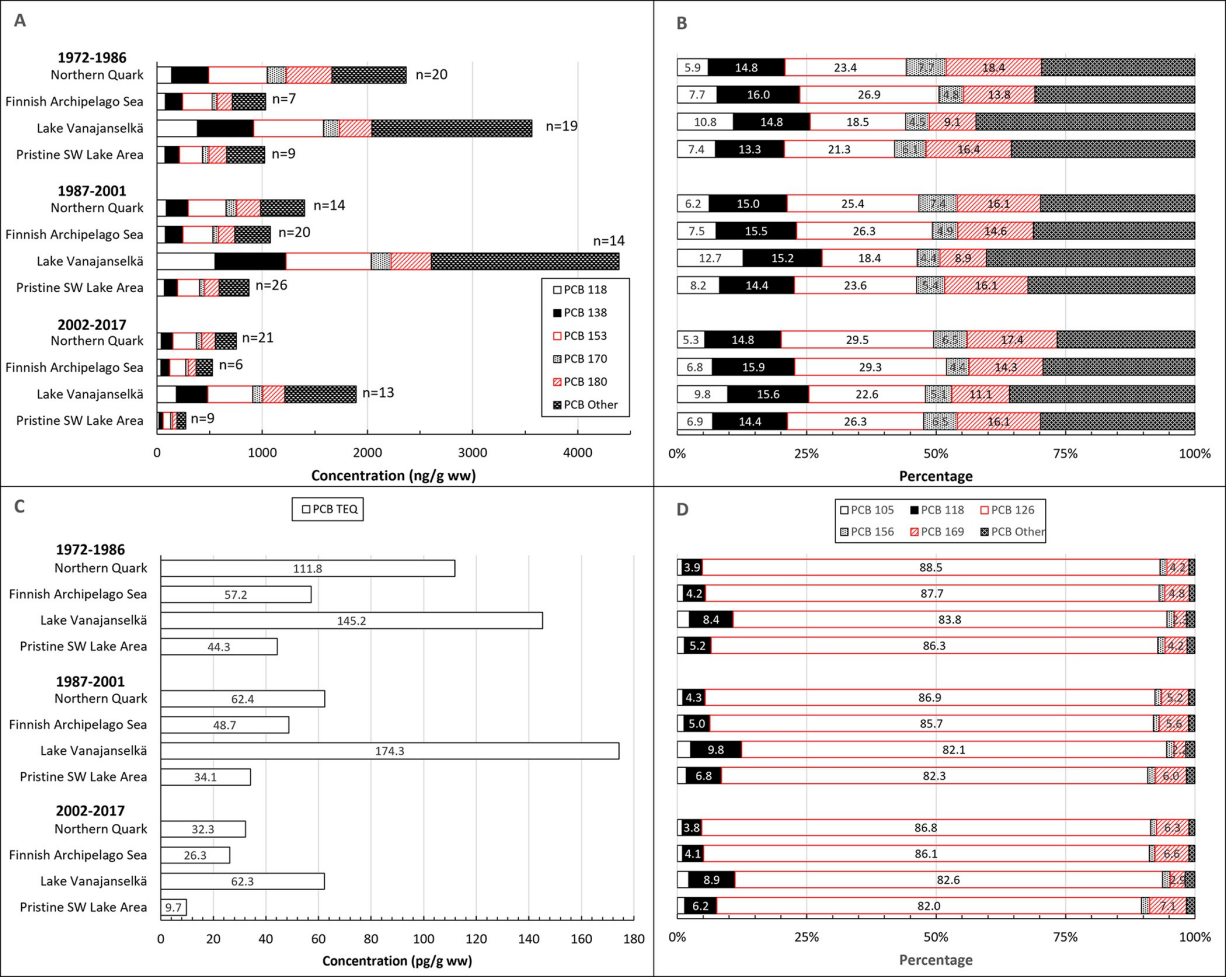
Geometric mean concentrations of 5 most abundant PCB congeners and the sum of other congeners (A), and PCB TEQ (C) in different study areas during the first (1972–1986), second (1987–2001) and third (2002–2017) trimester of the study. Right panel shows the average relative proportion of PCB congeners of ∑PCB concentrations (B) and PCB congener TEQs of PCB TEQ (D). Congener profile was quite similar in all study areas and did not change over time. PCB 153 was the dominant congener followed by PCB 180 / PCB 138. PCB 126 contributed most to PCB TEQ followed by PCB 169 / PCB 118.

Temporal trends of ∑PCB are presented in Fig 5. GMs of absolute concentrations and average relative concentrations of the 5 most abundant PCB congeners and TEQs in different study areas are shown in Fig 6. Individual data on ∑PCB and PCB-TEQs of all samples from different years and study areas are shown in S4 Fig.

∑PCB and PCB TEQs of all samples (1972–2017) followed the same pattern (Fig 6 and S4 Fig), and the order of GMs was Lake Vanajanselkä 3227 ng/g ww and 121 pg/g ww, respectively > Northern Quark 1349 ng/g ww and 60.0 pg/g ww > Finnish Archipelago Sea 850 ng/g ww and 42.5 pg/g ww > Pristine SW Lake Area 721 ng/g ww and 27.8 pg/g ww. Temporal trends showed a significant decrease for both ∑PCB and PCB TEQ in the whole dataset (annual decrease 3.5 and 4.0%, respectively) and in all study areas, except that in Lake Vanajanselkä there was no significant trend for ∑PCB (Fig 5, Table 2). The fastest decrease was in Northern Quark (4.2 and 4.4%) and slowest for PCB TEQ in Lake Vanajanselkä (2.8%).

PCB 153 was the most abundant congener in all study areas. It was followed by PCB 180 > PCB 138 in Northern Quark and Pristine SW Lake Area, but the order of these two congeners was reversed in Finnish Archipelago Sea and in Lake Vanajanselkä (Fig 6). There were no major changes in congener profile over time. Of the TEQ profiles PCB 126 had by far the greatest and nearly equal contribution to PCB TEQ in all study areas (82.0–88.5%) followed by PCB 169 > PCB 118 in the Baltic Sea areas. The order of these two congeners was reversed in the lake areas.

Correlation between ∑PCB and ∑indicator PCB is shown in S5 Fig, and between PCB TEQ and ∑PCB in S6 Fig. The correlation between ∑PCB and ∑indicator PCB was almost perfect in all study areas (R^2^ = 0.99–1.0) denoting the general ability of indicator PCBs to predict total PCB exposure. Correlation between ∑PCB and PCB TEQ was very strong in Pristine SW Lake Area (R^2^ = 0.99) and in Finnish Archipelago Sea (R^2^ = 0.98), and good correlation in Lake Vanajanselkä (R^2^ = 0.86) and Northern Quark (R^2^ = 0.86).

### ∑TEQ

**Fig 7:**
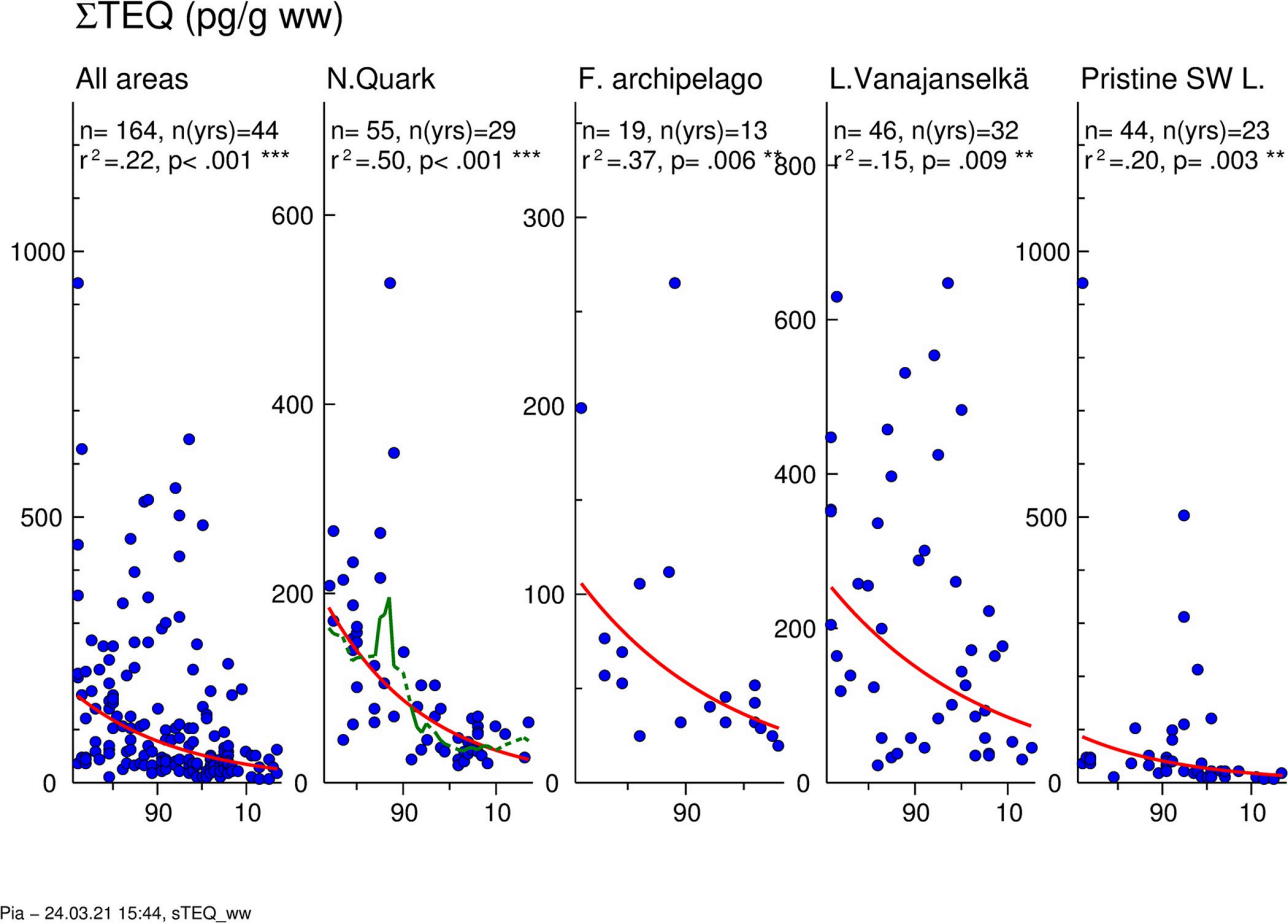
Temporal trend of ∑TEQ in osprey eggs in different study areas. Blue circles indicate individual data, and a red regression line is shown if p<0.05 (two-sided regression analysis). A smoothed green line is shown for non-linear trend components if p < 0.05 (ANOVA). ∑TEQ decreased significantly in the whole dataset (annual decrease 4.0%) and in all study areas (annual decrease 2.8–4.6%).

**Fig 8:**
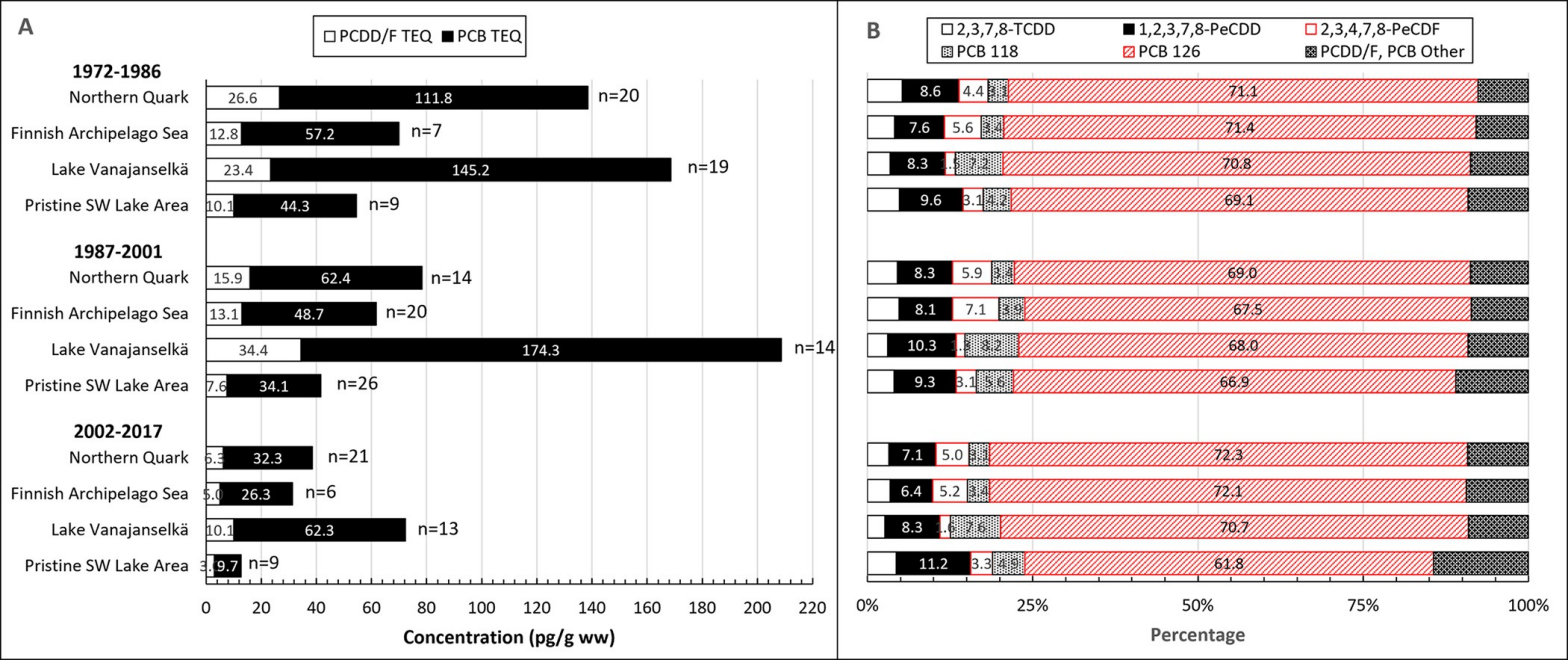
Geometric means of ∑TEQs (A) and average relative (B) concentrations in different study areas during the first (1972–1986), second (1987–2001) and third (2002–2017) trimester of the study. PCB 126 was the dominant congener of ∑TEQ in all study areas followed by 1,2,3,7,8-PeCDD > PCB 118 > 2,3,4,7,8-PeCDF > 2,3,7,8-TCDD > PCB 169 > PCB 105.

Total (PCDD/F+PCB) TEQs reflect the overall dioxin-like activity of samples. Temporal trends of ∑TEQ are shown in Fig 7. For congener profiles, GMs of ∑TEQ and average proportions of 5 most abundant congeners in different study areas are shown in Fig 8. All individual values of the sum of ∑TEQ are shown in S7 Fig and proportion PCB TEQ of ∑TEQ in S8 Fig.

The pattern of ∑TEQ was similar to that of PCB TEQ due to the dominating role of PCB TEQs. The order of GMs of all samples (1972–2017) was Lake Vanajanselkä 144 pg/g ww > Northern Quark 74.0 pg/g ww > Finnish Archipelago Sea 52.4 pg/g ww > Pristine SW Lake Area 34.9 pg/g ww (Fig 8 and S6 Fig). However, the highest concentrations in individual eggs were in Pristine SW Lake Area. On average, PCBs contributed 81.8% and PCDDs 14.0% and PCDFs 4.2% of ∑TEQ (S8 Fig). ∑TEQ decreased significantly in the whole dataset (annual decrease 4.0%) and in all study areas (Fig 7, Table 2). The fastest decrease was in Northern Quark (4.6%) and the slowest in Lake Vanajanselkä (2.8%).

There were only minor differences in the congener profile of dioxin-like compounds between study areas and over time (Fig 8). PCB 126 had by far the greatest and quite similar contribution to ∑TEQ in all study areas (61.8–72.3%) followed by 1,2,3,7,8-PeCDD (6.4–11.2%) > PCB 118 (3.1–8.2%) > 2,3,4,7,8-PeCDF > 2,3,7,8-TCDD > PCB 169 > PCB 105.

Correlations between ∑PCDD/F and ∑PCB as well as between PCDD/F TEQ and PCB TEQ in different study areas are shown in S9 Fig. PCDD/F TEQ and PCB TEQ showed a strong correlation in Finnish Archipelago Sea (R^2^ = 0.97), somewhat less in Northern Quark (R^2^ = 0.86) and in Lake Vanajanselkä (R^2^ = 0.69), but poor in Pristine SW Lake Area (R^2^ = 0.55).

### PBBs

**Fig 9:**
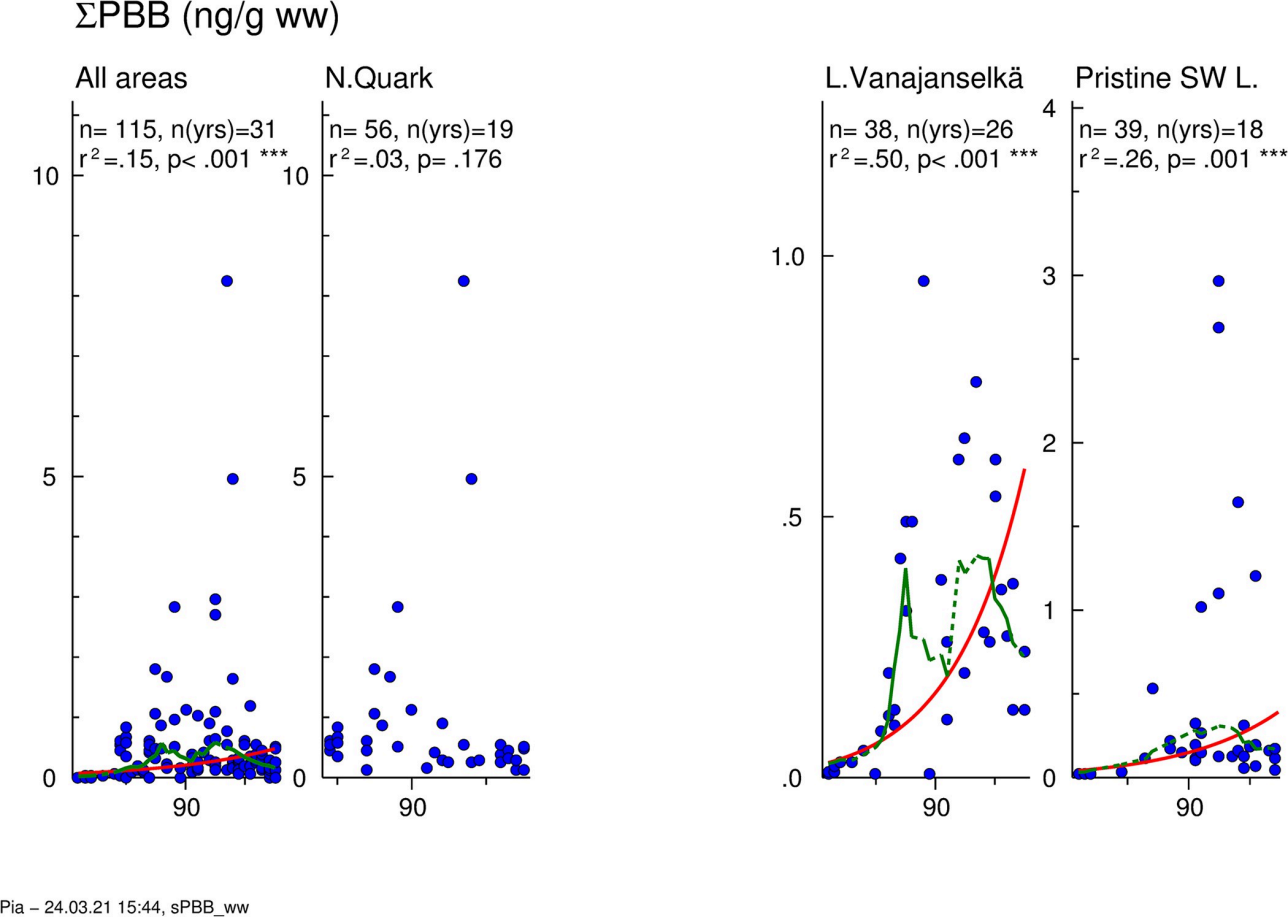
Temporal trend of ∑PBB levels in osprey eggs in the whole dataset and in different study areas. Blue circles indicate individual data, and a red regression line is shown if p<0.05 (two-sided regression analysis). A smoothed green line is shown for non-linear trend components if p < 0.05 (ANOVA). Regression analysis showed significantly increased ∑PBB concentrations in the whole dataset (annual increase 5.6%), in Lake Vanajanselkä (8.7%) and in Pristine SW Lake Area (6.5%), but the smoother explained significantly more of the variation in concentration over time showing a decrease in ∑PBB levels towards the end of the observation period.

**Fig 10:**
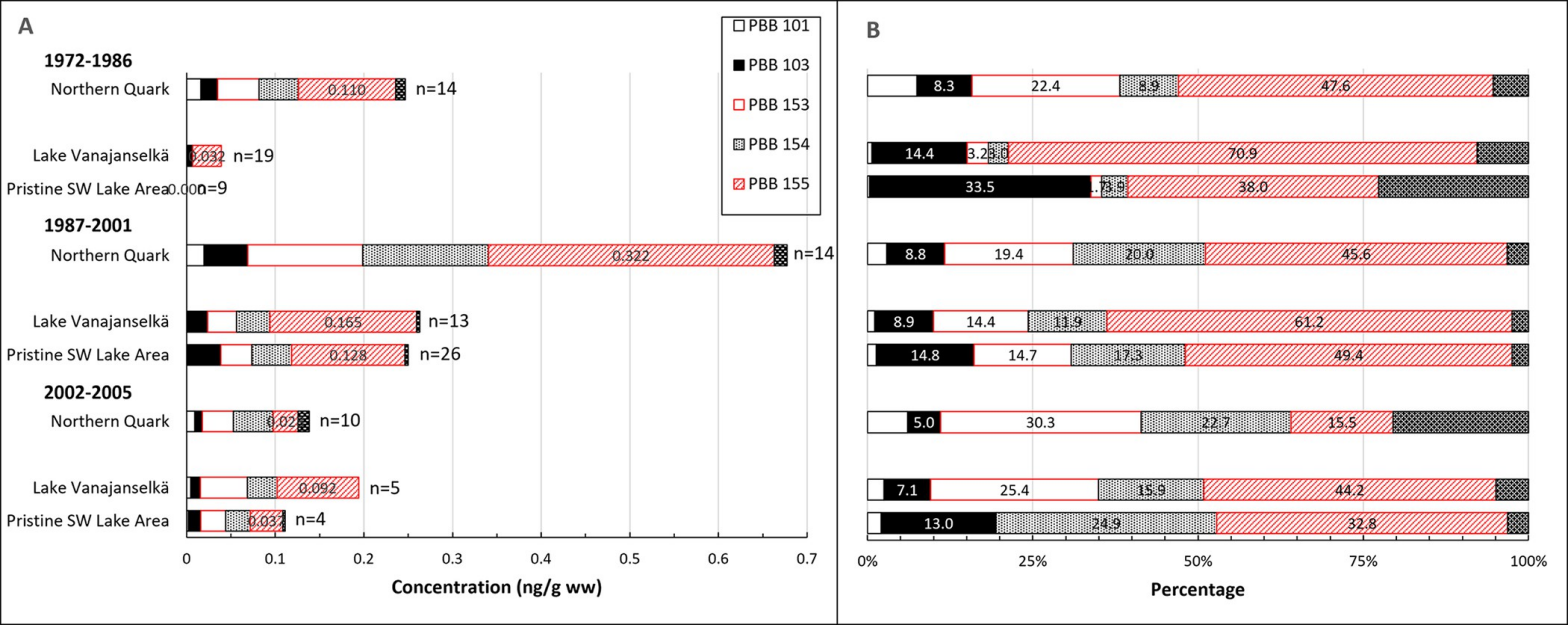
Geometric means of absolute concentrations (A) and average relative (B) concentrations of 5 most abundant PBB congeners in different study areas during the first (1972–1986), second (1987–2001) and third (2002–2005) trimester of the study. The highest ∑PBB concentrations were in Northern Quark during the first and second trimester and in Lake Vanajanselkä during the third trimester. PBB 155 was the most abundant congener except PBB 153 in Northern Quark during the third trimester.

Temporal trends of ∑PBB are presented in Fig 9. GMs of absolute concentrations and average relative concentrations of the 5 most abundant PBB congeners and the sum of other congeners in different study areas are shown in Fig 10. Individual data on ∑PBB of all samples from different years and study areas are shown in S10 Fig.

The highest GMs were in Northern Quark and order study areas of all samples (1972–2005) was Northern Quark 0.428 ng/g ww > Lake Vanajanselkä 0.111 ng/g ww > Pristine SW Lake Area 0.090 ng/g ww (Fig 10 and S10 Fig). Log-linear regression analysis indicated significantly increased ∑PBB in the whole dataset (annual increase 5.6%), in Lake Vanajanselkä and in Pristine SW Lake Area ignoring the decrease after late 1990s (Fig 9, Table 2). The smoother describing the initial increase in ∑PBB followed by the decrease towards the end of the study period was significant for these areas. Also, the highest individual value analyzed since 2002 was only 0.531 ng/g ww (Table 3, S10 Fig).

PBB 155 was the most abundant congener in all study areas (except PBB 153 in Northern Quark during the third trimester), and the other abundant congeners were PBBs 103, 153 and 154. None of them is a dioxin-like congener.

### BDEs

**Fig 11:**
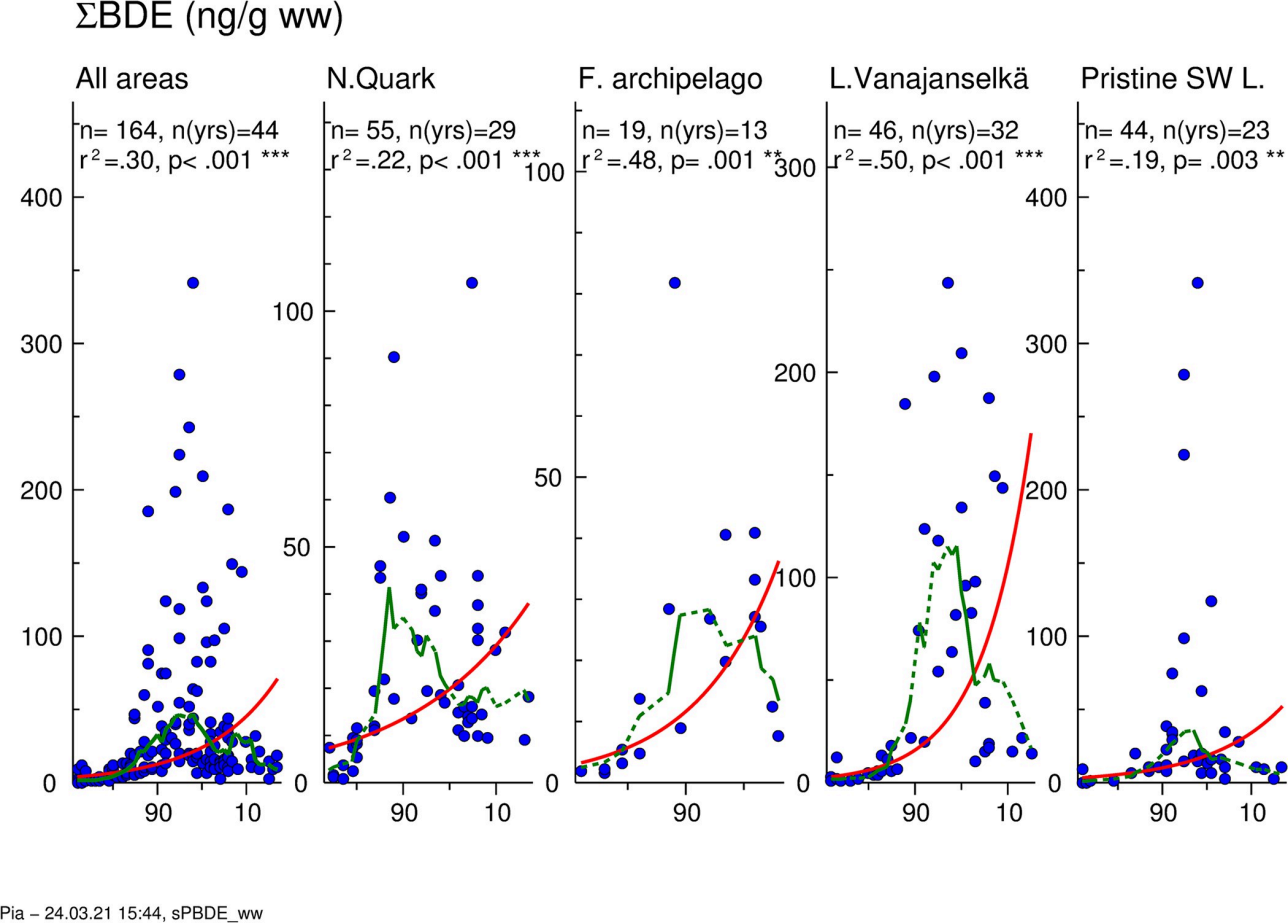
Temporal trend of ∑BDE levels in osprey eggs in different study areas. Blue circles indicate individual data, and a red regression line is shown if p<0.05 (two-sided regression analysis). A smoothed green line is shown for non-linear trend components if p < 0.05 (ANOVA). Regression analysis showed significantly increased ∑BDE concentrations in the whole dataset (annual increase 6.6%) and in all study areas (annual increase 3.9–10.0%). However, the smoother explained significantly more of the variation in concentration over time showing a decrease in ∑BDE levels towards the end of the study period.

**Fig 12:**
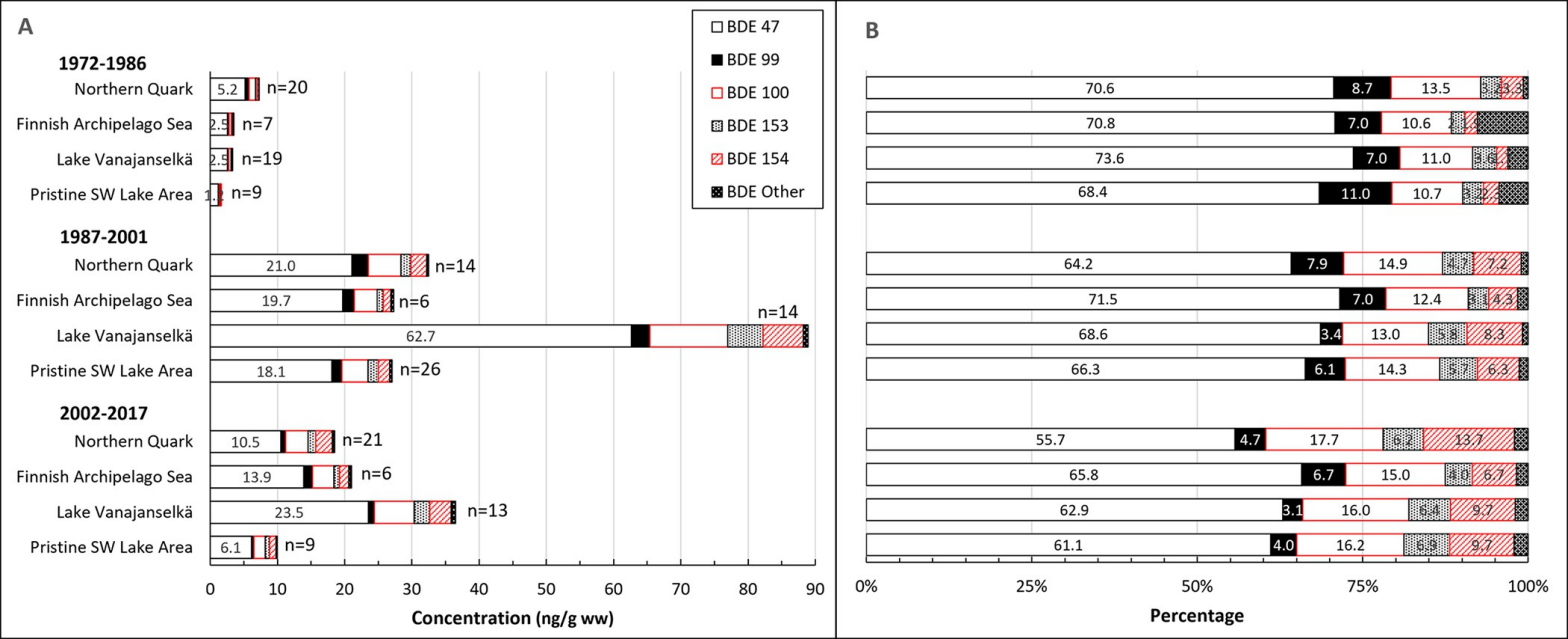
Geometric means of absolute concentrations (A) and average relative (B) concentrations of 5 most abundant BDE congeners and the sum of other congeners in different study areas during the first (1972–1986), second (1987–2001) and third (2002–2017) trimester of the study. Congener profile was quite similar in all areas. Proportion of tetra brominated BDEs 47, 66 and penta brominated BDE 99 decreased and that of penta brominated BDE 100 and hexa brominated BDEs 153 and 154 increased.

Temporal trends of ∑BDE are presented in Fig 11. GMs of absolute concentrations and average relative concentrations of the 5 most abundant BDE congeners and the sum of other congeners in different study areas are shown in Fig 12. Individual data on ∑BDE of all samples from different years and study areas are shown in S11 Fig.

The GM of ∑BDE of all samples (1972–2017) was 15.0 ng/g ww. The levels were highest during the second trimester, and the order study areas was Lake Vanajanselkä 92.0 ng/ ww > Northern Quark 32.9 ng/g ww > Finnish Archipelago Sea 27.5 ng/g ww > Pristine SW Lake Area 27.4 ng/g ww. Log-linear regression analysis indicated significantly increased ∑BDE in the whole dataset (annual increase 6.6%) and in all study areas, which ignores the decrease after late 1990s (Fig 11, Table 2). The smoother describing an initial increase in ∑BDE followed by a decrease towards the end of the study period was significant for the whole dataset and all areas. Peak ∑BDE concentrations were about in 1995, but in Northern Quark slightly earlier. After 2009 the highest analyzed individual value was only 31.8 ng/g ww (S11 Fig).

BDE congener profiles were quite similar in different study areas, and BDE 47 was the most abundant congener followed by BDE 100 (Fig 12). Other abundant congeners were BDEs 99, 153 and 154. The proportion of tetra brominated BDEs 47, 66 and penta brominated BDE 99 decreased over time and that of penta brominated BDE 100 and hexa brominated BDEs 153 and 154 increased.

### PCNs

**Fig 13:**
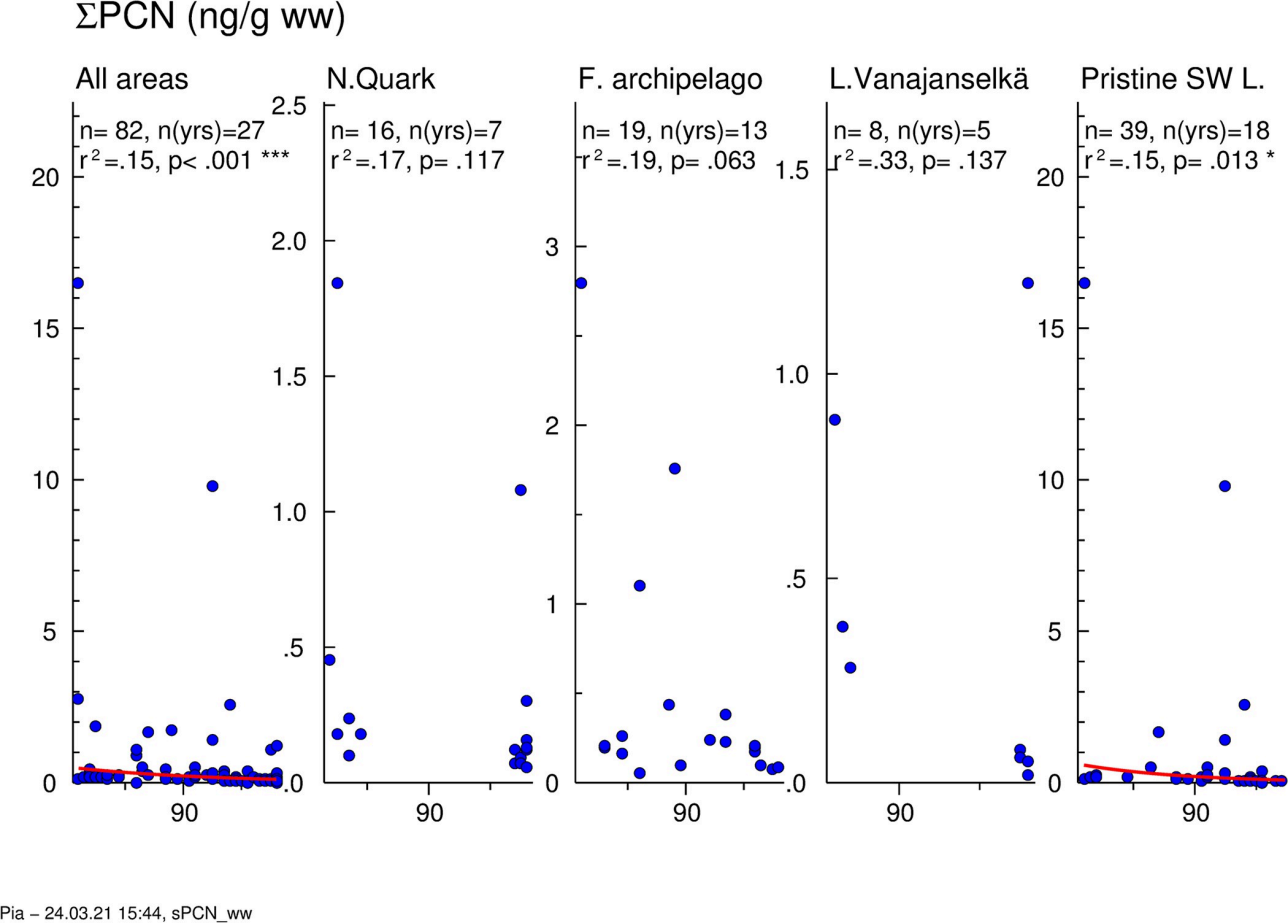
Temporal trend of ∑PCN levels in osprey eggs in different study areas. Blue circles indicate individual data, and a red regression line is shown if p<0.05 (two-sided regression analysis). ∑PCN concentrations decreased significantly in the whole dataset (annual decrease 4.0%) and in all study areas except Northern Quark (annual decrease 2.6–7.0%).

**Fig 14:**
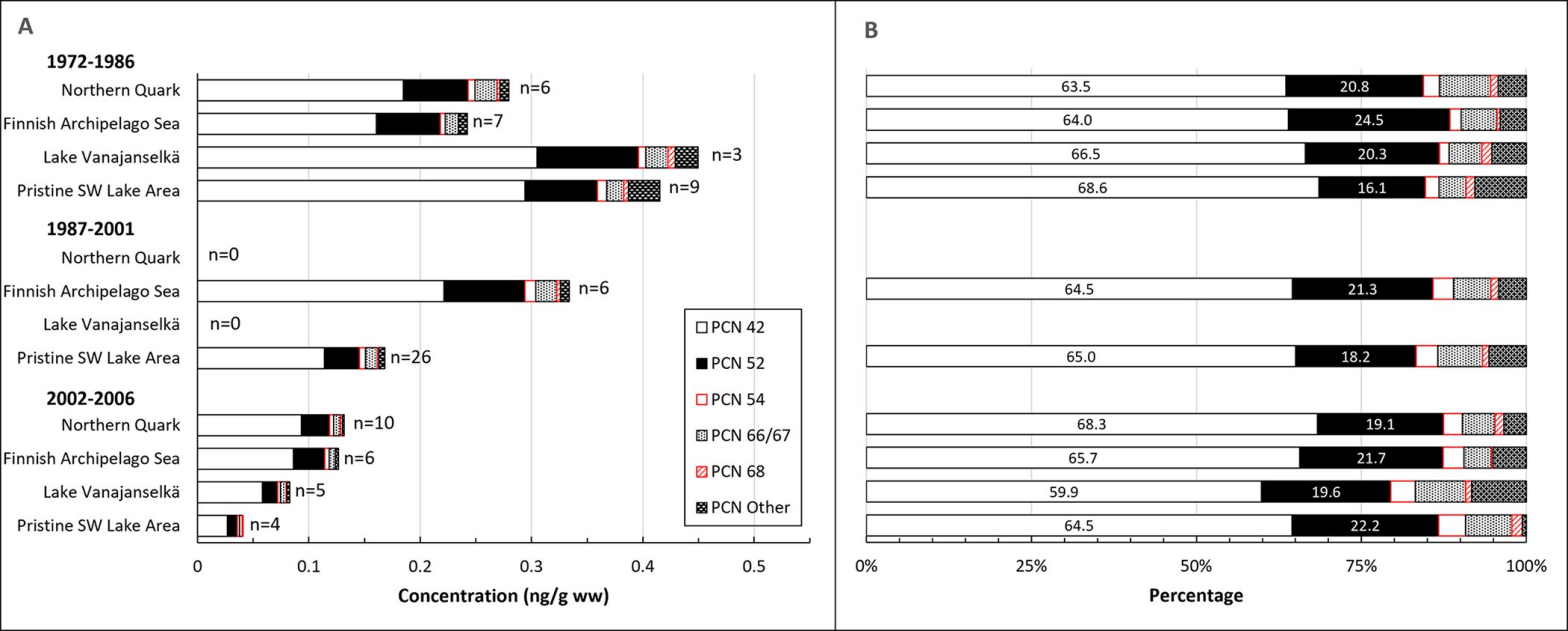
Geometric means of absolute concentrations (A) and average relative (B) concentrations of 5 most abundant PCN congeners and the sum of other congeners in different study areas during the first (1972–1986), second (1987–2001) and third (2002–2006) trimester of the study. PCN 42 was the dominant congener in all study areas followed by PCNs 52 and 66/67. There were no major changes in congener profile over time.

Temporal trends of ∑PCN are presented in Fig 13. Geometric means of absolute concentrations and average relative concentrations of the 5 most abundant PCN congeners and the sum of other congeners in different study areas are shown in Fig 14. Individual data on ∑PCN of all samples from different years (1972–2006) and study areas are shown in S12 Fig.

∑PCN levels of all samples (1972–2006) were quite similar in all study areas (Fig 14 and S12 Fig), and GMs were 0.168–0.226 ng/g ww. The highest GMs were during the first trimester, and the order was Lake Vanajanselkä 0.459 ng/g ww > Pristine SW Lake Area 0.432 ng/g ww > Northern Quark 0.294 ng/g ww > Finnish Archipelago Sea 0.250 ng/g ww. ∑PCN decreased significantly in the whole dataset (annual decrease 4.0%) and in Pristine SW Lake Area (annual decrease 5.7%) (Fig 13, Table 2).

PCN congener profiles did not show major differences among study areas and over time (Fig 14). PCN 42 was the dominant congener in all study areas (on average 60–69%) followed by PCN 52 > PCN 66/67.

### DDTs

**Fig 15:**
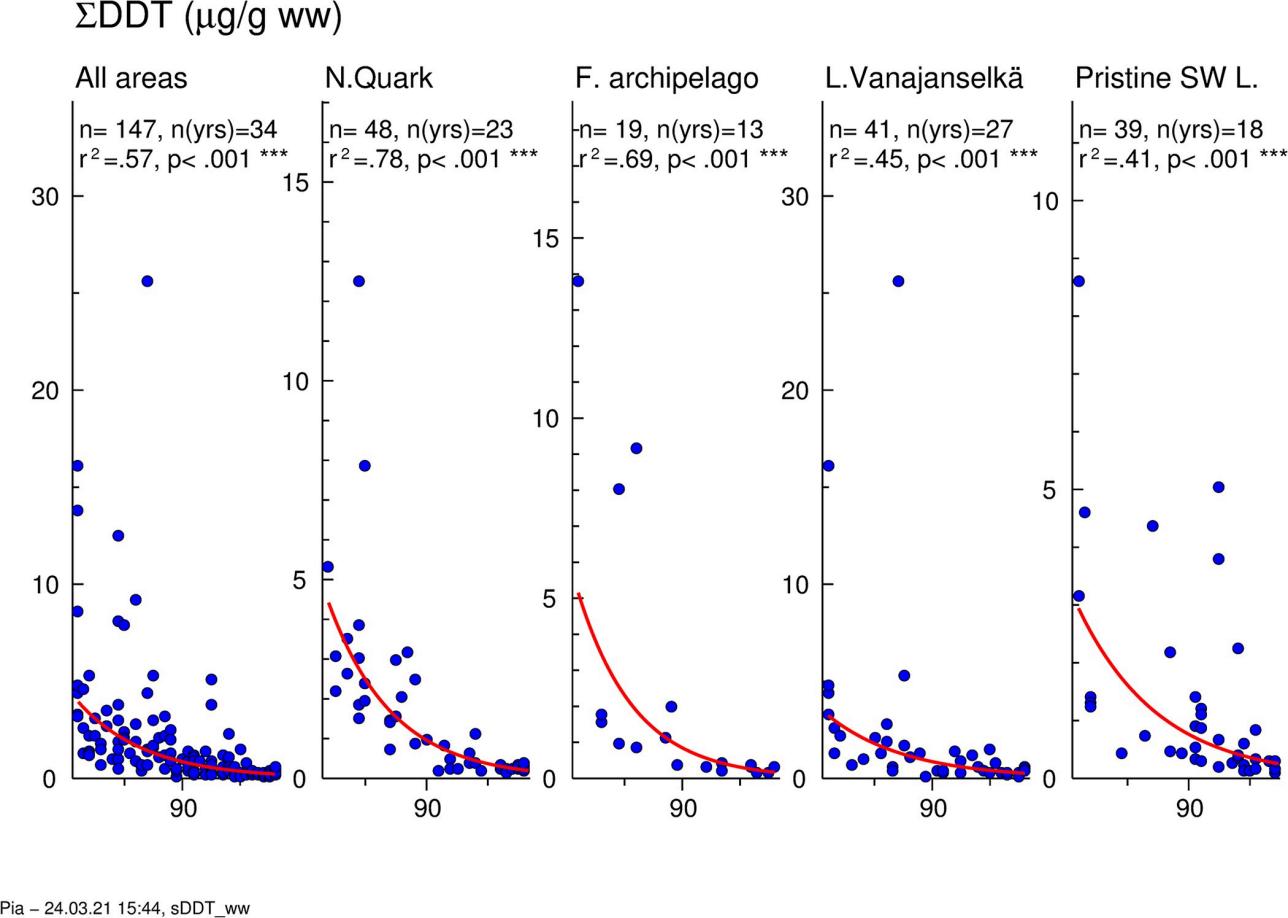
Temporal trend of ∑DDT levels in osprey eggs in different study areas and all areas combined (lower graph). Blue circles indicate individual data, and a red regression line is shown if p<0.05 (two-sided regression analysis). ∑DDT concentrations decreased significantly in the whole dataset (annual decrease 8.1%) and in all study areas (Fig 15, Table 2). The fastest decrease was in Finnish Archipelago Sea (9.5%) and the slowest in Lake Vanajanselkä (7.1%).

**Fig 16:**
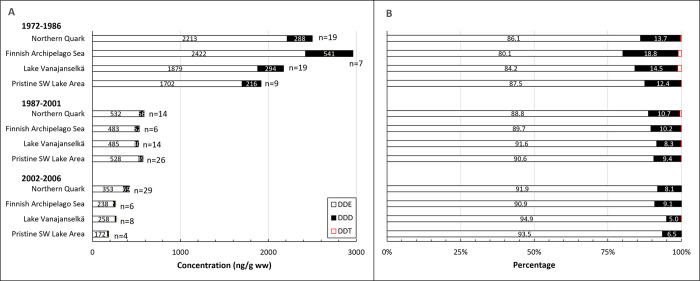
Geometric means of absolute concentrations (A) and average relative (B) concentrations of DDT metabolites DDE, DDD and the parent compound DDT in different study areas during the first (1972–1986), second (1987–2001) and third (2002–2006) trimester of the study. Congener profile was very similar in all study areas demonstrating universal distribution of DDT and its metabolites. The proportion of DDE increased and that of DDD and DDT decreased over time.

Temporal trends of ∑DDT (p,p’ and o,p’ isomers combined) are presented in Fig 15. GMs of absolute concentrations and average relative concentrations of DDT and its main metabolites DDE and DDD in different study areas are shown in Fig 16. Individual data on ∑DDT of all samples from different years (1972–2006) and study areas are shown in S13 Fig.

∑DDT concentrations were quite similar in all study areas (Fig 16 and S13 Fig). The order of GMs of all samples (1972–2006) in different study areas was Lake Vanajanselkä 885 ng/g ww > Northern Quark 800 ng/g ww > Finnish Archipelago Sea 797 ng/g ww > Pristine SW Lake Area 677 ng/g ww. There were high individual values in all study areas during the first trimester, but they disappeared later. ∑DDT concentrations decreased significantly in the whole dataset (annual decrease 8.1%) and in all study areas (Fig 15, Table 2). The fastest decrease was in Finnish Archipelago Sea (9.5%) and the slowest in Lake Vanajanselkä (7.1%).

The congener profile was very similar in all study areas demonstrating universal distribution of DDT and its metabolites (Fig 16). The profile was dominated by the most persistent main metabolite p,p’-DDE, which contributed 86.5–90.2% of ∑DDT in all study areas. The proportion of p,p’-DDD was 9.7–13.0% and that of p,p’-DDT 0.05–0.44%. The proportion of DDE increased from 84.9% (first trimester) to 92.7% (third trimester) and those of DDD and DDT decreased from 14.4% to 7.3% and 0.69 to 0.03%, respectively. DDT was quantified in only a few samples collected during the last two trimesters.

### Spatial differences in prey fish

Analysis of identifiable prey fish present in osprey nests indicated that the main prey was the pike (*Esox lucius*, 40.8%) in Norther Quark and the bream (*Abramis brama*, 50.4%) in the wider area of Lake Vanajanselkä (S1 Table) (56.7% in the actual Lake Vanajanselkä). The proportion of pike in Lake Vanajanselkä area was 6.4% and that of bream in Northern Quark 2.0%. The proportion of predatory fish of total prey in Northern Quark and Lake Vanajanselkä area was 76.1 and 19.3%, respectively, and that of Cyprinid fish 20.4 and 79.3%, respectively. These differences are statistically significant (p<0.001). No prey data are available from Finnish Archipelago Sea, but it is likely that it resembles Northern Quark. Thus, the majority of osprey prey in the Baltic Sea areas is at a higher trophic level than in Lake Vanajanselkä.

### Breeding success

**Fig 17:**
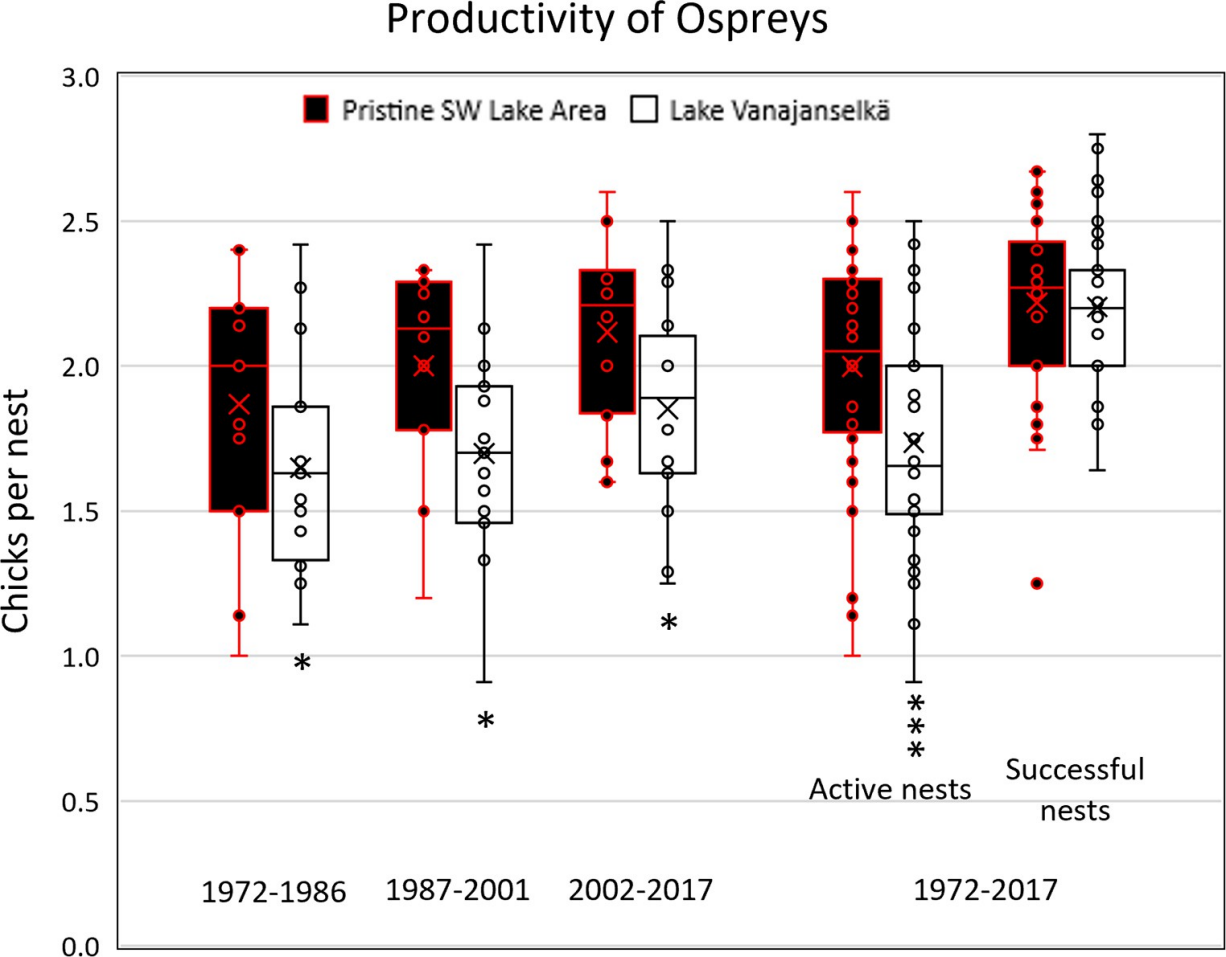
Comparison of average production of chicks per active nest between Pristine SW Lake Area and Lake Vanajanselkä during the first (1972–1986), second (1987–2001) and third (2002–2006) trimester of the study (means (x), medians (horizontal line), interquartile ranges, min and max datapoints excluding outliers and individual values). The four bars on the right show the production during the whole study period per active nest and per successful nest. The average production was significantly decreased when expressed per active nest (nests with laid eggs), but not per successful nest (nests with large chicks). Statistics: * p<0.05, *** p<0.001, Paired T-test.

Comparison of breeding success between Lake Vanajanselkä area and Pristine SW Lake Area over the whole study period indicated that a significantly higher proportion of active nests did not produce large chicks in the former area (19.9%; p<0.001, Paired t-test) than in the latter area (8.5%). Similarly, the annual average production of chicks per active nest was significantly lower (p<0.001) in Lake Vanajanselkä than in Pristine SW Lake Area over the whole study period as well as during all trimesters (Fig 17 and S14 Fig). It is remarkable that the productivity had not completely recovered in Lake Vanajavesi during the study period, although according to the outcome of the nationwide monitoring program Project Pandion productivity of Finnish ospreys has significantly improved during the monitoring period 1971–2022 (S15 Fig). The difference between these two areas was not significant when productivity is expressed per successful nest.

### Migration to wintering areas

The follow-up of ring encounters of ospreys ringed in the study areas showed that the main wintering areas are in West Africa, in areas between Senegal and Cameron (S16 Fig). Locations of ring encounters of ospreys ringed in areas of Northern Quark and Finnish Archipelago Sea did not significantly differ from ospreys ringed in areas of Lake Vanajanselkä and Pristine SW Lake Area.

## Discussion

In this study we analyzed temporal trends and spatial differences of persistent halogenated aromatic hydrocarbons in Baltic and inland water ecosystems utilizing the museum collection of unhatched osprey eggs collected by bird ringers since 1972. The highest sum concentrations of PCDD/Fs, PCBs, TEQs, BDEs and PCNs were in Lake Vanajanselkä, which has received large amounts of industrial effluents from pulp and paper mills etc. and municipal sewage since 1870s [57] and considering the volume of the water body has been affected by far more than the other areas. The second highest concentrations were in Northern Quark. The highest ∑PBB concentrations were in Northern Quark, but ∑DDT levels were almost similar in all study areas. There were noticeable differences among study areas in PCDD/F congener profiles, even between closely located urban Lake Vanajanselkä and Pristine SW Lake Area, which is in agreement with earlier reports on crucial contribution of the nesting area [27, 28]. Profiles of other congener groups were rather similar. In agreement with other studies, statistically significant decreasing trends (annual decrease 3.5–8.1%) were observed over the study period for all other POP groups except the brominated aromatic hydrocarbons BDEs and PBBs. These compound groups showed an increasing trend up to the late 1990s followed by a rapid decline.

### Spatial differences

Concentration of POPs in prey fish in the nesting area is the main determinant of their levels in osprey eggs. Bream and other Cyprinid fish are the main prey of ospreys in Lake Vanajanselkä while pike and other predatory fish are prevalent in Northern Quark (S1 Table). No data are available on POP levels in Lake Vanajanselkä fish, but in Bothnian Sea and Finnish Archipelago Sea PCDD/F TEQs, PCB TEQs and ∑BDEs were much higher in bream than in the predatory fish pike, pike-perch (*Sander lucioperca*) or perch (*Perca fluviatilis*) in 2001–2003 [58]. Also in the National database for fish contaminants maintained by the Finnish Institute for Health and Welfare [59] and the related research reports [60–62] average concentrations in bream were higher than in pike in 2002–2017: 4.23 and 2.64 pg/g ww for ∑PCDD/F, 68.0 and 14.7 ng/g ww for ∑PCB, and 1.39 and 0.54 ng/g ww for ∑BDE (2002), respectively. Similarly in lakes, e.g. in Lake Päijänne (about 80 km east from Lake Vanajanselkä) surrounded by densely populated areas, levels of these compound groups were clearly higher in bream than in pike: 9.99 and 3.23 pg/g ww for ∑PCDD/F, 29.2 and 9.07 ng/g ww for ∑PCB, and 2.24 and 1.78 ng/g ww for ∑BDE (2002), respectively. Therefore, the higher POP concentrations in osprey eggs in Lake Vanajanselkä compared to the other study areas are explained by (1) higher industrial emissions since early 1950’s [57], (2) dominance of bream as a prey, and (3) higher POP concentrations in bream than in pike and other predatory fish. The probable reason for higher PCDD/F, PCB and BDE concentrations in bream than in pike and perch (although they are at a higher trophic level), is the higher fat content of bream. In the fish contaminant reports the average fat concentration of muscle is 3.37% for bream, 0.65% for pike and 1.57% for perch [60–62]. In this database the muscle samples are from skinned fish, and skin removal decreases the fat content. Therefore the POP levels in intact fish are likely to be about 20% higher than in these skinned samples [58].

### PCDD/Fs

∑PCDD/F and PCDD/F TEQ concentrations of all samples decreased significantly on average 3.6 and 4.0% per year, respectively, between 1992–2017 (Table 2). The annual decrease in Northern Quark (4.9%) and in Finnish Archipelago Sea (3.7%) was quite similar to that reported for PCDD/F TEQ in guillemot eggs from Stora Karlsö, southern Baltic Proper for 2002–2011 (5.3%) [63] and for 1969–2016 (2.5%) [6]. In eggs of the arctic seabirds thick-billed murre (*Uria lomvia*) and northern fulmar (*Fulmarus glacialis*) from Prince Leopold Island, the Canadian Arctic (1975–2014), the annual decrease of ∑PCDD/F and PCDD/F TEQs was about 2.1% [44].

According to the Database for fish contaminants [59] the average decrease of PCDD/F TEQ in pike and perch, the main prey fish of osprey in Northern Quark, was quite similar to that in osprey eggs, i.e. 3.3 and 6.6%/year in Bothnian Sea (2002–2017), and in Lake Päijänne the average decrease in pike (2002–2016) and bream (2002–2009) was 5.1 and 8.2%/year, respectively. In Baltic herring from the Finnish Baltic coast (including Northern Quark and Archipelago Sea) the annual decrease of PCDD/F TEQ was 2.8% between 1978–2009 [5].

Availability of appropriate data on trends and levels of POPs in mothers’ milk allows a comparison between the top predator fish eating species and humans, and to find out if the trend observed in the environment is reflected into humans (the One Health concept). In mothers’ milk from Finland (1987–1994) the annual decrease of ∑PCDD/F and PCDD/F TEQ was 3.9 and 3.4%, respectively, in urban areas and 6.4 and 4.6%, respectively, in rural areas [64]. In mothers’ milk from Sweden (1972–2011) the annual decrease of ∑PCDD/F was 6.1% [65].

∑PCDD/F and ∑TEQ concentrations of all samples and the second trimester samples (showing the highest levels) of this study were much lower than those in unhatched eggs of white-tailed sea eagle from South Bothnian Sea and Baltic Proper (Sweden, 1992–2004), but higher than those from Greenland (2000) [39] (Table 3). Similarly with our both Baltic Sea study areas the congener profile of sea eagle eggs was dominated by 2,3,4,7,8-PeCDF, 1,2,3,7,8-PeCDD, 1,2,3,6,7,8-HxCDD and 2,3,7,8-TCDD. However, the proportion of ∑PCDD of ∑PCDD/F was 55 and 42% in osprey eggs from Northern Quark and Finnish Archipelago Sea, respectively, but only 39, 29 and 31% in sea eagle eggs from South Bothnian Sea, Baltic Proper and Greenland, respectively. Compared to our results, ∑PCDD/F levels were much lower in unhatched osprey eggs from the Mediterranean island Menorca (Spain, 1994–2000) [40] and in thick-billed murre and northern fulmar from Prince Leopold Island (1975–2014) [44], but the dominating congeners were largely the same. In osprey eggs from Menorca the congener profile was quite similar to ours, dominated by 2,3,4,7,8-PeCDF, 1,2,3,6,7,8-HxCDD, 1,2,3,7,8-PeCDD and OCDD, and the proportion of ∑PCDD of ∑PCDD/F was 43.3%, which is similar to our second trimester value in Finnish Archipelago Sea (42.2%), but lower than in the other study areas (54.9–78.9%). In Prince Leopold Island the dominating congeners were also 2,3,4,7,8-PeCDF, 1,2,3,7,8-PeCDD, 1,2,3,6,7,8-HxCDD and 2,3,7,8-TCDD.

∑PCDD/F levels were much higher in osprey eggs from the upper Willamette River (Oregon, USA) collected in 1993 and 2001 [41] than those in the present study (Table 3). However, the congener profile of these eggs was completely different, dominated by the higher chlorinated congeners OCDD, 1,2,3,4,6,7,8-HpCDD, 1,2,3,6,7,8-HxCDD and 1,2,3,4,6,7,8-Hp-CDF. Therefore PCDD/F TEQs were similar to our second trimester PCDD/F TEQs. On the other hand, the proportion of ∑PCDD of ∑PCDD/F was considerably higher (97%) than our second trimester proportion (64.7%) indicating the dominant role of PCDD impurities in local chlorine bleached-kraft mill effluents. Also several other studies from North American rivers reported high PCDD concentrations in osprey eggs from nests downstream of bleached-kraft pulp mills compared to nests upstream [e.g. 26, 27]. In Wisconsin (1992–1996), GMs of PCDD/F TEQs downstream bleached-kraft mill facilities were much higher than in this study, but in the upstream (reference) area similar to Pristine SW Lake Area [43]. Also here the role of PCDDs was much more dominant than in the present study. The proportion of 2,3,7,8-TCDD was 83.1–91.7% of PCDD/F TEQ in the downstream area. Implementation of chlorine free bleaching technology has resulted in significant decreases especially in levels of 2,3,7,8-TCDD, 1,2,3,7,8-PeCDD, 1,2,3,6,7,8-HxCDD, and 2,3,7,8-TCDF [27]. Local use of pentachlorophenol containing wood preservatives was suggested to explain the relatively high concentrations of higher chlorinated congeners, such as HxCDD, HpCDD, OCDD and HpCDF. In most cases even the high concentrations did not seem to affect hatching success or fledging, although lowered growth rate of osprey nestlings was reported in the contaminated area as compared to the reference area [43].

PCDD/F congener profiles showed some notable differences among the study areas. 2,3,4,7,8-PeCDF was the dominant congener in both Baltic Sea areas (30.8–44.4% of ∑PCDD/F) and 1,2,3,6,7,8-HxCDD in Lake Vanajanselkä (24.9–31.3%). 2,3,4,7,8-PeCDF together with 2,3,7,8-TCDF were the dominant congeners in pike, Baltic herring and salmon from the Baltic Sea [5, 59]. However, in pike and bream from lakes 2,3,7,8-TCDF is by far the dominant congener followed by 2,3,4,7,8-PeCDF. In all areas the next common congeners were 1,2,3,7,8-PeCDD and 1,2,3,6,7,8-HxCDD. Thus, osprey eggs reflected the congener profile of prey fish except that 2,3,7,8-TCDF had a lower proportion and 1,2,3,6,7,8-HxCDD a higher proportion than expected. The possible reasons for the dominance of 1,2,3,6,7,8-HxCDD in osprey eggs in Lake Vanajanselkä are local source(s) of emission and/or species-specific accumulation. In Pristine SW Lake Area proportion of the following 3 congeners was quite similar: 2,3,4,7,8-PeCDF (13.9–19.6%), 1,2,3,6,7,8-HxCDD (14.4–17.3%) and 1,2,3,7,8-PeCDD (12.5–17.4%). Overall, it is interesting to note that these three congeners are commonly observed in eggs of fish-eating birds in different areas [39, 44], which reflects similarity in sources of emissions and toxicokinetics of PCDD/Fs. 2,3,4,7,8-PeCDF is formed in a variety of combustion processes [66], it is a major impurity of commercially used technical PCB mixtures [67] and a minor impurity of formerly widely used chlorophenol fungicide KY-5 [68]. Other sources of PCDD/F impurities include chlorine bleaching of pulp and paper (especially in Lake Vanajanselkä), metal industry and chloralkali industry [69]. A statistical modeling approach using the positive matrix factorization has been utilized to identify the main PCDD/F sources to Baltic herring (*Clupea harengus*) [70]. This approach is also useful for assessing potential sources of osprey egg contaminants, because the PCDD/F congener profile of Baltic herring shares several dominating congeners with osprey eggs, including 2,3,4,7,8-PeCDF, 1,2,3,7,8-PeCDD, 2,3,7,8-TCDF, 2,3,7,8-TCDD, 1,2,3,6,7,8-HxCDD and OCDD [5, 71]. Based on PCDD/F concentrations in Baltic herring and surface sediments the study showed that thermal sources (such as waste incineration) via atmospheric deposition are main contributors of TEQ, but their importance is decreasing in line with declining air emissions. At the same time the relative importance of historical chlorophenol-related PCDD/F sources mainly from surface sediments may be increasing.

Comparison between PCDD/F TEQ levels in osprey eggs of the present study and Swedish mothers’ milk samples (1972–2011) [65] indicates that on lipid weight basis the PCDD/F TEQs in osprey eggs (first trimester GM 370 pg/g lipid, third trimester GM 113 pg/g lipid) were about 14–42 times higher than those in mothers’ milk (mean in 1972 26 pg/g lipid, mean in 2011 2.7 pg/g lipid). The proportion of PCDD TEQ in PCDD/F TEQ was higher in osprey eggs (69.7–86.3%) than in mothers’ milk (ca. 55–77%). The dominant PCDD/F congeners in were largely the same as in osprey eggs, i.e. 2,3,7,8-PeCDF, 1,2,3,7,8-PeCDD, 1,2,3,6,7,8-HxCDD, but also OCDD [65].

### PCBs

∑PCB and PCB TEQ concentrations of all samples decreased annually by 3.5 and 4.0%, respectively, between 1992–2017 (Table 2). The annual decrease of ∑PCB and PCB TEQ in Northern Quark (4.2 and 4.4%) and in Finnish Archipelago Sea (3.3%) was slower than in guillemot eggs from Stora Karlsö, southern Baltic Proper for 1969–1995 (8.6%) [22] and for 1969–2016 (8.6%) [6]. In a recent time-trend directed non-target screening study the annual decrease estimated for the dominant PCB congeners 153, 138, 180 and 126 in guillemot eggs from Stora Karlsö (1986–2019) was 7–8%, and for white-tailed sea eagle muscle from the Swedish Baltic Sea coast (1965–2017) 4–5% [72]. Annual decrease of 6.3–7.2% was reported in osprey eggs from Delaware Bay area, New Jersey, USA (1989–1998) [73]. In murre and fulmar eggs from Prince Leopold Island (1975–2014), the annual decrease of non-ortho ∑PCB was only about 1.6 and 2.0%, respectively and that of PCB TEQs was about 2.1% for both species [44].

The average decrease of PCB TEQ in pike and perch in Northern Quark was similar to that in osprey eggs, i.e. 4.2 and 6.3%/year in Bothnian Sea (2002–2017), and in Lake Päijänne the average decrease in pike (2002–2016) and bream (2002–2009) was 5.3 and 9.6%/year, respectively [59]. In Baltic herring from the Finnish Baltic coast (1978–2009) indicator PCBs and PCB TEQs decreased annually by about 2.8% [5]. In mothers’ milk from Finland (1987–1994) the annual decrease of ∑PCB and PCB TEQ was 6.7 and 7.1%, respectively in urban areas and 7,4 and 8.0%, respectively, in rural areas [64], while in mothers’ milk from Sweden (1972–2011) the annual decrease of dioxin-like PCBs was 6.9% [65].

The first trimester ∑PCB concentrations were also more than an order of magnitude lower than those in unhatched white-tailed sea eagle eggs from Swedish Baltic coast (1970–1974, and also 1995–1997) [45] as well as in guillemot eggs from Stora Karlsö, Baltic Proper (1969) [22] (Table 2). ∑PCB levels in second trimester eggs from the most contaminated Lake Vanajanselkä area were quite similar with those in osprey eggs from Norway (1991–1997) [32], Menorca (1994–2000) [40], the Great Lakes Basin (Canada, 1991–1995) [47] and the USA East Coast regions of Chesapeake Bay (2000–2001) [48] and Delaware River and Bay (2002) [49], although some much higher values were analyzed in eggs from the North American regions. On the other hand, second trimester levels in the other study areas were quite similar with those in osprey eggs from Wisconsin [43] and Pacific Northwest [41, 42, 46].

In this study PCB 153 was the dominant NDL-PCB congener in all study areas and over time (18.4–29.5% of ∑PCB) followed by PCB 138 (13.3–16.0%) and PCB 180 (8.9–18.4%). The order of these congeners was the same in pike, bream and Baltic herring from Bothnian Sea, Archipelago Sea and Finnish lakes [5, 60–62]. These congeners were dominant also in sea eagle eggs from Sweden [45], in osprey muscle from Sweden (1982–1986) [74], in osprey eggs from Willamette [75], Fraser and Columbia Rivers [46] in the Pacific Northwest and in osprey eggs from Menorca, where the proportions were PCBs 180 36%, PCB 153 34% and PCB 138 13% [40].

PCB TEQs of all samples in all study areas and the second trimester PCB TEQs in Northern Quark and Finnish Archipelago Sea were also more than an order of magnitude lower than non-ortho PCB TEQs in sea eagle eggs from Sweden (1992–2001) and lower than those from Greenland (2000) [39] (Table 2). On the other hand, levels in our study were similar to PCB TEQs in murre and fulmar eggs from Prince Leopold Island [44]. In our study the dominant non-ortho PCB congener in all study areas was PCB 126 (83.0–87.4% of PCB TEQ), which is quite similar to osprey eggs from Menorca (77.2% of ∑TEQ) [40] as well as to murre (65–75% of non-ortho PCB TEQ) and fulmar (>90% of non-ortho PCB TEQ) eggs from Prince Leopold Island.

PCB TEQs in osprey eggs of this study (first trimester GM 1806 pg/g lipid, third trimester GM 563 pg/g lipid) were on lipid weight basis about 60–296 times higher than those in Swedish mothers’ milk samples (1972–2011) [65] (mean in 1972 30 pg/g lipid, mean in 2011 1.9 pg/g lipid). The proportion of PCB TEQ in ∑TEQ was higher in osprey eggs (81.0–82.6%) than in mothers’ milk (ca. 28–53%). PCB 126 was also the dominant DL PCB congener in mothers’ milk.

Similar to dioxin-like compounds there are remarkable differences in sensitivity to PCBs among avian species, and ospreys seem to be relatively resistant to toxic effects of NDL-PCBs at least at the population level [26]. No adverse reproductive effects have been reported even at the mean PCB concentration of 25000 ng/g ww in osprey eggs [47, 76, 77]. NDL-PCB-related toxic effects are therefore very unlikely in the context of the present study.

### ∑TEQs

∑TEQ of all samples decreased significantly by 4.0% per year between 1992–2017 (Table 2). In murre and fulmar eggs from Prince Leopold Island (1975–2014) the annual decrease of ∑TEQ was again slower, about 2.0% [44]. Similar to PCDD/F TEQ and PCB TEQ the average annual decrease of ∑TEQ in pike and perch in Northern Quark was quite similar to that in osprey eggs, i.e. 3.8 and 6.5% in Bothnian Sea (2002–2017), and in Lake Päijänne the average decrease in pike (2002–2016) and bream (2002–2009) was 5.2 and 6.9%, respectively [59]. In Baltic herring from the Finnish Baltic coast (including Northern Quark and Archipelago Sea) the annual decrease of PCDD/F TEQ was 2.8% between 1978–2009 [5]. Compared to osprey eggs the decrease in Swedish mothers’ milk ∑TEQ (1972–2011) slightly faster 6.5% [65].

∑TEQs of all samples and the second trimester samples of the present study from all areas except Lake Vanajanselkä were much lower than in white-tailed sea eagle eggs from Baltic Sea (Sweden, 1992–2004) [39], but rather similar to osprey eggs from Menorca (1994–2000) [40] and Columbia River (1997–2004) [42] as well as murre and fulmar eggs from Prince Leopold Island (1975–2014) [44] (Table 2). The proportions of PCB TEQ, PCDD TEQ and PCDF TEQ of ∑TEQ in different study areas of this study were 80.0–84.3%, 12.2–16.2% and 2.00–6.51% of ∑TEQ, respectively, and the proportion of PCB TEQ was similar (80.0% of ∑TEQ, non-ortho PCBs only) in osprey eggs from Menorca. In the database for fish contaminants the proportion of PCDD/F TEQ was higher and that of PCB TEQ lower than in our osprey eggs, i.e. in 46.6 and 53.4%the pike and 40.0 and 60.0% in the bream, respectively, in all areas [59]. In white-tailed sea eagle eggs from Baltic Sea and Greenland the proportion of PCB TEQ was higher than in osprey eggs (92.5 and 94.6%, respectively; non-ortho PCBs only) and that of PCDD TEQ lower (2.6 and 1.7%) [39]. The likely explanations are species specific differences in diet or elimination of these compound groups. In murre and fulmar eggs from Prince Leopold Island the proportion of PCB TEQ was lower than in the present study, i.e. 48–69% and 37–48%, respectively, and that of PCDF TEQ were much higher, 20–41% and 42–51%, respectively [44]. Compared to osprey eggs of the present study the proportion of PCB TEQ in Baltic fish and lake fish from Finland was clearly lower, only 49±12% [58].

In this study the dominant PCB and PCDD/F congeners contributing to the ∑TEQ in osprey eggs were PCB 126 (61.8–72.3%), 1,2,3,7,8-PeCDD (6.4–11.2%), PCB 118 (3.1–8.2%) and 2,3,4,7,8-PeCDF. In osprey eggs from Menorca and in sea eagle eggs from Baltic Sea the dominant ∑TEQ congeners were PCB 126, PCB 169 and PCB 77 [39, 40], and in murre eggs from Prince Leopold Island PCB 126, PCB 77 and 2,3,4,7,8-PeCDF [44]. In fulmar eggs from the same island, however, the contribution of PCDFs to ∑TEQ was greater, increased over time, and the dominant congeners were 2,3,4,7,8-PeCDF and PCB 126. In Baltic fish and lake fish from Finland PCB 126 contributed 32% to ∑TEQ [58].

∑TEQs in osprey eggs of this study (first trimester GM 2211 pg/g lipid, third trimester GM 682 pg/g lipid) were about 40–148 times higher than those in Swedish mothers’ milk samples (1972–2011) [65] (mean in 1972 56 pg/g lipid, mean in 2011 4.6 pg/g lipid). In mothers’ milk the proportions of PCB TEQ, PCDD TEQ and PCDF TEQs of ∑TEQ were approximately 50, 30 and 20%, respectively. Thus, the proportion of PCB TEQs was lower and that of PCDD and PCDF TEQs higher than in osprey eggs.

There are up to 1000-fold sensitivity differences in biochemical and toxic effects of dioxin-like compounds among avian species, and this variability is mainly based on the genotype for the ligand-binding domain of AHR1 [78, 79]. Resistant species show lower binding affinity of TCDD to AHR1 and reduced ability for TCDD-dependent transactivation. Osprey is considered relatively insensitive to dioxin-like compounds [79, 80], and field observations together with experimental studies indicated that the no-observed-adverse-effect level (NOAEL) for embryo survival (hatching) was 136 pg total ∑TEQ/g ww in osprey eggs [43, 77]. Decreased embryo survival is considered the most sensitive toxic effects of dioxin-like compounds in birds, but induction of the xenobiotic metabolizing cytochrome P450 (CYP) 1A enzyme in the liver is an adaptive response and a sensitive indicator of exposure to dioxin-like compounds. The no-observed effect level (NOEL) for CYP 1A induction in osprey chicks is 37 pg TEQ/g ww in the egg and the lowest-observed effect level (LOEL) 130 pg TEQ/g ww [77]. Based on these data it is plausible that maternal exposure to dioxin-like compounds has resulted in decreased embryonic survival among highly exposed ospreys especially in Lake Vanajanselkä and Northern Quark, where ∑TEQ levels in eggs frequently exceeded the NOAEL of 136 pg ∑TEQ/g ww prior to 2010 (S7 Fig). This is in agreement with the significantly decreased productivity per active nest in Lake Vanajanselkä. Furthermore, because the productivity per nests with large chicks was not decreased, the likely explanation is decreased number of chicks due to increased embryonic mortality.

### PBBs

In this study concentrations of ∑PBB and abundant congeners increased from early 1970s to late 1990s after which they slowly decreased. Only a few previous studies on temporal trends on PBBs are available (none of them in raptors), and in accordance with our findings they indicate a slow decrease. PBB 153 concentrations did not significantly change in lake trout of the Great Lakes over the period 1980–2000 [81]. The only exception was Lake Huron, where they decreased with a half-life of 19±5 years. A significant decrease in PBB 153 with a population half-life of about 12 years was also found in 40 human serum pools collected in Southeast USA in 1985–2002 [82].

Overall, PBB concentrations were very low in the present study compared to those reported in samples from other areas. Commercial production of PBB-based flame retardants started in early 1970s, although they were not produced or used in Finland. This is the likely explanation for low concentrations in osprey eggs. ∑PBB concentrations of all samples (1972–2005) were lower than those in eggs of predatory birds collected in Norway during 1991–2002 [50, 51] (Table 3A). However, ∑PBB concentrations in osprey eggs collected during peak levels (1987–2001) from Northern Quark were similar to those in unhatched osprey eggs from Southern Norway, i.e. about 1 ng/g ww [50], but concentrations in all other areas and trimesters were much lower (GMs ND-0.26). The highest median ∑PBB concentrations in Norway were in peregrine falcon (*Falco peregrinus*) and in white-tailed sea eagle, 19 and 15 ng/g ww, respectively, while in eggs of goshawk (*Accipiter gentilis*), golden eagle (*Aquila chrysaetos*) and merlin (*Falco columbarius*) they were about 1–2 ng/g ww. For comparison to other POP groups, GM of ∑PBB of all study areas was 8500 times lower than that of ∑PCB and 93 times lower than that of ∑BDE. Proportions of ∑PBB were slightly higher in sea eagle eggs from Norway [83].

Hexabrominated PBB 155 was the most abundant congener in all study areas of this study, and the other abundant congeners were PBB 154 and 153 as well as pentabrominated PBB 103 and 101. In eggs of 6 predatory bird species from Norway PBBs 101 and 153 were found in all of them, and in all species the dominating congeners were PBBs 153, 154 and 155, of which PBB 153 was the most abundant in all other species except peregrine falcon, in which PBB 155 was the most abundant [50, 51]. PBB 153 is the main constituent of FireMaster® products, but PBB 154 and 155 are formed by photochemical debromination of decabromobiphenyl that was also commercially produced [13]. This is the plausible explanation for the dominance of PBB 155 in Finland. It is also interesting to note that in human milk and placental samples collected from Denmark and Finland in 1997–2001 PBBs 153 and 155 were the most abundant PBB congeners, and PBB 153 was significantly higher in Demark and PBB 155 significantly higher in Finland [84].

In our study ∑PBB and PBB 153 GMs and means were higher in Northern Quark than in inland lake areas. Accordingly, analysis of PBB 153 in 44 unhatched white-tailed sea eagle eggs collected from different regions of Sweden in 1992–2005 indicated higher concentrations in the Baltic regions than in inland regions [52]. The GMs were 1.0, 1.5, 5.0 and 6.0 ng/g ww for inland north Lapland, inland lakes in central and southern Sweden, South Bothnian Sea and Baltic Proper, respectively. In the present study PBB 153 GMs during second trimester were much lower, i.e. 0.13, 0.033 and 0.035 ng/g ww for Northern Quark, Lake Vanajanselkä and Pristine SW Lake Area, respectively. There is no specific explanation for this Baltic–inland lake difference except higher concentrations of these compounds in generally more polluted Baltic Sea [52].

### BDEs

∑_11_BDE levels increased rapidly in mid-1980s, peaked in about 1995 and declined rapidly after 2005–2010. A very similar trend for dominant BDE congeners 47, 99 and 100 was described for guillemot eggs from Stora Karlsö (1969–2016) with the peak a few years earlier, in early 1990s [6]. Also the ∑_14_BDE trend in Baltic herring from the Baltic coast of Finland was similar [5]. In pike and perch from Northern Quark the decrease of ∑BDE (without BDE 209) was 5.1 and 6.5%/year in Bothnian Sea (2002–2017), and in Lake Päijänne the average decrease in pike (2002–2016) and bream (2002–2009) was 5.1 and 5.8%/year, respectively [59]. For comparison, in Baltic herring from the Finnish Baltic coast (1978–2009) indicator PCBs and PCB TEQs decreased annually by about 2.8% [5].

In this study the highest levels of ∑_11_BDE were in Lake Vanajanselkä during the second trimester, and they were quite similar with ∑_9_BDE in unhatched osprey eggs from southern Norway [50] (Table 3B). However, ∑_5_BDE levels were higher in unhatched sea eagle eggs from the Swedish Baltic areas South Bothnian Sea and Baltic Proper, but lower in inland north Lapland and central and southern inland lakes [52]. Overall, BDE concentrations were higher in osprey eggs in North America than in Europe. In eggs from the East Coast, regions of Chesapeake Bay (2000–2001) [48] and Delaware River and Bay (USA, 2002) [49] the levels were quite similar with those in Columbia, Willamette and Yakima Rivers in Oregon and Washington (USA, 1991–2000) [54].

In the present study the BDE congener profile was quite constant in different study areas and over time following the order BDE 47 > 100 ≥ 99 >154 >153. The 5 most abundant congeners were mainly the same and the order abundance was similar both in pike and bream from Bothnian Sea, Archipelago Sea and Lake Päijänne [60–62] as well as in osprey eggs from different areas of Europe and North America with some variability only between the order of BDEs 99 and 100 as well as BDEs 153 and 154. Congener profile was very similar in unhatched osprey eggs from southern Norway [50], Sweden [53], USA East Coast [48, 49], USA and Canada Pacific North West [54, 85] as well as in sea eagle eggs from Sweden [52]. In these two species the proportion of BDE 47 was typically >50% of ∑BDE. The congener profile was largely similar in Baltic and fresh water fish species in Finland [86], and also in eggs of other piscivore avian species living in the same areas with ospreys, such as guillemot [87], great blue heron (*Ardea herodias*) and double-crested cormorant (*Phalacrocorax auritus*) [85], but different, dominated by BDE 153, in raptor species relying on terrestrial food chains, such as peregrine falcon [88], merlin and golden eagle [50]. This dissimilarity is most likely due to the difference in prey congener profile between aquatic and terrestrial habitats.

Overall, the expected source of these dominant congeners in osprey eggs is the formerly used technical PeBDE mixtures, e.g. t DE-71 and Bromkal 70-5DE the ingredients of which are BDE 99 > 47 > 100 > 153 > 154 [89]. The sale of commercial PeBDE and octaBDE mixtures was banned in EU in 2004, but it appears that the use had partly been voluntarily phased out even earlier. Technical decaBDE (BDE 209) has still been used later for some applications, but the use is now strictly limited and regulated. BDE 209 was originally considered almost inert due to very low bioavailability but was later shown to undergo both abiotic and biotic debromination leading to formation of more toxic and persistent hepta to nonabrominated BDEs. Debromination of BDE 209 has been shown in xenobiotic metabolism of several avian species including European starling (*Sturnus vulgaris*), herring gull (*Larus argentatus*) and American kestrel (*Falco sparverius*) [90]. Due to low levels of hepta to nonaBDEs in osprey eggs of the present study it seems unlikely that debromination products of BDE 209 would have an important contribution to BDE exposure or congener profile.

Human exposure to BDE congeners seems to be qualitatively quite similar with that of ospreys. In mothers’ milk from Finland and Denmark (1997–2001) the dominant congeners were BDE 47 > 153 > 99 > 100 [91]. The median ∑_14_BDE concentration in mothers’ milk (0.12 ng/g ww) was 316 times lower than our second trimester GM for ∑_11_BDE (37.9 ng/g ww). European dietary survey indicated that the dominating congeners for dietary exposure are BDE 209 (not analyzed in the present study) followed by BDE 47 and 100 [89].

Field studies in ospreys indicated that ∑BDE levels >1000 ng/g ww in eggs are linked to significantly reduced reproductive performance (productivity) by nearly 50%, but no adverse effects were observed at levels <1000 ng/g ww [54]. It is therefore very unlikely that the ∑BDE levels observed in this study would have had adverse effects on reproduction.

### PCNs

∑PCN concentrations of all samples decreased significantly on average 4.0% per year between 1972–2006 (Table 2). In quite good agreement with our data annual decrease of 5.6% was reported in guillemot eggs from Stora Karlsö (1976–1987) [55] and annual decrease of 4.5% in peregrine falcon eggs from South-Greenland (1986–2014) [92]. The decrease was slower (2.8%) in thick-billed murre eggs from Prince Leopold Island (1975–2014) [56].

∑PCN concentrations of all study areas during the highest concentrations, i.e. the first and second trimesters, were much lower than those reported in white tailed sea eagle eggs from Baltic Proper (1985–1989) and guillemot egg from Gotland (1974–1987) [55] (Table 3C). They were also lower than those in thick-billed murre from Prince Leopold Island in the Canadian Arctic (1975–1998), but during the third trimester the levels were similar or even slightly higher than in the Canadian Arctic (2003–2006) [56].

In this study PCN 42 was the dominating congener followed by PCN 52 and PCN 66/67 in all study areas and over time. A common feature of these congeners is that they are bioaccumulative, because they lack vicinal unsubstituted carbons, which can be hydroxylated via arene oxide intermediates [56]. Tetra- and pentachlorinated PCNs (such as PCNs 42 and 52) dominate in combustion fly ash whereas penta- and hexachlorinated PCN are impurities in commercial PCB mixtures. In 35 osprey muscle samples from Sweden (1982–86) the PCN profile was dominated by pentachlorinated PCNs and hexachlorinated PCNs 66/67 [74]. In a white-tailed sea eagle egg from Baltic Proper (1989) PCN 60 dominated followed by PCNs 52, 42 and 66/67, and in guillemot eggs from Gotland (1987) PCN 66/67 dominated followed by PCNs 60, 73, 52 and 42) [55]. Similarly with our data PCNs 42, 52/60 and 66/67 were also among the predominant congeners in thick-billed murre eggs from Canadian Arctic [56] and in peregrine falcon eggs from South-Greenland [92].

### DDTs

∑DDT concentrations of all samples decreased significantly and faster than sum concentrations of other compound groups, on average 8.1% per year between 1992–2006 (Table 2). The annual decrease in Northern Quark (9.1%) and in Finnish Archipelago Sea (9.5%) was quite similar to that reported for DDE in guillemot eggs from Stora Karlsö for 1969–2016 (8.9%) [6]. Slightly slower annual decreases of 5.0–7.0% were reported in osprey eggs from Delaware Bay area (1989–1998) [73].

∑DDT and *p*,*p’*-DDE concentrations were very similar in all study areas indicating lack of local sources of exposure. *p*,*p’*-DDE concentrations were similar with those in unhatched osprey eggs collected in Sweden (1966–2013), and there was a similar decreasing trend [53] (Table 3D). The present first trimester data were also close to the *p*,*p’*-DDE range reported in six unhatched osprey eggs collected in Northern Quark in 1970–1971 [21]. In North American osprey populations *p*,*p’*-DDE concentrations were clearly higher in 1991–2002 than in our second trimester samples, and in many cases even higher than in our first trimester samples [41, 42, 46–49], and only in a few reference sites the levels were similar [43, 48]. *p*,*p’*-DDD concentrations showed a similar pattern, but *p*,*p’*-DDT was mainly not detectable in our samples. Our second trimester *p*,*p’*-DDE concentrations were slightly higher than those from the Menorca Island (Spain, 1994–2000), but *p*,*p’*-DDT concentrations were higher in Menorca, possibly due to more recent DDT contamination.

The proportion of *p*,*p’*-DDD during the first and the third trimester was 17.5% and 8.2% of *p*,*p’*-DDE, respectively, which is quite similar to those reported for white-tailed sea eagle eggs from Sweden, i.e. 17% in 1960s-1970s and <4% in 1990s [45]. Similarly, the proportion of *p*,*p’*-DDD was 4.6% of *p*,*p’*-DDE based on median concentrations in osprey eggs from Sweden [53]. In the present study the proportion of *p*,*p’*-DDT was 0.12% of the amount of *p*,*p’*-DDE during the second trimester, which is lower than the proportion reported from Wisconsin (USA, 1992–1993), i.e. 0.83–1.15% [43], which suggests more recent DDT contamination in the latter location.

Egg shell thinning (as also indicated by the related parameters desiccation index and shell index) is the classical harmful effect that is mainly linked to DDE exposure of ospreys and other raptors [45, 46, 76, 93]. Studies of Wiemeyer et al. [76] indicated that shell thinning by 10% was associated with DDE concentration of 2000 ng/g ww, 15% with 4200 ng/g ww and 20% with 8700 ng/g ww. Based on these observations the critical threshold for shell thinning and related reproductive success in ospreys was set to 4200 ng/g ww. Thus, in the present study nearly all analyzed eggs since 1985 were below the critical threshold (S12, S16 Figs) and therefore not likely to have been affected by characterized adverse effects of DDT.

## Summary and conclusions

This study showed that concentrations of all chlorinated compounds in osprey eggs decreased significantly during 1972–2017 in all study areas (∑PCN only partly). Average annual decreases were ∑PCDD/F 2.3–4.9%, ∑PCB 2.2–4.2%, ∑PCN 3.7–5.7% and ∑DDT 7.1–9.5%. Instead, concentrations of the brominated compounds ∑PBB and ∑BDE increased significantly peaking in 1990s and declining rapidly thereafter. Temporal trends in osprey eggs reflected quite well the available data on POP levels in the main prey fish pike and bream, but also in other fish, such as Baltic herring.

In spite of some differences, POP concentrations in osprey eggs analyzed in this study were comparable to those from other studies and areas. Comparison of POP concentrations between osprey and white-tailed sea eagle eggs from same or closely located areas revealed consistently higher levels in sea eagle eggs than in osprey eggs. This has been reported earlier [21], and is explained by longer lifetime and higher trophic level of sea eagles that feed also waterfowls. In addition, ospreys migrate, and sea eagles live all the time close to their (often more industrialized and contaminated) nesting ground.

The highest sum concentrations of most compound groups were in osprey eggs from Lake Vanajanselkä. The high levels are explained by long-term industrial emissions since 1870s, but also the dominance of bream as a prey of ospreys in this area. According to the database for fish contaminants the bream has higher concentrations of lipophilic POPs than in the pike and other predatory fish, which is most likely due to the higher fat content of bream. There were obvious differences in PCDD/F congener profiles among study areas, even between the closely located Lake Vanajanselkä and Pristine SW Lake Area. The observed spatial differences in POP levels and congener profiles in eggs suggest specific sources of exposure and emphasize the contribution of exposure from nesting area as denoted before for PCDD/Fs and DDE [27, 28]. Based on long-term ring encounters migration routes and wintering areas of ospreys nesting in our study areas did not differ, and the main wintering area is in West Africa, in areas that are generally less industrialized. This is in agreement with earlier findings [29]. There are no data on elimination half-lives of POPs in ospreys, but the half-life of DDE is about 400 days in herring gulls (*Larus argentatus*) [94]. It is therefore likely that at least for the most persistent compounds exposure during the previous nesting season and wintering contribute to the levels in eggs.

The PCDD/F profile was dominated by 2,3,4,7,8-PeCDF, except in Lake Vanajanselkä where 1,2,3,6,7,8-HxCDD dominated. Congener profiles of osprey eggs quite closely reflected those of prey fish except that 2,3,7,8-TCDF had a lower proportion and 1,2,3,6,7,8-HxCDD a higher proportion than in prey fish. Profiles of other congener groups were quite similar across different study areas. The PCB congener profile was dominated by PCB 153 in all study areas, followed by PCB 180 and PCB 138. Among dioxin-like compounds PCBs accounted for 82%, PCDDs 14% and PCDFs 4% of ∑TEQ, and PCB 126 made the greatest contribution to ∑TEQ in all study areas (62–72%), followed by 1,2,3,7,8-PeCDD (6–11%). The BDE congener profile was dominated by BDE 47, followed by BDE 100. ∑DDT concentrations were quite similar across all study areas with DDE contributing about 90% of ∑DDT.

Decreased survival of embryos is considered the most sensitive toxic effects of dioxin-like compounds in birds. Based on the observed ∑TEQ levels in osprey eggs that exceed the NOAEL of 136 pg TEQ/g ww for embryonic mortality it is likely that maternal exposure to dioxin-like compounds has decreased embryonic survival among highly exposed ospreys especially in Lake Vanajanselkä and Northern Quark prior to 2010. Accordingly, embryonic mortality due to these compounds is the probable explanation for decreased annual average production of chicks per active nest in Lake Vanajanselkä. Eggshell thinning is the classical harmful effect of DDE exposure of ospreys and other raptors. In this study nearly all analyzed eggs since 1985 were below the critical threshold of 4200 ng/g ww, and therefore this characteristic adverse effect of DDT seems very unlikely since that. Also, adverse effects on reproduction due to BDEs are highly improbable, because all ∑BDE levels observed in this study were below the level of 1000 ng/g ww reported to cause adverse effects on reproduction.

In the early 1970s when the Finnish program for monitoring osprey populations was launched the number of ospreys had collapsed in several countries, and it was low also in Finland [31]. Since 1975 the number of active nests has doubled in Finland, and the increase between 1975 and 1995 was 57%, and between 1995 and 2010 27%. As shown in this study, the recovery of Finnish osprey populations has taken place in line with a general decrease in environmental levels of most POPs due to both global and national risk management actions.

Overall, this study emphasizes the value of osprey as a sentinel species for studying and monitoring contaminants in aquatic ecosystems as suggested earlier [26]. Furthermore, this study highlights the significance of long-term time series, not only for environmental monitoring but also for nature conservation and maintaining biodiversity. It was hardly possible to imagine all potential dimensions and future uses of the Project Pandion for monitoring osprey populations, implemented by bird ringers, when it was launched in the early 1970s.

## Supporting information

S1 File A complete list of egg samples indicating the collection year, area, and results of analyses. Osprey chick productivity data.
(XLSX)

S2 File Analytical methods, toxic equivalency factors and IUPAC names of numbered congeners. (PDF)

S1 Fig An osprey nest with a large chick and an unhatched egg (photo P. Saurola). (TIF)

S2 Fig Individual data and box plots on ∑PCDD/F and PCDD/F-TEQs of all samples. In left panel ∑PCDD/F (A) and PCDD/F TEQ (C) show decreasing trends since 1980’s-1990’s until 2010. In right panel ∑PCDD/F (B) and PCDD/F TEQ (D) means (x), medians (horizontal line), interquartile ranges, min and max datapoints excluding outliers (exceeding 1.5 times the interquartile range; bars) and individual values of all samples (1972–2017) in different study areas are shown. ∑PCDD/F and PCDD/F TEQ followed the same pattern, and the highest levels were in Lake Vanajanselkä and Northern Quark and in a few outliers in Pristine SW Lake Area.
(TIF)

S3 Fig Linear regression between PCDD/F TEQ and ∑PCDD/F in different study areas. The correlation was very good in Finnish Archipelago Sea followed by Lake Vanajanselkä and Northern Quark, but poor in Pristine SW Lake Area.
(TIF)

S4 Fig Individual data and box plots on ∑PCB and PCB TEQs of all samples. In left panel ∑PCB (A) and PCB TEQ (C) show decreasing temporal trends that depend on study area until 2010. In right panel ∑PCB (B) and PCB TEQ (D) means (x), medians (horizontal line), interquartile ranges, min and max datapoints excluding outliers (exceeding 1.5 times the interquartile range; bars) and individual values of all samples (1972–2017) in different study areas are shown. Similarly with PCDD/Fs, ∑PCB and PCB TEQ followed the same pattern, and the highest levels were in Lake Vanajanselkä and Northern Quark and in a few outliers in Pristine SW Lake Area.
(TIF)

S5 Fig Linear regression between ∑PCB and ∑indicator PCB in different study areas. The correlation was very strong in all study areas, which confirms the validity of the indicator PCBs. Indicator PCBs (PCBs 28, 52, 101, 138, 153, 180) have been selected to represent the most abundant congeners across the compositional range of most common technical mixtures and the environment.
(TIF)

S6 Fig Linear regression between PCB TEQ and ∑PCB in different study areas. The correlation was very good in Pristine SW Lake Area and in Finnish Archipelago Sea and slightly weaker in Lake Vanajanselkä and Northern Quark.
(TIF)

S7 Fig Individual data and box plots on ∑TEQs of all samples. Individual data show decreasing temporal trends that depend on study area until 2010 (A). PCDD/F TEQ + PCB TEQ means (x), medians (horizontal line), interquartile ranges, min and max data points excluding outliers (exceeding 1.5 times the interquartile range; bars) and individual values of all samples (1972–2017) in different study areas (B). The highest levels were in Lake Vanajanselkä and Northern Quark and in a few outliers in Pristine SW Lake Area.
(TIF)

S8 Fig Box plot on proportions of PCB TEQ on ∑TEQ. Means (x), medians (horizontal line), interquartile ranges, min and max datapoints excluding outliers (exceeding 1.5 times the interquartile range) and individual values of all samples (1972–2017) in different study areas. PCB TEQs contribute over 80% of ∑TEQ in all study areas and the highest proportion of PCB TEQs is in Lake Vanajanselkä.
(TIF)

S9 Fig Linear regression between PCB TEQ and PCDD/F TEQ in different study areas. The correlation was very good in Finnish Archipelago Sea and quite good in Northern Quark, but weaker in Lake Vanajanselkä and in Pristine SW Lake Area.
(TIF)

S10 Fig Individual data and box plots on ∑PBB of all samples. Individual data (A) indicate an increase in the early 1980’s, a peak in mid-late 1990’s followed by a decrease depending on study area. ∑PBB means (x), medians (horizontal line), interquartile ranges, min and max data points excluding outliers (exceeding 1.5 times the interquartile range; bars) and individual values of all samples (1972–2005) in different study areas (B). The highest levels were in Northern Quark and in outliers in Pristine SW Lake Area.
(TIF)

S11 Fig Individual data and box plots on ∑BDE of all samples. Individual data (A) show an increase in mid-1980’s, peak in late 1990’s and decline rapidly before 2010. ∑BDE means (x), medians (horizontal line), interquartile ranges, min and max data points excluding outliers (exceeding 1.5 times the interquartile range; bars) and individual values of all samples (1972–2017) in different study areas (B). The highest levels were in Lake Vanajanselkä and in a few outliers in Pristine SW Lake Area.
(TIF)

S12 Fig Individual data and box plots on ∑PCN of all samples. Individual data (A) indicate no major temporal trends. ∑PCN means (x), medians (horizontal line), interquartile ranges, min and max data points excluding outliers (exceeding 1.5 times the interquartile range; bars) and individual values of all samples in different study areas during 1972–2006 (B). Overall, the levels were quite similar in all study areas.
(TIF)

S13 Fig Individual data and box plots on ∑DDT of all samples. Individual data in different study areas (A) indicating a steady decline until the late 1980’s followed by a minor decline until early 2000. ∑DDT means (x), medians (horizontal line), interquartile ranges, min and max data points excluding outliers (exceeding 1.5 times the interquartile range; bars) and individual values of all samples (1972–2006) in different study areas (B). There were high individual values in all study areas (highest in Lake Vanajanselkä) until the late 1990’s after which the levels were quite similar.
(TIF)

S14 Fig Annual average production of chicks per active nest in the Pristine SW Lake Area (blue triangles) and in Lake Vanajanselkä (red dots connected with a line) during 1972–2017. In 46 years, productivity has been lower in the Pristine SW Lake Area than in the Lake Vanajanselkä only in nine years (marked with circles). The difference is statistically significant (p<0.001, Paired T-test). Data from the database of the Finnish Museum of Natural History.
(TIF)

S15 Fig Average productivity of the Finnish ospreys in 1971–2022. Black square = chicks / occupied territory, blue triangle = chicks / active nest, red dot = chicks / successful nest. Data from the database of the Finnish Museum of Natural History [31].
(TIF)

S16 Fig Ring encounters of ospreys ringed in the study areas of the present study in 1957–2023. Black dots are ringing sites and red dots encounter sites. Locations of ring encounters of ospreys ringed in areas of Northern Quark and Finnish Archipelago Sea did not significantly differ from ospreys ringed in areas of Lake Vanajanselkä and Pristine SW Lake Area. Data from the Finnish Museum of Natural History. The map was drawn by the TISS application developed by AB based on coordinates from the CIA public domain database.
(TIF)

S1 Table A dataset on prey fish species of ospreys in Northern Quark and Vanajanselkä area. Data from M. Finnlund and P. Saurola. Difference between bream and pike and between cyprinid and predatory fish is statistically significant (p<0.001, Fischer’s Exact Test).
(DOCX)

## References

[1] Stockholm Convention on Persistent Organic Pollutants. 2021. Available: http://www.pops.int/

[2] HELCOM Thematic assessment of hazardous substances 2011–2016. Baltic Sea Environment Proceedings. The Baltic Marine Environment Protection Commission–HELCOM; 2018. Available: http://www.helcom.fi/baltic-sea-trends/holistic-assessments/state-of-the-baltic-sea-2018/reports-and-materials/

[3] Helander B, Olsson M, Reutergårdh L. Residue levels of organochlorine and mercury compounds in unhatched eggs and the relationships to breeding success in white-tailed sea eagles Haliaeetus albicilla in Sweden. Ecography. 1982;5: 349–366. doi: 10.1111/j.1600-0587.1982.tb01049.x

[4] Bignert A, Danielsson S, Nyberg E, Asplund L, Eriksson U, Nylund K, et al. Comments Concerning the National Swedish Contaminant Monitoring Programme in Marine Biota, 2010. Swedish Museum of Natural History; 2010. Available: https://www.nrm.se/download/18.1c3523612b9bef904d80001896/

[5] Airaksinen R, Hallikainen A, Rantakokko P, Ruokojärvi P, Vuorinen PJ, Parmanne R, et al. Time trends and congener profiles of PCDD/Fs, PCBs, and PBDEs in Baltic herring off the coast of Finland during 1978–2009. Chemosphere. 2014;114: 165–171. doi: 10.1016/j.chemosphere.2014.03.097 25113198

[6] Bignert A, Danielsson S, Ek C, Faxneld S, Nyberg E. Comments Concerning the National Swedish Contaminant Monitoring Programme in Marine Biota, 2017 (2016 years data). Swedish Museum of Natural History; 2017. Available: https://www.diva-portal.org/smash/get/diva2:1185621/FULLTEXT01.pdf

[7] Commission Regulation (EC) No 1881/2006 of 19 December 2006 setting maximum levels for certain contaminants in foodstuffs (Text with EEA relevance). Official Journal of the European Union. 2006;L: (OJ L 364 20.12.2006, 5).

[8] COMMISSION RECOMMENDATION (EU) 2016/688 of 2 May 2016 on the monitoring and management of the presence of dioxins and PCBs in fish and fishery products from the Baltic region (Text with EEA relevance). Official Journal of the European Union. 2016;L 118: 16–23.

[9] Tuomisto J. Dioxins and dioxin-like compounds: toxicity in humans and animals, sources, and behaviour in the environment. WikiJournal of Medicine 6(1):8. 2019. doi: 10.15347/WJM/2019.008

[10] Tuomisto J, Viluksela M. A Handbook of Environmental Toxicology. Human Disorders and Ecotoxicology (D’Mello J.P.F. ed.). 13. Dioxins II. Human exposure and health risks. Oxfordshire: CAB International; 2020.

[11] Van den Berg M, Birnbaum LS, Denison M, De Vito M, Farland W, Feeley M, et al. The 2005 World Health Organization reevaluation of human and Mammalian toxic equivalency factors for dioxins and dioxin-like compounds. Toxicol Sci. 2006;93: 223–241. doi: 10.1093/toxsci/kfl055 16829543 PMC2290740

[12] Alarcon S, Esteban J, Roos R, Heikkinen P, Sanchez-Perez I, Adamsson A, et al. Endocrine, metabolic and apical effects of in utero and lactational exposure to non-dioxin-like 2,2’,3,4,4’,5,5’-heptachlorobiphenyl (PCB 180): A postnatal follow-up study in rats. ReprodToxicol. 2021;102: 109–127. doi: 10.1016/j.reprotox.2021.04.004 33992733

[13] Environmental Health Criteria 152: Polybrominated biphenyls: IPCS International Programme on Chemical Safety. Geneva: United Nations Environment Programme, International Labour Organisation, World Health Organization; 1994. Available: https://www.inchem.org/documents/ehc/ehc/ehc152.htm#SectionNumber:1.1

[14] EFSA Panel on Contaminants in the Food Chain (CONTAM). Scientific Opinion on Polybrominated Biphenyls (PBBs) in Food. EFSA J. 2010;8: 1789. doi: 10.2903/j.efsa.2010.1789

[15] Polychlorinated Biphenyls and Polybrominated Biphenyls. IARC MonogrEvalCarcinogRisks Hum. 2016;107: 9–500. 29905442 PMC7681612

[16] Darnerud PO, Eriksen GS, Johannesson T, Larsen PB, Viluksela M. Polybrominated diphenyl ethers: occurrence, dietary exposure, and toxicology. Environ Health Perspect. 109 Suppl 1: 49–68. doi: 10.1289/ehp.01109s149 11250805 PMC1240542

[17] Linares V, Bellés M, Domingo JL. Human exposure to PBDE and critical evaluation of health hazards. Arch Toxicol. 2015;89: 335–356. doi: 10.1007/s00204-015-1457-1 25637414

[18] Falandysz J, Fernandes A, Gregoraszczuk E, Rose M. The toxicological effects of halogenated naphthalenes: A review of aryl hydrocarbon receptor-mediated (dioxin-like) relative potency factors. J Environ Sci Health C Environ Carcinog Ecotoxicol Rev. 2014;32: 239–272. doi: 10.1080/10590501.2014.938945 25226220

[19] Fernandes A, Rose M, Falandysz J. Polychlorinated naphthalenes (PCNs) in food and humans. Environ Int. 2017;104: 1–13. doi: 10.1016/j.envint.2017.02.015 28391007

[20] Fernandes AR, Kilanowicz A, Stragierowicz J, Klimczak M, Falandysz J. The toxicological profile of polychlorinated naphthalenes (PCNs). Sci Total Environ. 837: 155764. doi: 10.1016/j.scitotenv.2022.155764 35545163

[21] Koivusaari J, Laamanen A, Nuuja I, Palokangas R, Vihko V. Notes on the concentrations of some environmental chemicals in the eggs of the white-tailed eagle and the osprey in the Quarken area of the Gulf of Bothnia. Work Environ Health. 1972;9: 44–45.

[22] Bignert A, Olsson M, Persson W, Jensen S, Zakrisson S, Litzen K, et al. Temporal trends of organochlorines in Northern Europe, 1967–1995. Relation to global fractionation, leakage from sediments and international measures. Environ Pollut. 1998;99: 177–198. doi: 10.1016/s0269-7491(97)00191-7 15093312

[23] Bignert A, Helander BO. Monitoring of contaminants and their effects on the common Guillemot and the White-tailed sea eagle. J Ornithol. 2015;156: 173–185. doi: 10.1007/s10336-015-1240-3

[24] Danielsson S, Faxneld S, Soerensen AL. The Swedish National Programme for Contaminants in Marine Biota—Temporal trends and spatial variations. Swedish Museum of Natural History; 2020. Available: https://www.diva-portal.org/smash/get/diva2:1395199/FULLTEXT01.pdf

[25] Poole AF. Ospreys. The revival of a global raptor. Baltimore Md: Johns Hopkins University Press; 2019.

[26] Grove RA, Henny CJ, Kaiser JL. Osprey: worldwide sentinel species for assessing and monitoring environmental contamination in rivers, lakes, reservoirs, and estuaries. J Toxicol EnvironHealth B Crit Rev. 2009;12: 25–44. doi: 10.1080/10937400802545078 19117208

[27] Elliott JE, Machmer MM, Henny CJ, Wilson LK, Norstrom RJ. Contaminants in ospreys from the Pacific Northwest. I. Trends and patterns in polychlorinated dibenzo-p-dioxins and -dibenzofurans in eggs and plasma. Arch Environ Contam Toxicol. 1998;35: 620–631. doi: 10.1007/s002449900424 9776780

[28] Elliott JE, Morrissey CA, Henny CJ, Inzunza ER, Shaw P. Satellite telemetry and prey sampling reveal contaminant sources to Pacific Northwest ospreys. Ecol Appl. 2007;17: 1223–1233. doi: 10.1890/06-1213 17555230

[29] Saurola P. African non-breeding areas of Fennoscandian Ospreys Pandion haliaetus: a ring recovery analysis. Ostrich. 1994;65: 127–136. doi: 10.1080/00306525.1994.9639675

[30] Saurola P. Finnish Project Pandion. Acta Ornithol. 1980;17: 161–168.

[31] Saurola P. Finnish Ospreys Pandion haliaetus 1971–2020: 86–93 (in Finnish with English summary). Linnut Yearbook. 2021;2020: 86–93.

[32] Herzke D, Kallenborn R, Nygard T. Organochlorines in egg samples from Norwegian birds of prey: congener-, isomer- and enantiomer specific considerations. Sci Total Environ. 2002;291: 59–71. doi: 10.1016/s0048-9697(01)01092-0 12150443

[33] Lokki H, Saurola P. Bootstrap methods for two-sample location and scatter problems. Acta Ornithol. 17: 161–168.

[34] Lokki H, Saurola P. Comparing timing and routes of migration based on ring encounters and randomization methods. Anim Biodivers Conserv. 2004;27: 357–368.

[35] Bignert A. PIA statistical application developed for use by the Arctic Monitoring and Assessment Programme. www.amap.no; 2007.

[36] Gilbert RO. Statistical methods for environmental pollution monitoring. New York Chichester Weinheim: Wiley; 1987.

[37] Report of the Joint Meeting of The Working Group on Environmental Assessment and Monitoring Strategies and The Working Group on Statistical Aspects of Environmental Monitoring. Copenhagen: International Council for the Exploration of the Sea; 1995 pp. 1–8. Report No.: ICES CM 1995/ENV:7 Ref.: D+E. Available: https://imr.brage.unit.no/imr-xmlui/bitstream/handle/11250/105388/CM_1995_ENV_7.pdf?sequence=1&isAllowed=y

[38] Nicholson MD, Fryer RJ, Larsen JR. Temporal trend monitoring: Robust method for analysing contaminant trend monitoring data. Copenhagen: International Council for the Exploration of the Sea; 1998 pp. 1–23. Report No.: No. 20. Available: https://repository.oceanbestpractices.org/bitstream/handle/11329/712/TIMES20.pdf?sequence=1&isAllowed=y

[39] Nordlöf U, Helander B, Zebühr Y, Bignert A, Asplund L. Polychlorinated dibenzo-p-dioxins, polychlorinated dibenzofurans and non-ortho-PCBs in eggs of white-tailed sea eagles collected along the Swedish coast in the Baltic Sea. Sci Total Environ. 2012;438: 166–173. doi: 10.1016/j.scitotenv.2012.07.016 23000468

[40] Jiménez B, Merino R, Abad E, Rivera J, Olie K. Evaluation of Organochlorine Compounds (PCDDs, PCDFs, PCBs and DDTs) in Two Raptor Species Inhabiting a Mediterranean Island in Spain (8 pp). Environ Sci Pollut Res Int. 2007;14: 61–68. doi: 10.1065/espr2006.01.015 21959542

[41] Henny CJ, Kaiser JL, Grove RA. PCDDs, PCDFs, PCBs, OC pesticides and mercury in fish and osprey eggs from Willamette River, Oregon (1993, 2001 and 2006) with calculated biomagnification factors. Ecotoxicology. 2009;18: 151–173. doi: 10.1007/s10646-008-0268-z 18830817

[42] Henny CJ, Grove RA, Kaiser JL. Osprey distribution, abundance, reproductive success and contaminant burdens along lower Columbia River, 1997/1998 versus 2004. Arch Environ Contam Toxicol. 2008;54: 525–534. doi: 10.1007/s00244-007-9041-1 17926083

[43] Woodford JE (U, Karasov WH, Meyer MW, Chambers L. Impact of 2,3,7,8-TCDD exposure on survival, growth, and behavior of ospreys breeding in Wisconsin, USA. Environ Toxicol Chem. 1998;17: 1323–1331. doi: 10.1002/etc.5620170717

[44] Braune BM, Mallory ML. Declining trends of polychlorinated dibenzo-p-dioxins, dibenzofurans and non-ortho PCBs in Canadian Arctic seabirds. Environ Pollut. 2017;220: 557–566. doi: 10.1016/j.envpol.2016.10.003 27742441

[45] Helander B, Olsson A, Bignert A, Asplund L, Litzén K. The Role of DDE, PCB, Coplanar PCB and Eggshell Parameters for Reproduction in the White-tailed Sea Eagle (Haliaeetus albicilla) in Sweden. Ambio. 2002;31: 386–403. doi: 10.1579/0044-7447-31.5.386 12374047

[46] Elliott JE, Machmer MM, Wilson LK, Henny CJ. Contaminants in Ospreys from the Pacific Northwest: II. Organochlorine Pesticides, Polychlorinated Biphenyls, and Mercury, 1991–1997. Arch Environ Contam Toxicol. 2000;38: 93–106. doi: 10.1007/s002449910012 10556376

[47] Martin PA, De Solla SR, Ewins P. Chlorinated hydrocarbon contamination in osprey eggs and nestlings from the Canadian Great Lakes basin, 1991–1995. Ecotoxicology. 2003;12: 209–224. doi: 10.1023/a:1022554810870 12739869

[48] Rattner BA, McGowan PC, Golden NH, Hatfield JS, Toschik PC, Lukei RF, et al. Contaminant Exposure and Reproductive Success of Ospreys (Pandion haliaetus) Nesting in Chesapeake Bay Regions of Concern. Arch Environ Contam Toxicol. 2004;47: 126–140. doi: 10.1007/s00244-003-3160-0 15346786

[49] Toschik PC, Rattner BA, McGowan PC, Christman MC, Carter DB, Hale RC, et al. Effects of contaminant exposure on reproductive success of ospreys (Pandion haliaetus) nesting in Delaware River and Bay, USA. Environ Toxicol Chem. 2005;24: 617–628. doi: 10.1897/04-141r.1 15779762

[50] Herzke D, Berger U, Kallenborn R, Nygard T, Vetter W. Brominated flame retardants and other organobromines in Norwegian predatory bird eggs. Chemosphere. 2005;61: 441–449. doi: 10.1016/j.chemosphere.2005.01.066 16182862

[51] Vetter W, von der Recke R, Herzke D, Nygard T. Detailed analysis of polybrominated biphenyl congeners in bird eggs from Norway. Environ Pollut. 2008;156: 1204–1210. doi: 10.1016/j.envpol.2008.04.003 18472199

[52] Nordlöf U, Helander B, Bignert A, Asplund L. Levels of brominated flame retardants and methoxylated polybrominated diphenyl ethers in eggs of white-tailed sea eagles breeding in different regions of Sweden. Sci Total Environ. 2010;409: 238–246. doi: 10.1016/j.scitotenv.2010.09.042 20971499

[53] Geng D, Jogsten IE Kukucka P, Roos A. Temporal trends of polychlorinated biphenyls, organochlorine pesticides and polybrominated diphenyl ethers in osprey eggs in Sweden over the years 1966–2013. Organohalogen Compounds. 2016;78: 524–527.

[54] Henny CJ, Kaiser JL, Grove RA, Johnson BL, Letcher RJ. Polybrominated diphenyl ether flame retardants in eggs may reduce reproductive success of ospreys in Oregon and Washington, USA. Ecotoxicology. 2009;18: 802–813. doi: 10.1007/s10646-009-0323-4 19513829

[55] Järnberg U, Asplund L, de Wit C, Egebäck A, Wideqvist U, Jakobsson E. Distribution of Polychlorinated Naphthalene Congeners in Environmental and Source-Related Samples. Arch Environ Contam Toxicol. 1997;32: 232–245. doi: 10.1007/s002449900181 9096072

[56] Braune BM, Muir DCG. Declining trends of polychlorinated naphthalenes in seabird eggs from the Canadian Arctic, 1975–2014. Environ Sci Technol. 2017;51: 3802–3808. doi: 10.1021/acs.est.7b00431 28333458

[57] Kansanen PH, Aho J. Changes in the macrozoobenthos associations of polluted Lake Vanajavesi, Southern Finland, over a period of 50 years. Ann Zool Fennici. 1981;18: 73–101.

[58] Isosaari P, Hallikainen A, Kiviranta H, Vuorinen PJ, Parmanne R, Koistinen J, et al. Polychlorinated dibenzo-p-dioxins, dibenzofurans, biphenyls, naphthalenes and polybrominated diphenyl ethers in the edible fish caught from the Baltic Sea and lakes in Finland. Environ Pollut. 2006;141: 213–225. doi: 10.1016/j.envpol.2005.08.055 16226362

[59] Finnish Institute for Health and Welfare. Database for fish contaminants. 2024. Available: https://thl.fi/en/topics/environmental-health/environmental-pollutants/database-for-fish-contaminants

[60] Hallikainen A, Kiviranta H, Isosaari P, Vartiainen T, Parmanne R, Vuorinen PJ. Concentration of dioxins, furans, dioxin-like PCB compounds and polybrominated diphenyl ethers in domestic fresh water and salt water fish (in Finnish). Helsinki: National Food Agency of Finland; 2004. Available: https://urn.fi/URN:ISBN:951-732-206-2

[61] Hallikainen A, Airaksinen R, Rantakokko P, Koponen J, Mannio J, Vuorinen PJ, et al. Environmental pollutants in Baltic fish and other domestic fish: PCDD/F, PCB, PBDE, PFC and OT compounds (in Finnish). Helsinki: Finnish Food Safety Authority Evira; 2011. Available: https://urn.fi/URN:ISBN:978-952-225-083-4

[62] Airaksinen R, Jestoi M, Keinänen M, Kiviranta H, Koponen J, Mannio J, et al. Changes in the levels of environmental contaminants of Finnish wild caught fish (in Finnish). Helsinki: Prime Minister´s Office; 2018. Available: http://urn.fi/URN:ISBN:978-952-287-600-3

[63] Miller A, Nyberg E, Danielsson S, Faxneld S, Haglund P, Bignert A. Comparing temporal trends of organochlorines in guillemot eggs and Baltic herring: Advantages and disadvantage for selecting sentinel species for environmental monitoring. Mar Environ Res. 2014;100: 38–47. doi: 10.1016/j.marenvres.2014.02.007 24680644

[64] Kiviranta H, Purkunen R, Vartiainen T. Levels and trends of PCDD/Fs and PCBs in human milk in Finland. Chemosphere. 1999;38: 311–323. doi: 10.1016/s0045-6535(98)00192-1 10901657

[65] Fång J, Nyberg E, Bignert A, Bergman Å. Temporal trends of polychlorinated dibenzo-p-dioxins and dibenzofurans and dioxin-like polychlorinated biphenyls in mothers’ milk from Sweden, 1972–2011. Environ Int. 2013;60: 224–231. doi: 10.1016/j.envint.2013.08.019 24080458

[66] Rappe C, Marklund S, Kjeller L-O, Lindskog A. Long-range transport of PCDDs and PCDFs on airborne particles. Chemosphere. 1989;18: 1283–1290. doi: 10.1016/0045-6535(89)90266-X

[67] Wakimoto T, Kannan N, Ono M, Tatsukawa R, Masuda Y. Isomer-specific determination of polychlorinated dibenzofurans in Japanese and American polychlorinated biphenyls. Chemosphere. 1988;17: 743–750. doi: 10.1016/0045-6535(88)90254-8

[68] Vartiainen T, Lampi P, Tolonen K, Tuomisto J. Polychlorodibenzo-p-dioxin and polychlorodibenzofuran concentrations in lake sediments and fish after a ground water pollution with chlorophenols. Chemosphere. 1995;30: 1439–1451. doi: 10.1016/0045-6535(95)00037-9 7743140

[69] Rappe C, Kjeller L-O, Kulp S-E, De Wit C, Hasselsten I, Palm O. Levels, profile and pattern of PCDDs and PCDFs in samples related to the production and use of chlorine. Chemosphere. 1991;23: 1629–1636. doi: 10.1016/0045-6535(91)90010-B

[70] Assefa A, Tysklind M, Bignert A, Josefsson S, Wiberg K. Sources of polychlorinated dibenzo-p-dioxins and dibenzofurans to Baltic Sea herring. Chemosphere. 2019;218: 493–500. doi: 10.1016/j.chemosphere.2018.11.051 30497032

[71] Miller A, Hedman JE, Nyberg E, Haglund P, Cousins IT, Wiberg K, et al. Temporal trends in dioxins (polychlorinated dibenzo-p-dioxin and dibenzofurans) and dioxin-like polychlorinated biphenyls in Baltic herring (Clupea harengus). Mar Pollut Bull. 2013;73: 220–230. doi: 10.1016/j.marpolbul.2013.05.015 23806670

[72] Rebryk A, Gallampois C, Haglund P. A time-trend guided non-target screening study of organic contaminants in Baltic Sea harbor porpoise (1988–2019), guillemot (1986–2019), and white-tailed sea eagle (1965–2017) using gas chromatography–high-resolution mass spectrometry. Sci Total Environ. 2022;829: 154620. doi: 10.1016/j.scitotenv.2022.154620 35306077

[73] Clark KE, Stansley W, Niles LJ. Changes in Contaminant Levels in New Jersey Osprey Eggs and Prey, 1989 to 1998. Arch Environ Contam Toxicol. 2001;40: 277–284. doi: 10.1007/s002440010173 11243331

[74] Jansson B, Andersson R, Asplund L, Litzen K, Nylund K, Sellström U, et al. Chlorinated and brominated persistent organic compounds in biological samples from the environment. EnvironToxicolChem. 1993;12: 1163–1174. doi: 10.1002/etc.5620120704

[75] Henny CJ, Kaiser JL, Grove RA, Bentley VR, Elliott JE. Biomagnification factors (fish to Osprey eggs from Willamette River, Oregon, U.S.A.) for PCDDs, PCDFs, PCBs and OC pesticides. Environ Monit Assess. 2003;84: 275–315. doi: 10.1023/a:1023396815092 12807265

[76] Wiemeyer SN, Bunck CM, Krynitsky AJ. Organochlorine pesticides, polychlorinated biphenyls, and mercury in osprey eggs—1970–79—and their relationships to shell thinning and productivity. Arch Environ Contam Toxicol. 1988;17: 767–787. doi: 10.1007/BF01061982

[77] Elliott JE, Wilson LK, Henny CJ, Trudeau SF, Leighton FA, Kennedy SW, et al. Assessment of biological effects of chlorinated hydrocarbons in osprey chicks. Environ Toxicol Chem. 2001;20: 866–879. doi: 10.1002/etc.5620200423 11345464

[78] Karchner SI, Franks DG, Kennedy SW, Hahn ME. The molecular basis for differential dioxin sensitivity in birds: Role of the aryl hydrocarbon receptor. Proc Natl Acad Sci USA. 2006;103: 6252–6257. doi: 10.1073/pnas.0509950103 16606854 PMC1435364

[79] Farmahin R, Manning GE, Crump D, Wu D, Mundy LJ, Jones SP, et al. Amino Acid Sequence of the Ligand-Binding Domain of the Aryl Hydrocarbon Receptor 1 Predicts Sensitivity of Wild Birds to Effects of Dioxin-Like Compounds. ToxicolSci. 2013;131: 139–152. doi: 10.1093/toxsci/kfs259 22923492

[80] Harris ML, Elliott JE. Effects of polychlorinated biphenyls, dibenzo-p-dioxins and dibenzo- furans and polybrominated diphenyl ethers in birds. 1st ed. In: Beyer WN, Meador J, editors. Environmental Contaminants in Wildlife Interpreting Tissue Concentrations. 1st ed. Boca Raton: CRC Press; 2011. pp. 471–522. doi: 10.1201/b10598

[81] Zhu LY, Hites RA. Temporal trends and spatial distributions of brominated flame retardants in archived fishes from the Great Lakes. Environ Sci Technol. 2004;38: 2779–2784. doi: 10.1021/es035288h 15212250

[82] Sjödin A, Jones RS, Focant J-F, Lapeza C, Wang RY, McGahee EE 3rd, et al. Retrospective time-trend study of polybrominated diphenyl ether and polybrominated and polychlorinated biphenyl levels in human serum from the United States. Environ Health Perspect. 2004;112: 654–658. doi: 10.1289/ehp.112-1241957 15121506 PMC1241957

[83] Vetter W, Gallistl C, Schlienz A, Preston T, Muller J, von der Trenck KT. Brominated flame retardants (BFRs) in eggs from birds of prey from Southern Germany, 2014. Environ Pollut. 2017;231: 569–577. doi: 10.1016/j.envpol.2017.08.047 28843896

[84] Shen H, Main KM, Andersson A-M, Damgaard IN, Virtanen HE, Skakkebaek NE, et al. Concentrations of persistent organochlorine compounds in human milk and placenta are higher in Denmark than in Finland. Hum Reprod. 2008;23: 201–210. doi: 10.1093/humrep/dem199 18025027

[85] Elliott JE, Wilson LK, Wakeford B. Polybrominated Diphenyl Ether Trends in Eggs of Marine and Freshwater Birds from British Columbia, Canada, 1979−2002. Environ Sci Technol. 2005;39: 5584–5591. doi: 10.1021/es050496q 16124290

[86] Airaksinen R, Hallikainen A, Rantakokko P, Ruokojärvi P, Vuorinen PJ, Mannio J, et al. Levels and congener profiles of PBDEs in edible Baltic, freshwater, and farmed fish in Finland. Environ Sci Technol. 49: 3851–3859. doi: 10.1021/es505266p 25699573

[87] Sellstrom U, Bignert A, Kierkegaard A, Haggberg L, de Wit CA, Olsson M, et al. Temporal trend studies on tetra- and pentabrominated diphenyl ethers and hexabromocyclododecane in guillemot egg from the Baltic Sea. Environ Sci Technol. 2003;37: 5496–5501. doi: 10.1021/es0300766 14717156

[88] Lindberg P, Sellström U, Häggberg L, De Wit CA. Higher Brominated Diphenyl Ethers and Hexabromocyclododecane Found in Eggs of Peregrine Falcons (*Falco peregrinus*) Breeding in Sweden. Environ Sci Technol. 2004;38: 93–96. doi: 10.1021/es034614q 14740722

[89] EFSA Panel on Contaminants in the Food Chain (CONTAM). Scientific Opinion on Polybrominated Diphenyl Ethers (PBDEs) in Food. EFSA J. 2011;9. doi: 10.2903/j.efsa.2011.2156 PMC13177364 42148426

[90] Letcher RJ, Marteinson SC, Fernie KJ. Dietary exposure of American kestrels (Falco sparverius) to decabromodiphenyl ether (BDE-209) flame retardant: Uptake, distribution, debromination and cytochrome P450 enzyme induction. Environ Int. 2014;63: 182–190. doi: 10.1016/j.envint.2013.11.010 24317224

[91] Main KM, Kiviranta H, Virtanen HE, Sundqvist E, Tuomisto JT, Tuomisto J, et al. Flame Retardants in Placenta and Breast Milk and Cryptorchidism in Newborn Boys. Environ Health Perspect. 2007;115: 1519–1526. doi: 10.1289/ehp.9924 17938745 PMC2022640

[92] Vorkamp K, Falk K, Møller S, Bossi R, Rigét FF, Sørensen PB. Perfluoroalkyl substances (PFASs) and polychlorinated naphthalenes (PCNs) add to the chemical cocktail in peregrine falcon eggs. Sci Total Environ. 2019;648: 894–901. doi: 10.1016/j.scitotenv.2018.08.090 30144757

[93] Henny CJ, Grove RA, Kaiser JL, Johnson BL. North American osprey populations and contaminants: historic and contemporary perspectives. J Toxicol Environ Health B Crit Rev. 2010;13: 579–603. doi: 10.1080/10937404.2010.538658 21170810

[94] Clark TP, Norstrom RJ, Fox GA, Won HT. Dynamics of organochlorine compounds in herring gulls (*Larus argentatus*): II. A two‐compartment model and data for ten compounds. Environ ToxicolChem. 1987;6: 547–559. doi: 10.1002/etc.5620060707

