# Supplementary material for: Temporal trends (1972–2017) and spatial differences of persistent halogenated aromatic hydrocarbons in osprey eggs in Finland
Source: PLoS One. 2024 Sep 3;19(9):e0308227. doi: 10.1371/journal.pone.0308227 (PMC11371234; doi:10.1371/journal.pone.0308227)
Supplement: S2 File — (PDF) [file pone.0308227.s002.pdf]

S1 Supporting Information. Analytical methods, toxic equivalency factors and IUPAC names of numbered congeners

Osprey eggs collected between 1972-2017 were analyzed for POPs over a relatively wide timeframe in three different campaigns in 2004, 2007 and 2018 (Tables A and B). Sample extraction and cleanup were similar at timepoints. However, GC-MS instrumentation changed as described below.

Table A. Number of eggs analyzed in each POP group and sampling years. The number of different POP congeners analyzed are shown in parenthesis. Lower number of PCN analyses are due to exclusion of samples analyzed before 2005 because of a different cleanup method that proved to result in higher values possible as a consequence of co-eluting PCB peaks that caused severe interference even with the HRMS instrument used.

Persistent organic pollutant group (number of congeners analyzed)	Number of eggs	Sampling years
**Persistent organic pollutants:**		
PCDD/F (17)	164	1972-2017
PCB (37; 12 dioxin-like PCB, 25 NDL-PCB)	164	1972-2017
PBB (19)	115	1972-2005
BDE (15)*	164	1972-2017
PCN (14)	82	1972-2006
DDT and metabolites (6)	147	1972-2006

*BDE 209 not included.

Chemicals and standards

Solvents and solid reagents used were of the highest commercial quality available. Both ^{12}C and ^{13}C standard compounds for POPs were obtained from Wellington or Cambridge Isotope Laboratories. However, the number of ^{13}C-labelled internal standards slightly increased over the years for PCBs (2004: 17; 2018: 23) and BDEs (2004: 6; 2018: 8).

Extraction and clean-up of POPs

Egg samples (2-10 g depending on the availability of sample) were spiked with ^{13}C-labelled internal standards, mixed with concentrated hydrochloric acid (15 ml) and heated to 100°C. After cooling, ultra-purified water (30 ml) was added to the sample and extracted in a separatory funnel with diethyl ether (30 ml). *n*-hexane (30 ml) was added and extraction continued. Organic phase was separated. The extraction of aqueous phase with diethyl ether and *n*-hexane was repeated twice more. The combined organic phase was washed with ultra-purified water. The water fraction was then rejected. The organic phase was dried with sodium sulfate and filtrated through a cotton plug. The solvent was evaporated almost to dryness and reconstituted to pure *n*-hexane before column clean-up. Samples were defatted with multilayer silica column cleanup and fractionated by activated carbon column as previously described [1]. PCBs, PBDEs, PBBs and DDTs were collected from carbon column in the forward eluted fraction 1 (F1) while PCDD/Fs, non-ortho-PCBs and PCNs were collected in backwards eluted fraction 2 (F2). F1 was concentrated to 500 µl of hexane and F2 to 15 µl of nonane before gas chromatography-high resolution mass spectrometry (GC-HRMS) or gas chromatography triple quadrupole mass spectrometer (GC-MS/MS) analysis.

Table B. Groups of POPs and individual congeners analyzed. POPs with detection rates < 20% were excluded from statistical analysis with the exception of PCDD/Fs for which all congeners were included. Congeners marked with '*' had ^{13}C-labelled internal standard.

POP group	Detection rate > 20%	Detection rate < 20%
PCDD/Fs	2378-TCDF*, 12378-PCDF*, 23478-PCDF*, 123478-HxCDF*, 123678-HxCDF*, 234678-HxCDF*, 1234678-HpCDF*, 2378-TCDD*, 12378-PCDD*, 123478-HxCDD*, 123678-HxCDD, 123789-HxCDD*, 1234678-HpCDD*, OCDD*	123789-HxCDF*, 1234789-HpCDF*, OCDF*
PCBs	81*, 77*, 126*, 169*, 18, 28/31*, 33, 47, 49, 51, 52*, 60, 66, 74, 99, 101*, 105*, 110, 114*, 118*, 122, 123*, 128*, 138*, 141, 153*, 156*, 157*, 167*, 170*, 180*, 183, 187, 189, 194*, 206, 209*	-
BDEs	28*, 47*, 66, 75, 77*, 99*, 100*, 119, 153*, 154*,183*	71, 85, 138, 190
PBBs	52*, 75, 101, 103, 153*, 154, 155	18, 22, 29, 31, 37, 38, 49, 53, 56, 77, 80, 169
PCNs	42*, 36, 27*, 52*, 54, 53, 66/67*, 68*, 71/72, 70	48, 73*, 74, 75*
DDTs	p,p'-DDE*, o,p'-DDD, p,p'-DDD, p,p'-DDT*	o,p'-DDE, o,p'-DDT

Instrumental analysis of POPs

In 2004 and 2007 POPs were analyzed with Waters Autospec Ultima GC-HRMS and in 2018 with Agilent 7010 GC-MS/MS. GC column was DB-5MS UI (J&W Scientific, 60m, ID 0.25 mm, 0.25 µm film) in both cases. All groups of POPs were analyzed in separate GC-HRMS or GC-MS/MS run programs except for PCDD/Fs and non-ortho-PCBs that were analyzed in the same run. The status of both GC-HRMS and GC-MS/MS was assessed daily, and the instruments were calibrated and serviced regularly.

Quality control in POP analysis

Two blank samples were included in the batch of samples. Mass of POPs in blank was subtracted from the mass of POPs in real samples. During the years of egg sample analysis, laboratory participated annually to Interlaboratory Comparison of Persistent Organic Pollutants in food (ILC POPs) (https://www.fhi.no/en/studies/ilc-pop/) and since 2008 biannually to interlaboratory comparison of European Union Reference Laboratory for Halogenated Persistent Organic Pollutants in Feed and Food (EURL) (http://www.crl-freiburg.eu/). During the years 2006, 2011, 2012, 2013, 2019 intercomparisons included egg samples analyzed similarly as Osprey eggs. Contaminants analyzed were PCDD/Fs, PCBs and PBDEs in all intercomparisons. For PCNs, same standard solutions and cleanup method were used throughout as those used for the first available intercomparison in 2021 (EURL) for PCNs from Cod Liver Oil. In EURL intercomparison accuracy for congeners PCN-42 and PCN-52/60 that had assigned values, were 97% and 108%. For PBBs and DDTs no interlaboratory comparisons were participated, but technical and quality measures applied in analysis of PBBs and DDTs were similar to those for accredited

POPs.

Laboratory of chemistry at the Finnish Institute for Health and Welfare is an accredited testing laboratory T077 by Finnish Accreditation Services (FINAS) since 1996. Scope of accreditation includes PCDD/Fs, PCBs and PBDE from egg samples. Finnish Institute for Health and Welfare is also the National Reference Laboratory (NRL) for Halogenated POPs in Feed and Food in Finland.

Toxic equivalency factors and nomenclature of congeners

Toxic equivalency factors (TEFs) [2] used for calculation of toxic equivalency quantities (TEQs) of dioxin-like compounds are shown in Table C and IUPAC names of numbered PCB, PBB, BDE and PCN congeners analyzed in this study in Tables D-G.

Table C. WHO 2005 toxic equivalency factors (TEFs) of dioxin-like congeners (Van den Berg et al., 2005)**.**

Congener	WHO 2005 TEF
**PCDDs**	
2,3,7,8-TCDD	1
1,2,3,7,8-PeCDD	1
1,2,3,4,7,8-HxCDD	0.1
1,2,3,6,7,8-HxCDD	0.1
1,2,3,7,8,9-HxCDD	0.1
1,2,3,4,6,7,8-HpCDD	0.01
OCDD	0.0003
**PCDFs**	
2,3,7,8-TCDF	0.1
1,2,3,7,8-PeCDF	0.03
2,3,4,7,8-PeCDF	0.3
1,2,3,4,7,8-HxCDF	0.1
1,2,3,6,7,8-HxCDF	0.1
1,2,3,7,8,9-HxCDF	0.1
2,3,4,6,7,8-HxCDF	0.1
1,2,3,4,6,7,8-HpCDF	0.01
1,2,3,4,7,8,9-HpCDF	0.01
OCDF	0.0003
**Non-ortho-PCBs**	
3,3',4,4'-TCB (77)	0.0001
3,4,4',5-TCB (81)	0.0003
3,3',4,4',5-PeCB (126)	0.1
3,3',4,4',5,5'-HxCB (169)	0.03
**Mono-ortho-PCBs**	
2,3,3',4,4'-PeCB (105)	0.00003
2,3,4,4',5-PeCB (114)	0.00003
2,3',4,4',5-PeCB (118)	0.00003
2',3,4,4',5-PeCB (123)	0.00003
2,3,3',4,4',5-HxCB (156)	0.00003
2,3,3',4,4',5'-HxCB (157)	0.00003
2,3',4,4',5,5'-HxCB (167)	0.00003
2,3,3',4,4',5,5'-HpCB (189)	0.00003

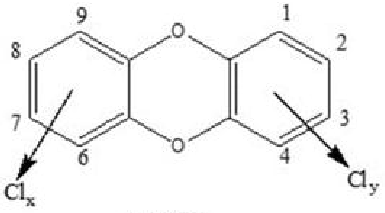

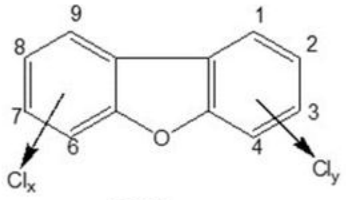

Table D. IUPAC names of numbered PCB congeners.

PCB Congener Number	IUPAC Name	Remarks
PCB 18	2,2',5-Trichlorobiphenyl	
PCB 28	2,4,4'-Trichlorobiphenyl	Indicator PCB
PCB 31	2,4',5-Trichlorobiphenyl	
PCB 33	2,3',4'-Trichlorobiphenyl	
PCB 47	2,2',4,4'-Tetrachlorobiphenyl	
PCB 49	2,2',4,5'-Tetrachlorobiphenyl	
PCB 51	2,2',4,6'-Tetrachlorobiphenyl	
PCB 52	2,2',5,5'-Tetrachlorobiphenyl	Indicator PCB
PCB 60	2,3,4,4'-Tetrachlorobiphenyl	
PCB 66	2,3',4,4'-Tetrachlorobiphenyl	
PCB 74	2,4,4',5-Tetrachlorobiphenyl	
PCB 77	3,3',4,4'-Tetrachlorobiphenyl	Non-ortho PCB
PCB 81	3,4,4',5-Tetrachlorobiphenyl	Non-ortho PCB
PCB 99	2,2',4,4',5-Pentachlorobiphenyl	
PCB 101	2,2',4,5,5'-Pentachlorobiphenyl	Indicator PCB
PCB 105	2,3,3',4,4'-Pentachlorobiphenyl	Mono-ortho PCB
PCB 110	2,3,3',4',6-Pentachlorobiphenyl	
PCB 114	2,3,4,4',5-Pentachlorobipheny	Mono-ortho PCB
PCB 118	2,3',4,4',5-Pentachlorobiphenyl	Mono-ortho PCB
PCB 122	2,3,3',4',5'-Pentachlorobiphenyl	
PCB 123	2,3',4,4',5'-Pentachlorobiphenyl	Mono-ortho PCB
PCB126	3,3',4,4',5-Pentachlorobiphenyl	Non-ortho PCB
PCB 128	2,2',3,3',4,4'-Hexachlorobiphenyl	
PCB 138	2,2',3,4,4',5'-Hexachlorobiphenyl	Indicator PCB
PCB 141	2,2',3,4,5,5'-Hexachlorobiphenyl	
PCB 153	2,2',4,4',5,5'-Hexachlorobiphenyl	Indicator PCB
PCB 156	2,3,3',4,4',5-Hexachlorobiphenyl	Mono-ortho PCB
PCB 157	2,3,3',4,4',5'-Hexachlorobiphenyl	Mono-ortho PCB
PCB 167	2,3',4,4',5,5'-Hexachlorobiphenyl	Mono-ortho PCB
PCB 169	3,3',4,4',5,5'-Hexachlorobiphenyl	Non-ortho PCB
PCB 170	2,2',3,3',4,4',5-Heptachlorobiphenyl	
PCB 180	2,2',3,4,4',5,5'-Heptachlorobiphenyl	Indicator PCB
PCB 183	2,2',3,4,4',5',6-Heptachlorobiphenyl	
PCB 187	2,2',3,4',5,5',6-Heptachlorobiphenyl	
PCB 189	2,3,3',4,4',5,5'-Heptachlorobiphenyl	Mono-ortho PCB
PCB 194	2,2',3,3',4,4',5,5'-Octachlorobiphenyl	
PCB 206	2,2',3,3',4,4',5,5',6-Nonachlorobiphenyl	
PCB 209	Decachlorobiphenyl	

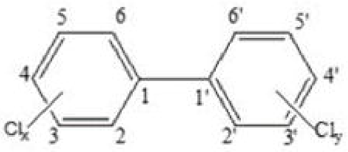

Table E. IUPAC names of numbered PBB congeners.

PBB Congener Number	IUPAC Name	Remarks
PBB 18	2,2',5-Tribromobiphenyl	
PBB 22	2,3,4'-Tribromobiphenyl	
PBB 29	2,4,5-Tribromobiphenyl	
PBB 31	2,4',5-Tribromobiphenyl	
PBB 37	3,4,4'-Tribromobiphenyl	
PBB 38	3,4,5-Tribromobiphenyl	
PBB 49	2,2',4,5'-Tetrabromobiphenyl	
PBB 52	2,2',5,5'-Tetrabromobiphenyl	
PBB 53	2,2',5,6'-Tetrabromobiphenyl	
PBB 56	2,3,3'4'-Tetrabromobiphenyl	Mono-ortho PBB
PBB 75	2,4,4'6-Tetrabromobiphenyl	
PBB 77	3,3',4,4'-Tetrabromobiphenyl	Non-ortho PBB
PBB 80	3,3',5,5'-Tetrabromobiphenyl	Non-ortho PBB
PBB 101	2,2',4,5,5'-Pentabromobiphenyl	
PBB 103	2,2',4,5',6-Pentabromobiphenyl	
PBB 153	2,2',4,4',5,5'-Hexabromobiphenyl	
PBB 154	2,2',4,4',5,6'-Hexabromobiphenyl	
PBB 155	2,2',4,4',6,6'-Hexabromobiphenyl	
PBB 169	3,3',4,4',5,5'-Hexabromobiphenyl	Non-ortho PBB

Table F. IUPAC names of numbered BDE congeners.

BDE Congener Number	IUPAC Name
BDE 28	2,4,4'-Tribromodiphenyl ether
BDE 47	2,2',4,4'-Tetrabromodiphenyl ether
BDE 49	2,2',4,5'-Tetrabromodiphenyl ether
BDE 66	2,3',4,4'-Tetrabromodiphenyl ether
BDE 71	2,3',4',6-Tetrabromodiphenyl ether
BDE 75	2,4,4',6-Tetrabromodiphenyl ether
BDE 77	3,3',4,4'-Tetrabromodiphenyl ether
BDE 85	2,2',3,4,4'-Pentabromodiphenyl ether
BDE 99	2,2',4,4',5-Pentabromodiphenyl ether
BDE 100	2,2',4,4',6-Pentabromodiphenyl ether
BDE 119	2,3',4,4',6-Pentabromodiphenyl ether
BDE 138	2,2',3,4,4',5'-Hexabromodiphenyl ether
BDE 153	2,2',4,4',5,5'-Hexabromodiphenyl ether
BDE 154	2,2',4,4',5,6'-Hexabromodiphenyl ether
BDE 183	2,2',3,4,4',5',6-Heptabromodiphenyl ether
BDE 190	2,3,3',4,4',5,6-Heptabromodiphenyl ether

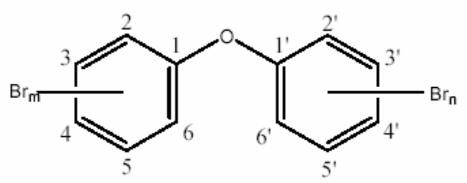

Table G. IUPAC names of numbered PCN congeners.

PCN Congener Number	IUPAC Name
PCN 27	1,2,3,4-Tetrachloronaphthalene
PCN 36	1,2,5,6-Tetrachloronaphthalene
PCN 42	1,3,5,7-Tetrachloronaphthalene
PCN 48	2,3,6,7-Tetrachloronaphthalene
PCN 52	1,2,3,5,7-Pentachloronaphthalene
PCN 53	1,2,3,5,8-Pentachloronaphthalene
PCN 54	1,2,3,6,7-Pentachloronaphthalene
PCN 66	1,2,3,4,6,7-Hexachloronaphthalene
PCN 67	1,2,3,5,6,7-Hexachloronaphthalene
PCN 68	1,2,3,5,6,8-Hexachloronaphthalene
PCN 70	1,2,3,6,7,8-Hexachloronaphthalene
PCN 71	1,2,4,5,6,8-Hexachloronaphthalene
PCN 72	1,2,4,5,7,8-Hexachloronaphthalene
PCN 73	1,2,3,4,5,6,7-Heptachloronaphthalene
PCN 74	1,2,3,4,5,6,8-Heptachloronaphthalene
PCN 75	Octachloronaphthalene

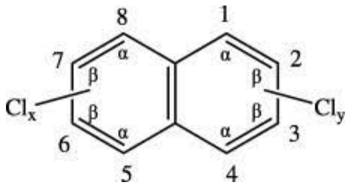

References

1. Kiviranta H, Ovaskainen ML, Vartiainen T. Market basket study on dietary intake of PCDD/Fs, PCBs, and PBDEs in Finland. EnvironInt. 30: 923–932. doi:10.1016/j.envint.2004.03.002

2. Van den Berg M, Birnbaum LS, Denison M, De Vito M, Farland W, Feeley M, et al. The 2005 World Health Organization reevaluation of human and Mammalian toxic equivalency factors for dioxins and dioxin-like compounds. ToxicolSci. 93: 223–241.
