# Supplementary figures and images for: Temporal trends (1972–2017) and spatial differences of persistent halogenated aromatic hydrocarbons in osprey eggs in Finland
Source: PLoS One. 2024 Sep 3;19(9):e0308227. doi: 10.1371/journal.pone.0308227 (PMC11371234; doi:10.1371/journal.pone.0308227)

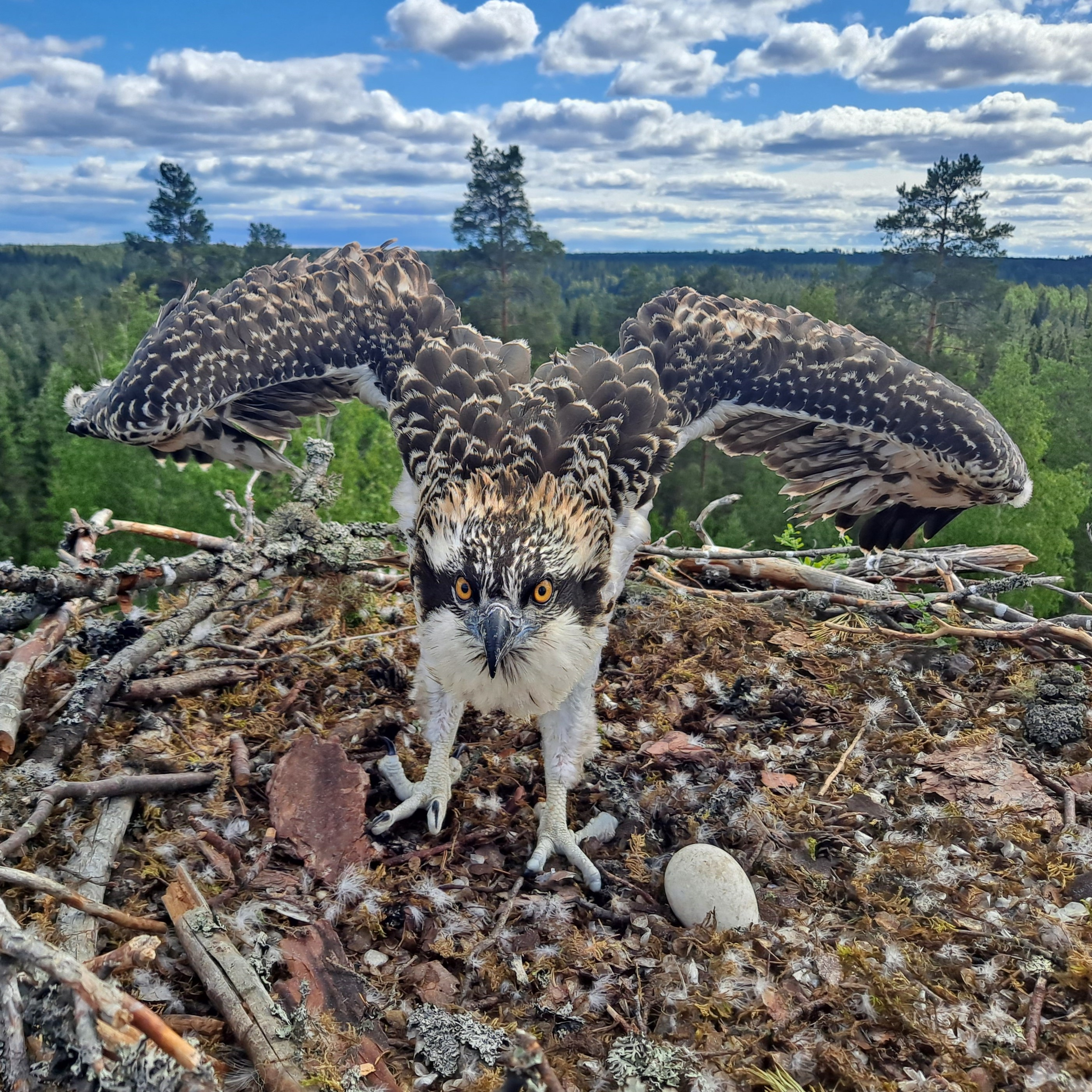

Supplement: S1 Fig — (TIF) [file pone.0308227.s003.tif]

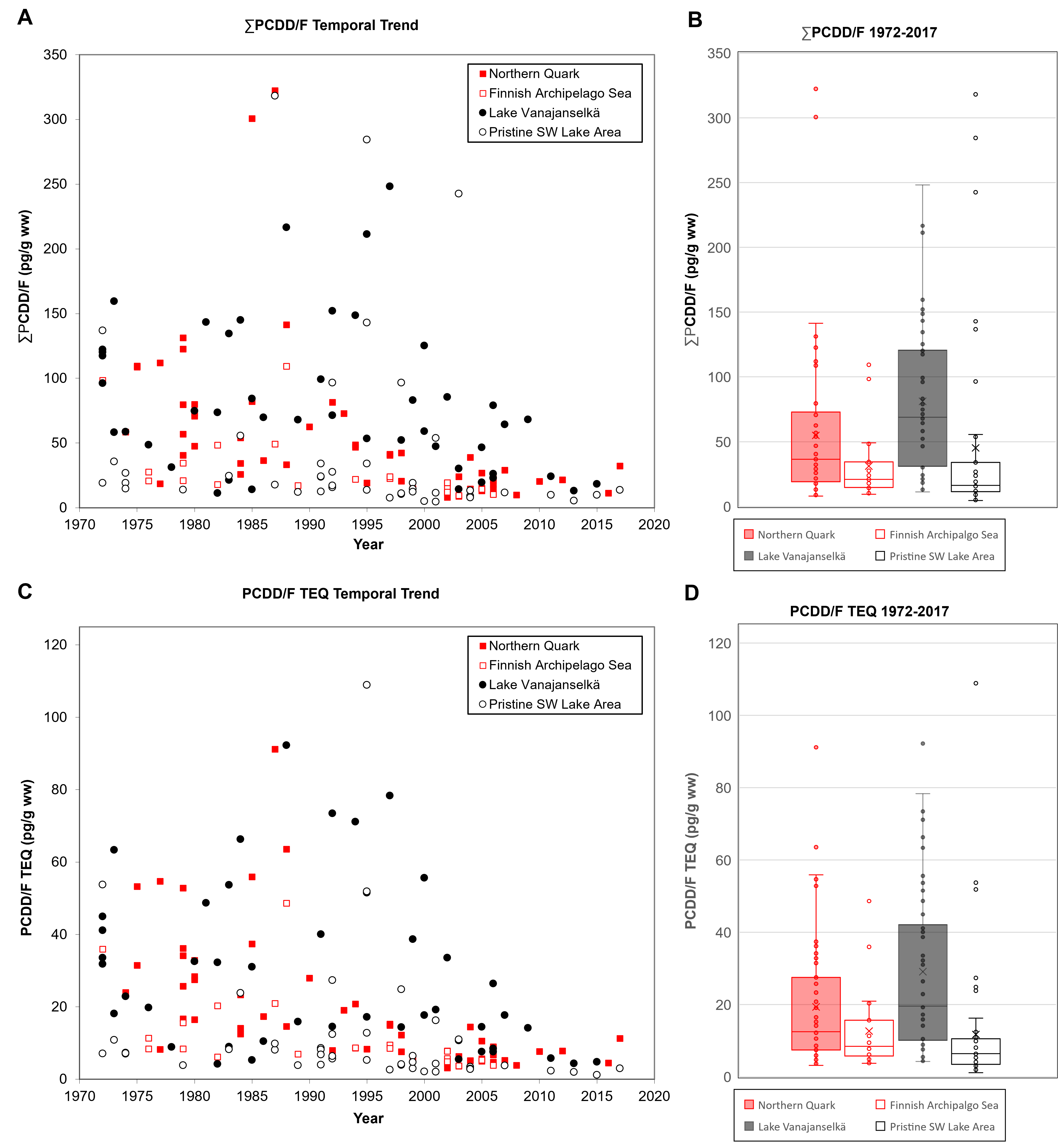

Supplement: S2 Fig — In left panel ∑PCDD/F (A) and PCDD/F TEQ (C) show decreasing trends since 1980’s-1990’s until 2010. In right panel ∑PCDD/F (B) and PCDD/F TEQ (D) means (x), medians (horizontal line), interquartile ranges, min and max datapoints excluding outliers (exceeding 1.5 times the interquartile range; bars) and individual values of all samples (1972–2017) in different study areas are shown. ∑PCDD/F and PCDD/F TEQ followed the same pattern, and the highest levels were in Lake Vanajanselkä and Northern Quark and in a few outliers in Pristine SW Lake Area. (TIF) [file pone.0308227.s004.tif]

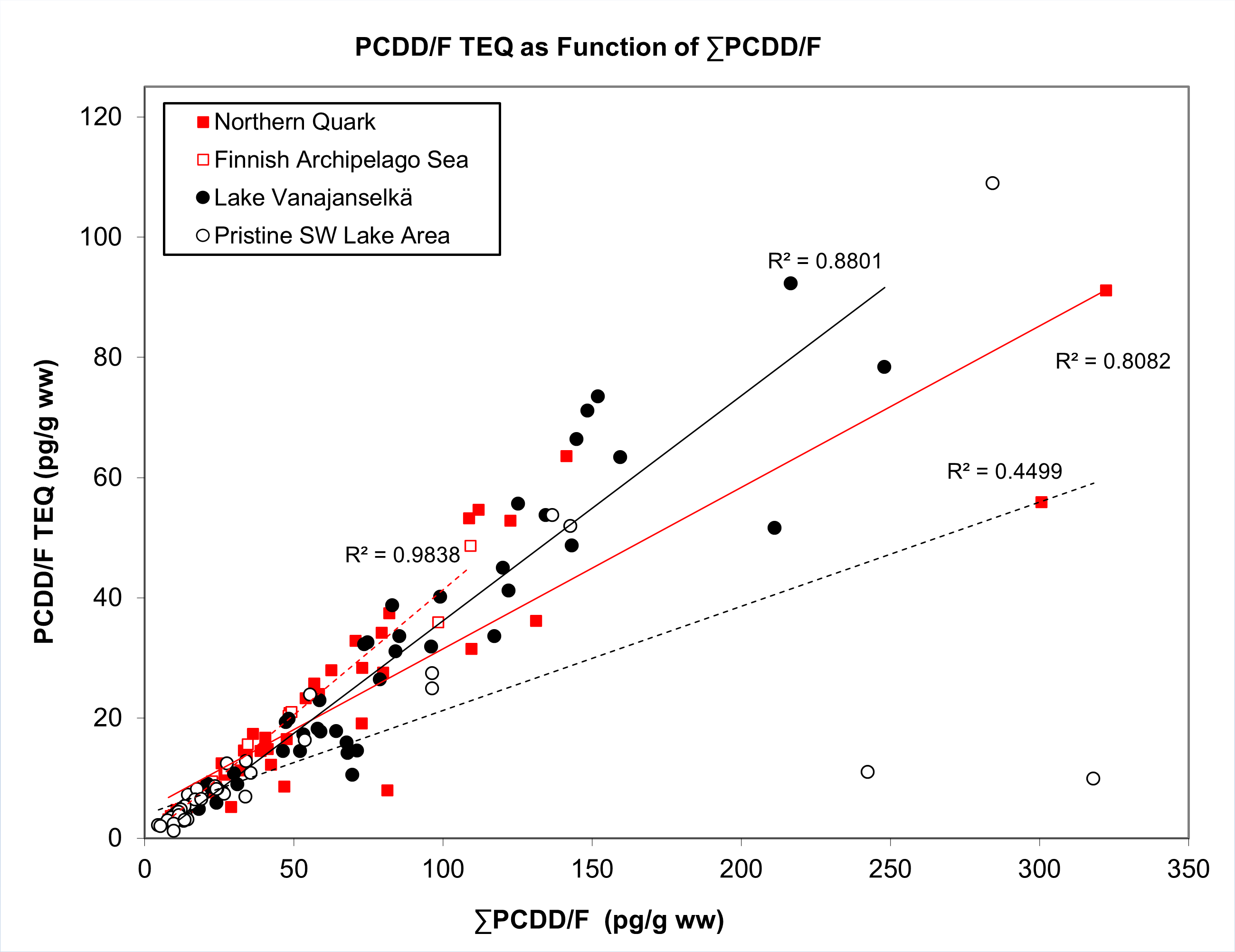

Supplement: S3 Fig — The correlation was very good in Finnish Archipelago Sea followed by Lake Vanajanselkä and Northern Quark, but poor in Pristine SW Lake Area. (TIF) [file pone.0308227.s005.tif]

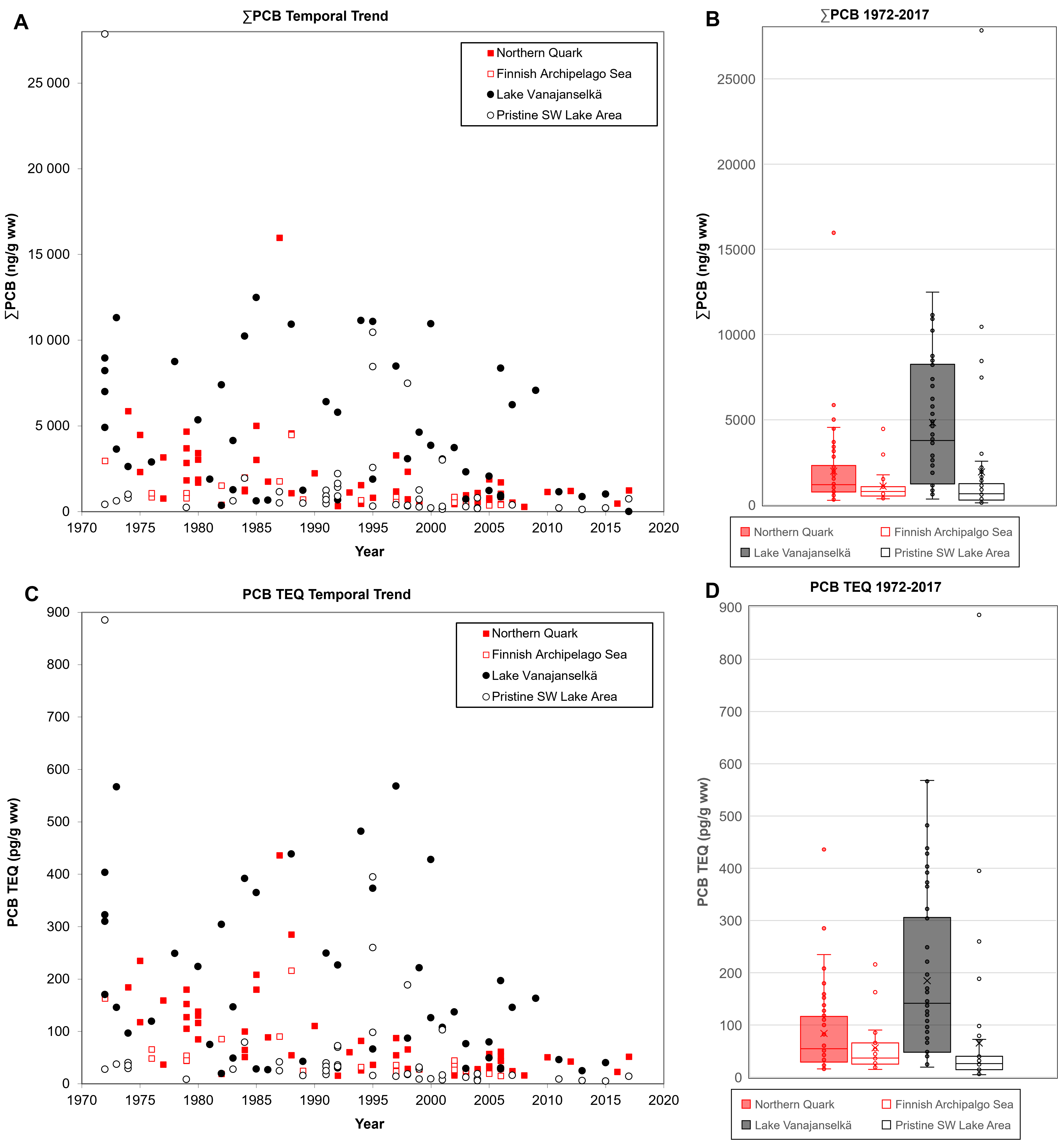

Supplement: S4 Fig — In left panel ∑PCB (A) and PCB TEQ (C) show decreasing temporal trends that depend on study area until 2010. In right panel ∑PCB (B) and PCB TEQ (D) means (x), medians (horizontal line), interquartile ranges, min and max datapoints excluding outliers (exceeding 1.5 times the interquartile range; bars) and individual values of all samples (1972–2017) in different study areas are shown. Similarly with PCDD/Fs, ∑PCB and PCB TEQ followed the same pattern, and the highest levels were in Lake Vanajanselkä and Northern Quark and in a few outliers in Pristine SW Lake Area. (TIF) [file pone.0308227.s006.tif]

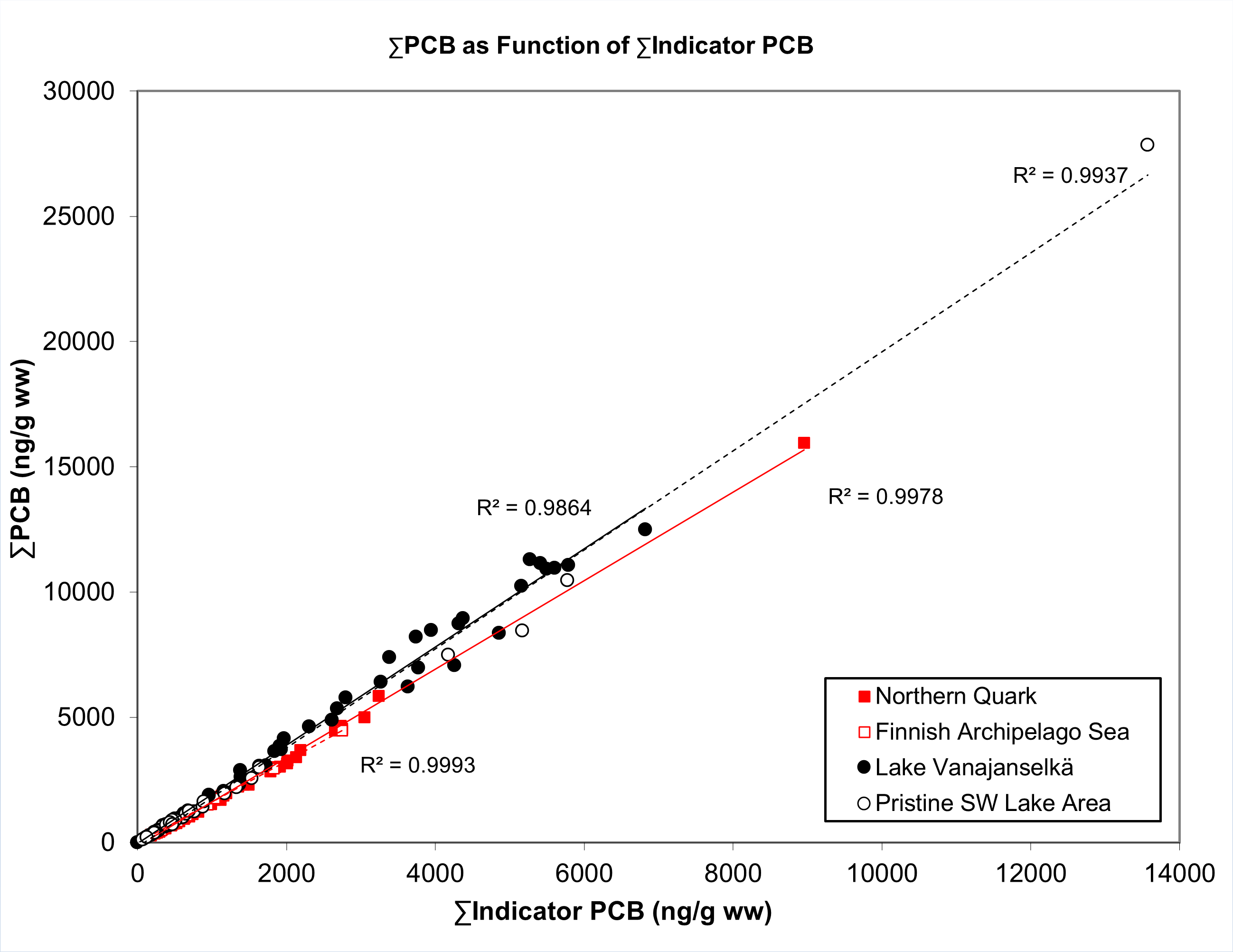

Supplement: S5 Fig — The correlation was very strong in all study areas, which confirms the validity of the indicator PCBs. Indicator PCBs (PCBs 28, 52, 101, 138, 153, 180) have been selected to represent the most abundant congeners across the compositional range of most common technical mixtures and the environment. (TIF) [file pone.0308227.s007.tif]

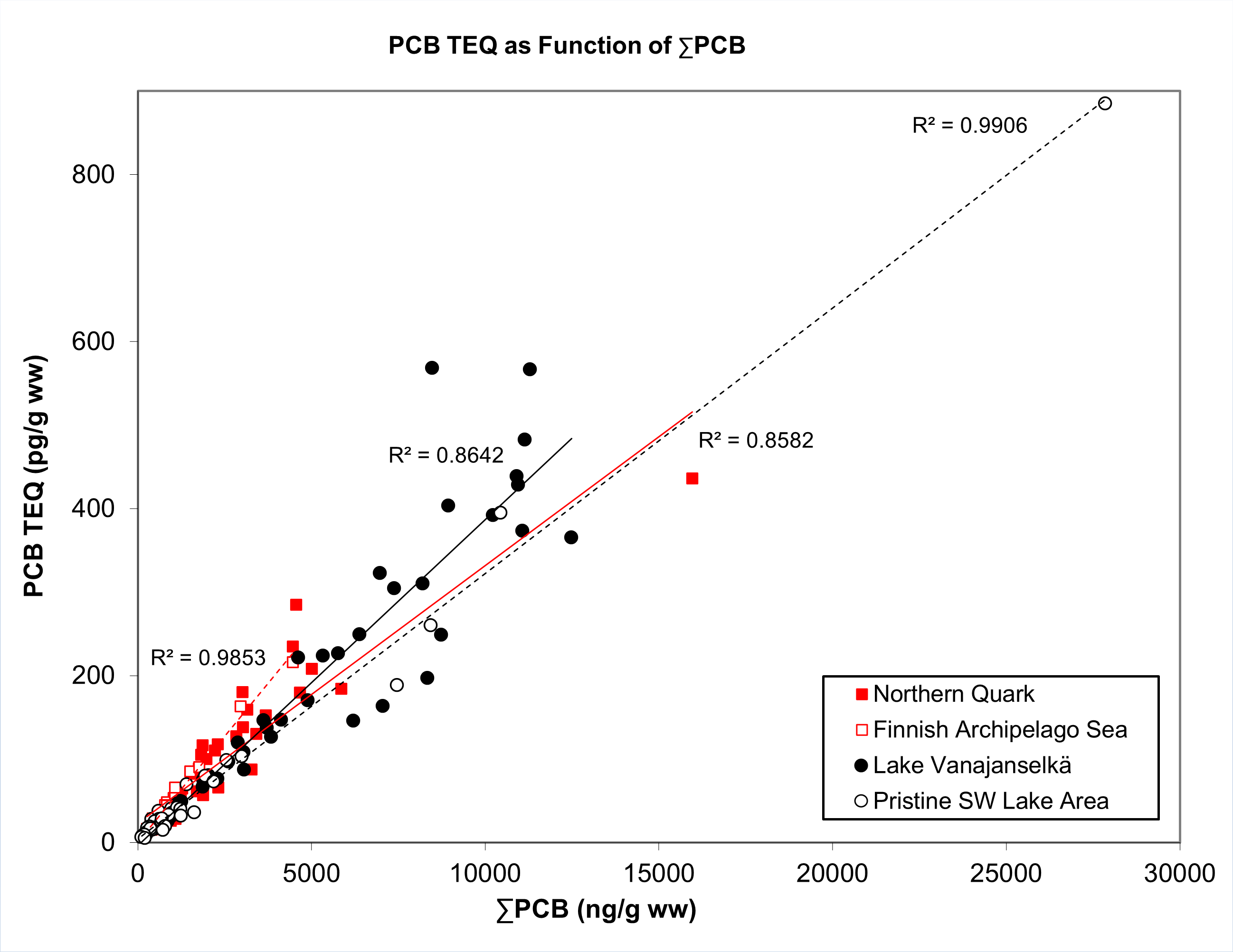

Supplement: S6 Fig — The correlation was very good in Pristine SW Lake Area and in Finnish Archipelago Sea and slightly weaker in Lake Vanajanselkä and Northern Quark. (TIF) [file pone.0308227.s008.tif]

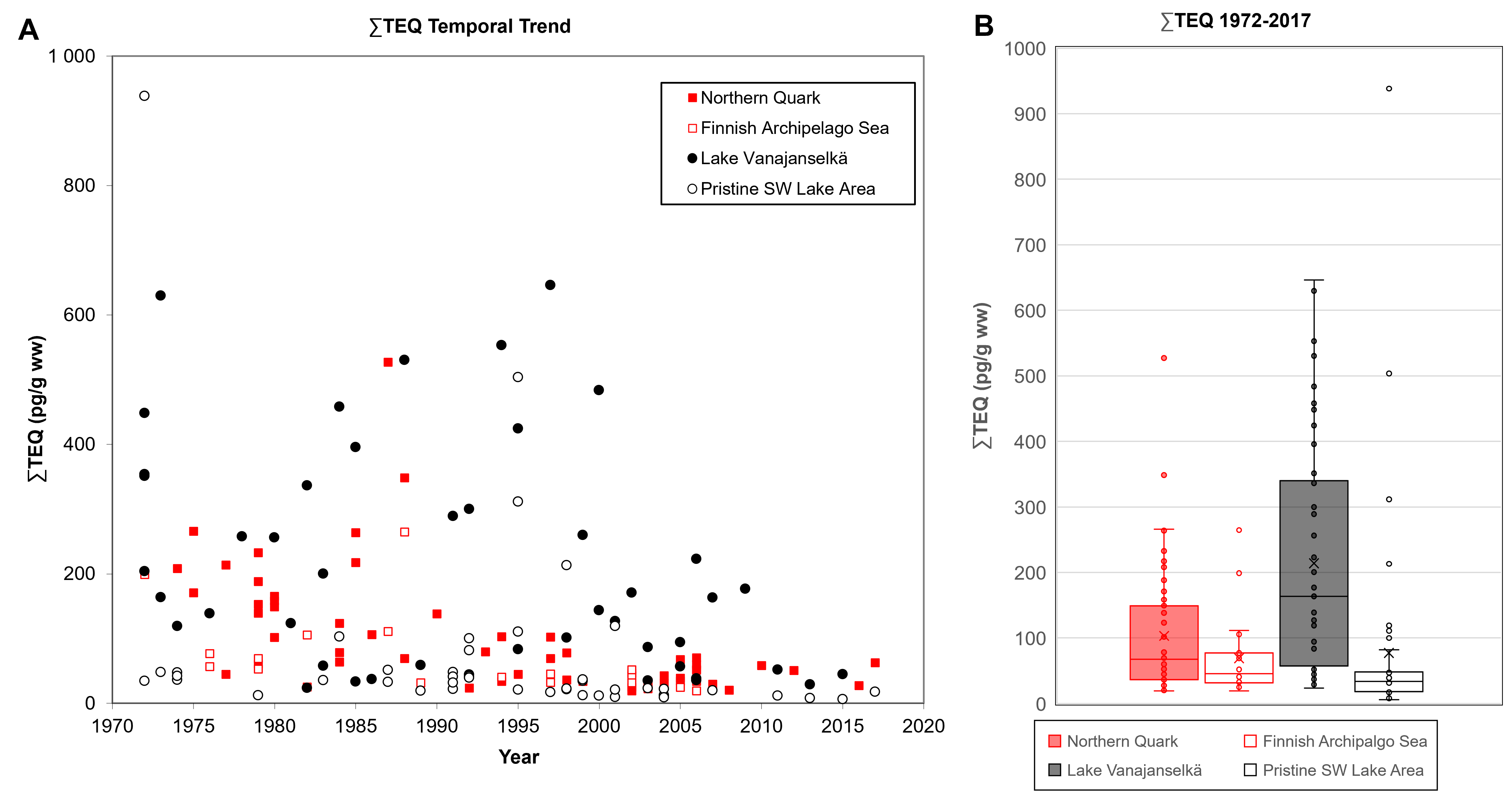

Supplement: S7 Fig — Individual data show decreasing temporal trends that depend on study area until 2010 (A). PCDD/F TEQ + PCB TEQ means (x), medians (horizontal line), interquartile ranges, min and max data points excluding outliers (exceeding 1.5 times the interquartile range; bars) and individual values of all samples (1972–2017) in different study areas (B). The highest levels were in Lake Vanajanselkä and Northern Quark and in a few outliers in Pristine SW Lake Area. (TIF) [file pone.0308227.s009.tif]

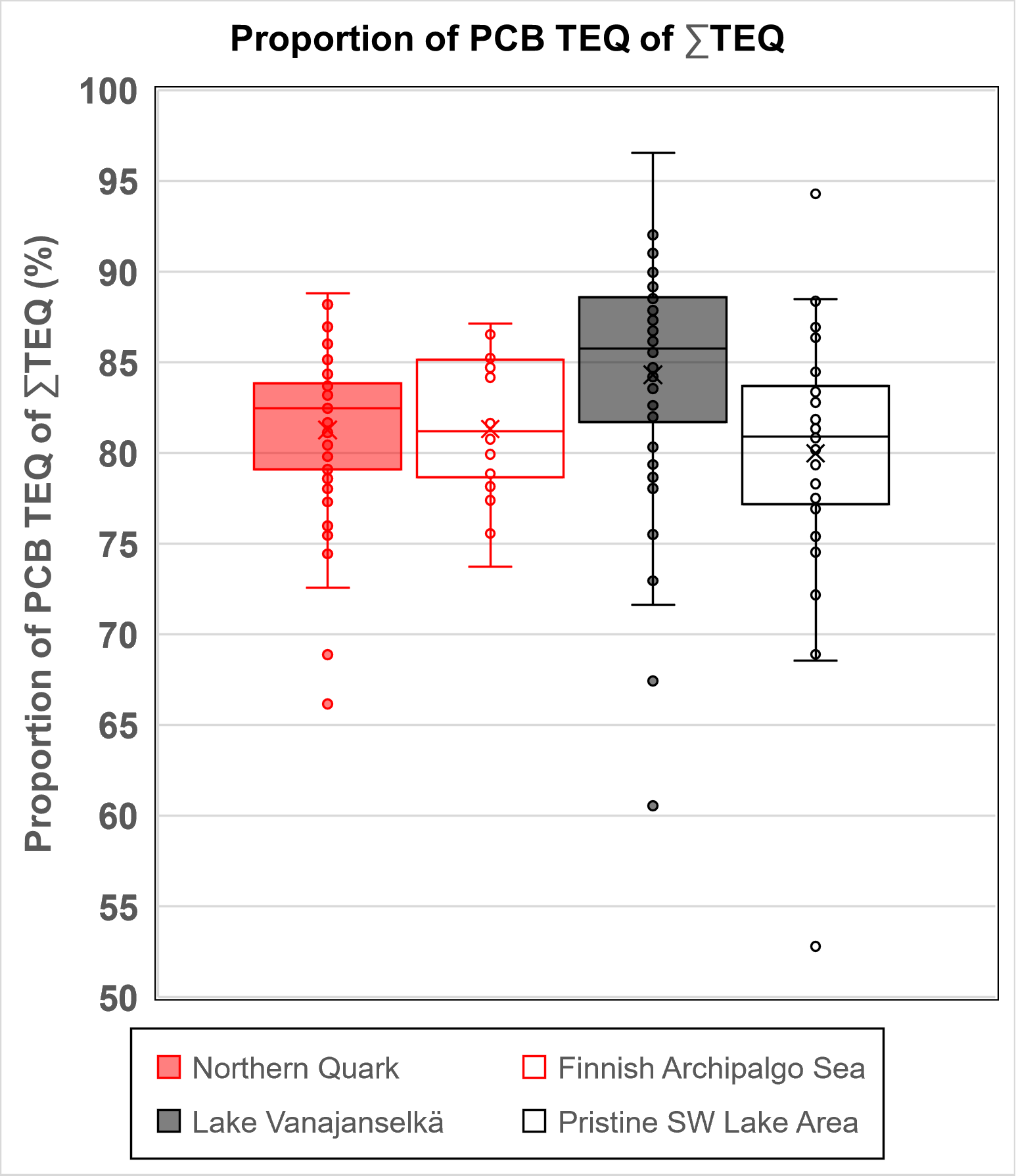

Supplement: S8 Fig — Means (x), medians (horizontal line), interquartile ranges, min and max datapoints excluding outliers (exceeding 1.5 times the interquartile range) and individual values of all samples (1972–2017) in different study areas. PCB TEQs contribute over 80% of ∑TEQ in all study areas and the highest proportion of PCB TEQs is in Lake Vanajanselkä. (TIF) [file pone.0308227.s010.tif]

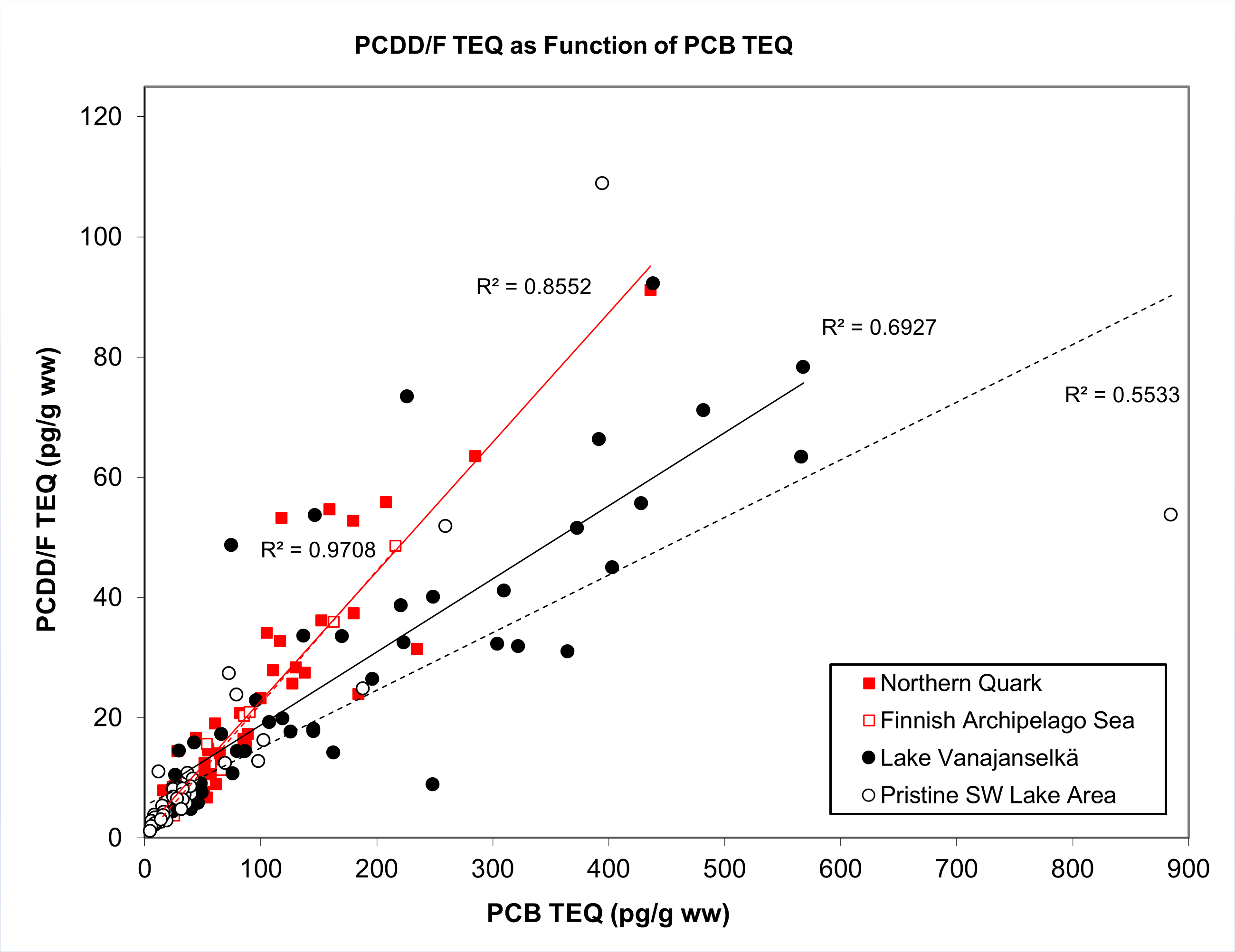

Supplement: S9 Fig — The correlation was very good in Finnish Archipelago Sea and quite good in Northern Quark, but weaker in Lake Vanajanselkä and in Pristine SW Lake Area. (TIF) [file pone.0308227.s011.tif]

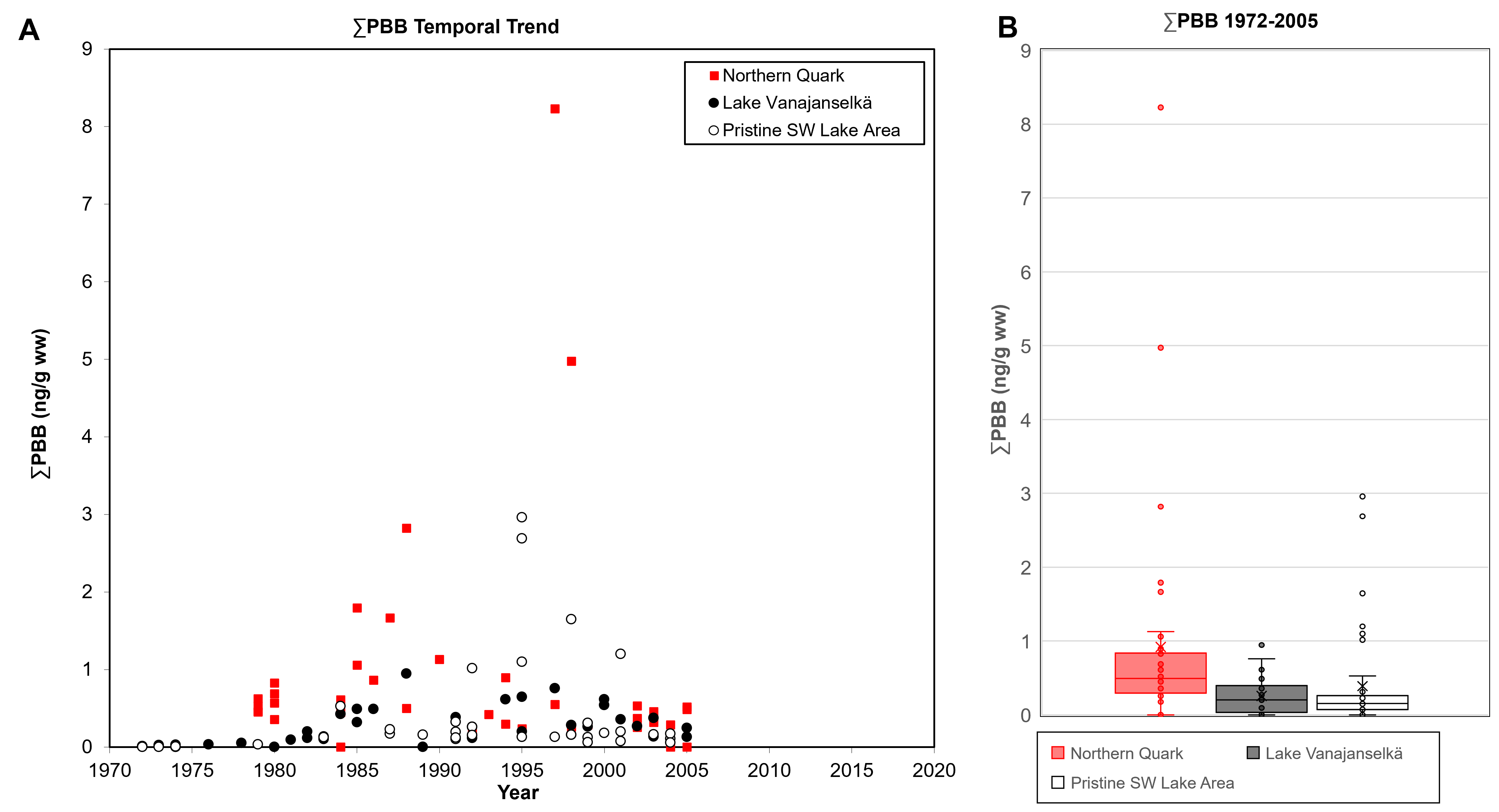

Supplement: S10 Fig — Individual data (A) indicate an increase in the early 1980’s, a peak in mid-late 1990’s followed by a decrease depending on study area. ∑PBB means (x), medians (horizontal line), interquartile ranges, min and max data points excluding outliers (exceeding 1.5 times the interquartile range; bars) and individual values of all samples (1972–2005) in different study areas (B). The highest levels were in Northern Quark and in outliers in Pristine SW Lake Area. (TIF) [file pone.0308227.s012.tif]

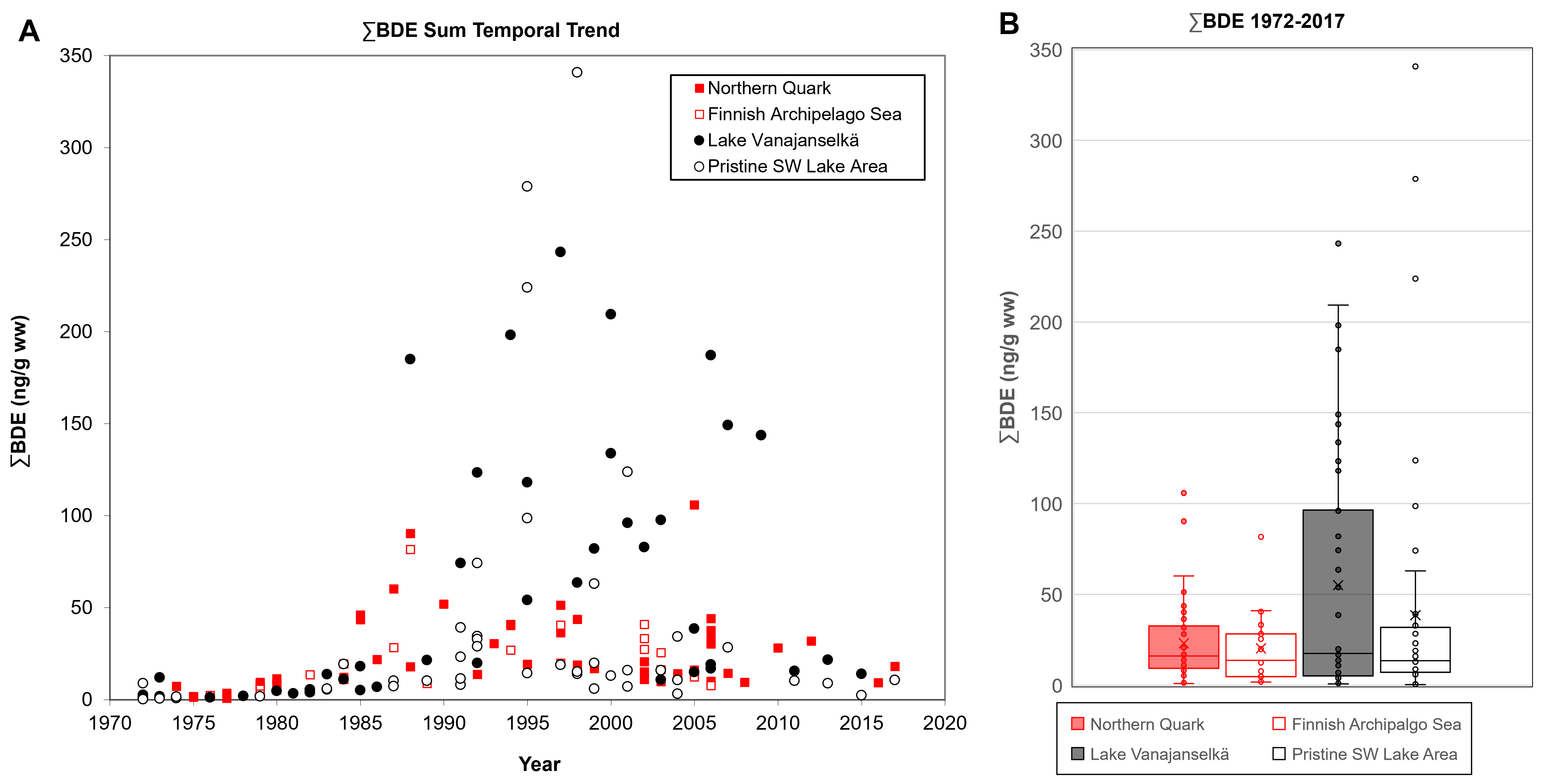

Supplement: S11 Fig — Individual data (A) show an increase in mid-1980’s, peak in late 1990’s and decline rapidly before 2010. ∑BDE means (x), medians (horizontal line), interquartile ranges, min and max data points excluding outliers (exceeding 1.5 times the interquartile range; bars) and individual values of all samples (1972–2017) in different study areas (B). The highest levels were in Lake Vanajanselkä and in a few outliers in Pristine SW Lake Area. (TIF) [file pone.0308227.s013.tif]

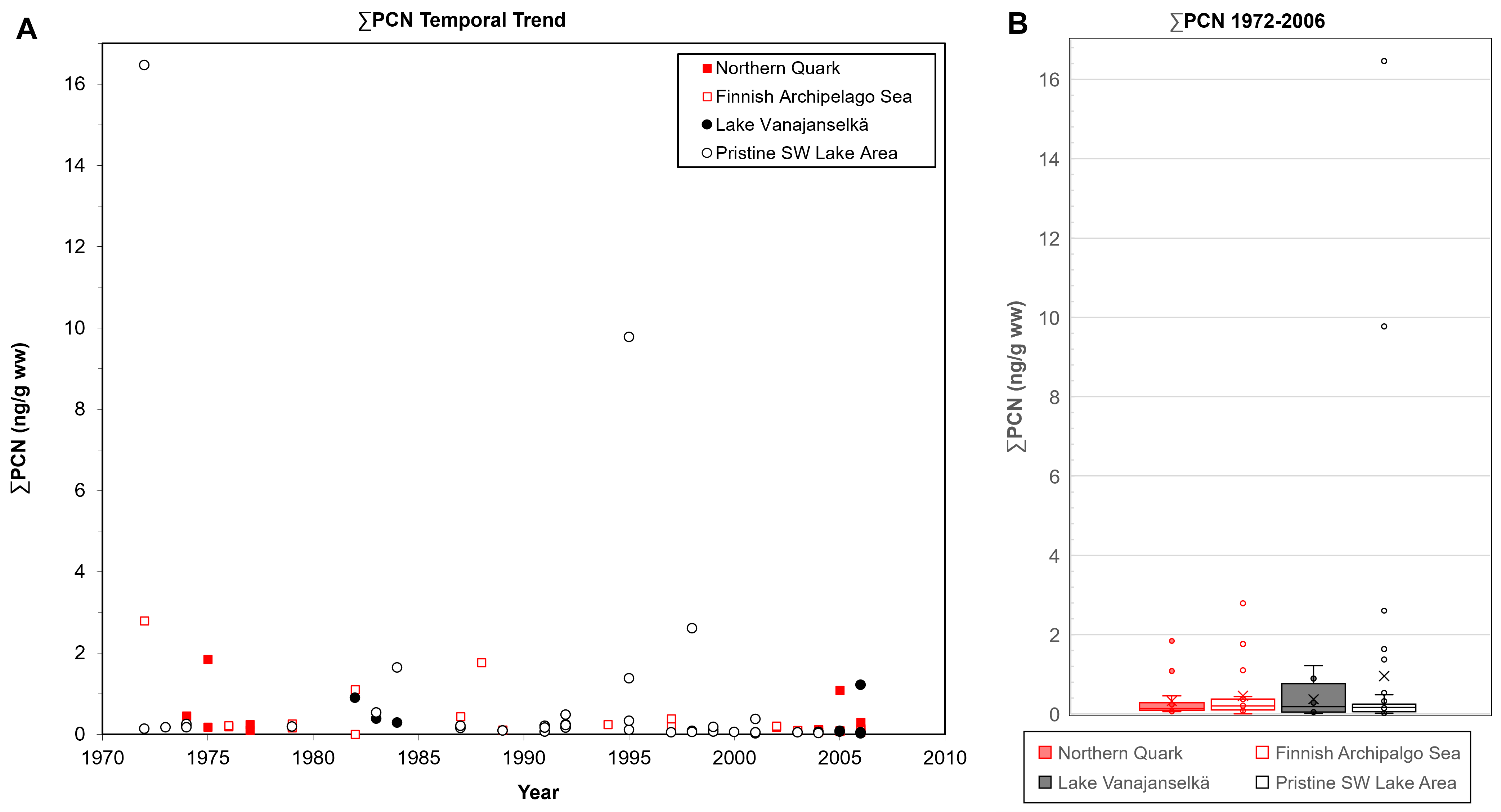

Supplement: S12 Fig — Individual data (A) indicate no major temporal trends. ∑PCN means (x), medians (horizontal line), interquartile ranges, min and max data points excluding outliers (exceeding 1.5 times the interquartile range; bars) and individual values of all samples in different study areas during 1972–2006 (B). Overall, the levels were quite similar in all study areas. (TIF) [file pone.0308227.s014.tif]

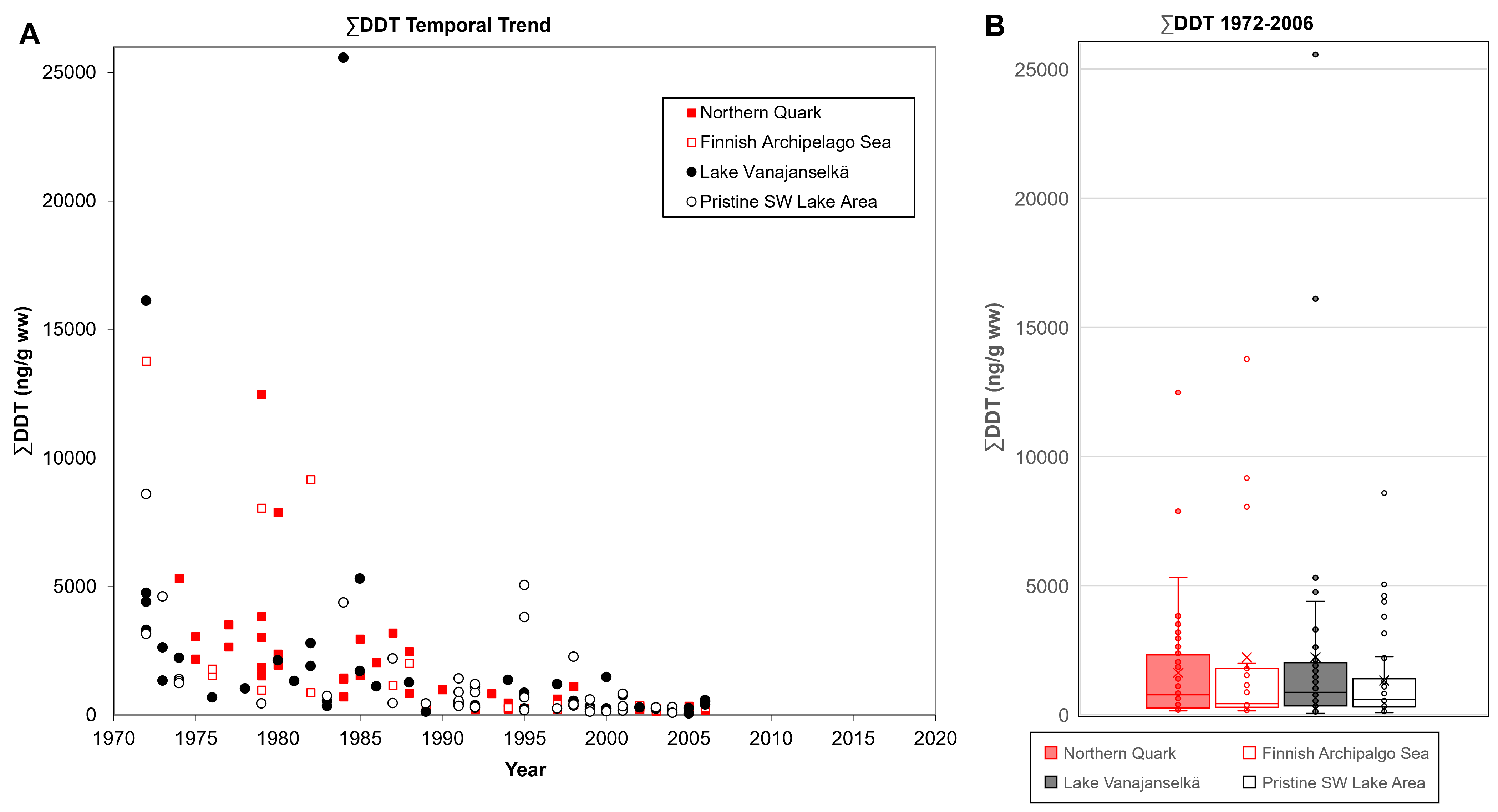

Supplement: S13 Fig — Individual data in different study areas (A) indicating a steady decline until the late 1980’s followed by a minor decline until early 2000. ∑DDT means (x), medians (horizontal line), interquartile ranges, min and max data points excluding outliers (exceeding 1.5 times the interquartile range; bars) and individual values of all samples (1972–2006) in different study areas (B). There were high individual values in all study areas (highest in Lake Vanajanselkä) until the late 1990’s after which the levels were quite similar. (TIF) [file pone.0308227.s015.tif]

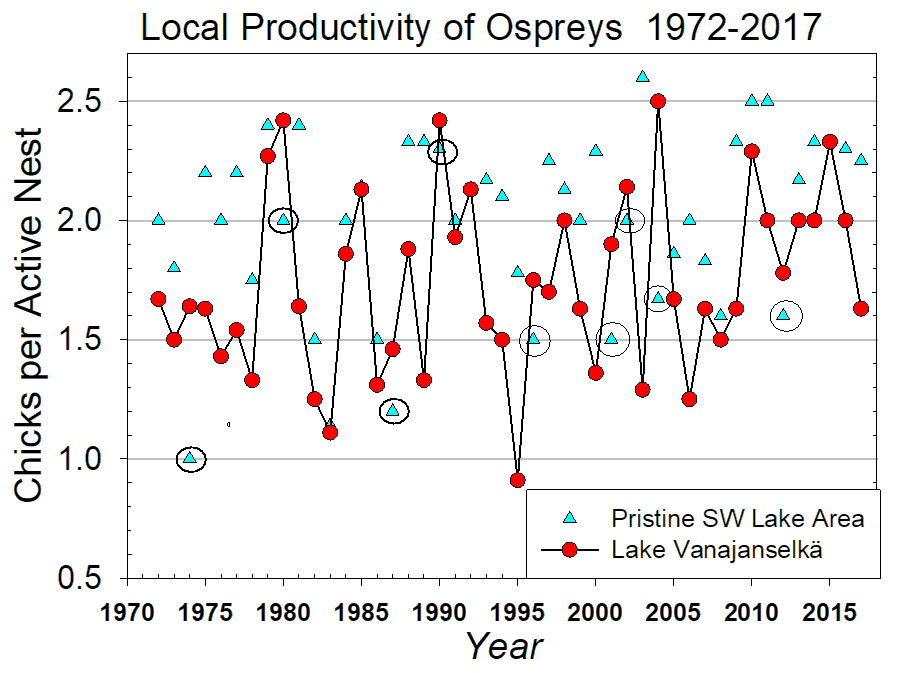

Supplement: S14 Fig — In 46 years, productivity has been lower in the Pristine SW Lake Area than in the Lake Vanajanselkä only in nine years (marked with circles). The difference is statistically significant (p<0.001, Paired T-test). Data from the database of the Finnish Museum of Natural History. (TIF) [file pone.0308227.s016.TIF]

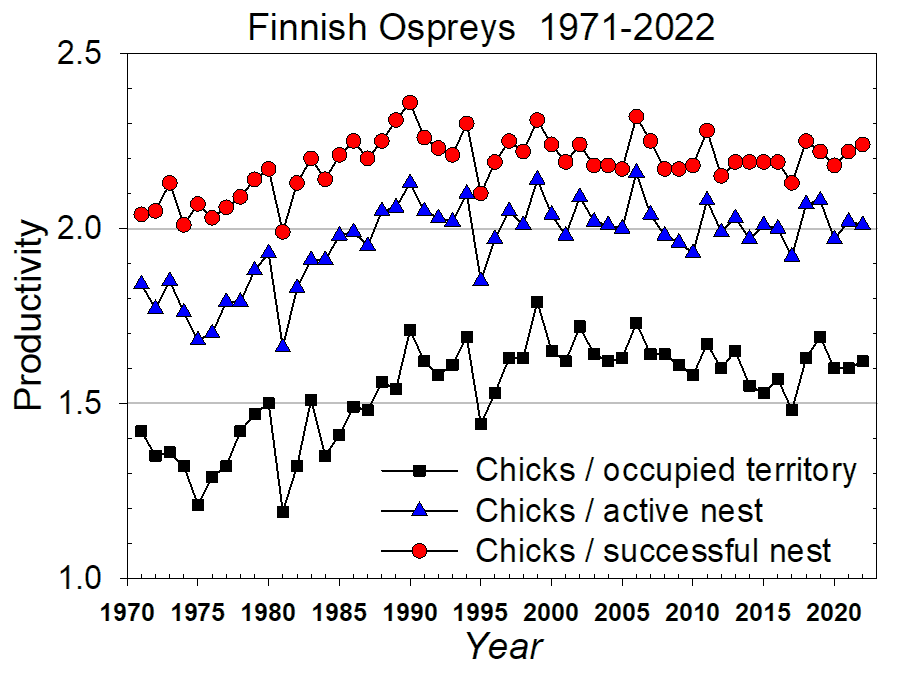

Supplement: S15 Fig — Black square = chicks / occupied territory, blue triangle = chicks / active nest, red dot = chicks / successful nest. Data from the database of the Finnish Museum of Natural History [31]. (TIF) [file pone.0308227.s017.TIF]

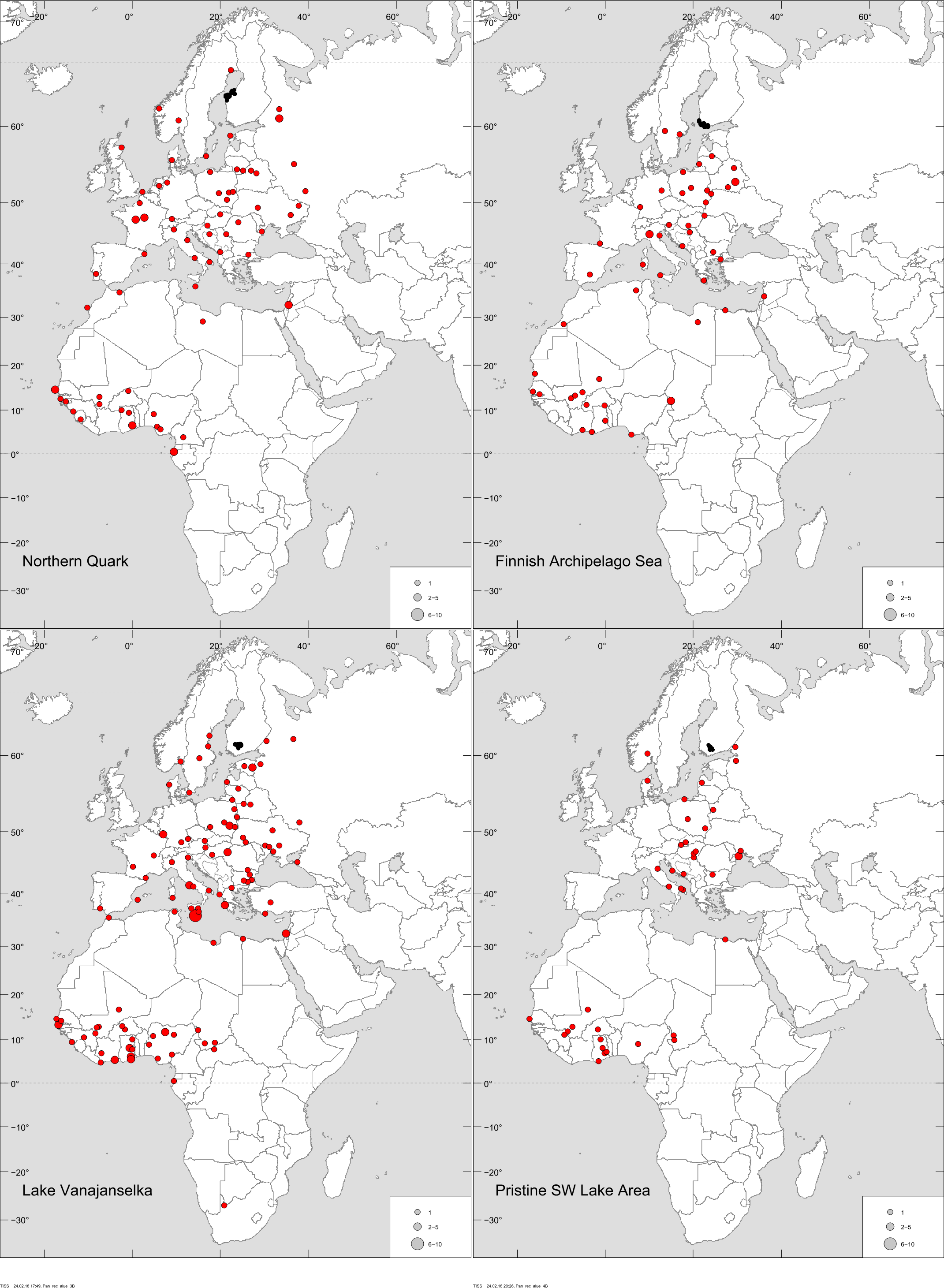

Supplement: S16 Fig — Black dots are ringing sites and red dots encounter sites. Locations of ring encounters of ospreys ringed in areas of Northern Quark and Finnish Archipelago Sea did not significantly differ from ospreys ringed in areas of Lake Vanajanselkä and Pristine SW Lake Area. Data from the Finnish Museum of Natural History. The map was drawn by the TISS application developed by AB based on coordinates from the CIA public domain database. (TIF) [file pone.0308227.s018.tif]
