# Supplementary material for: Temporal trends (1972–2017) and spatial differences of persistent halogenated aromatic hydrocarbons in osprey eggs in Finland
Source: PLoS One. 2024 Sep 3;19(9):e0308227. doi: 10.1371/journal.pone.0308227 (PMC11371234; doi:10.1371/journal.pone.0308227)
Supplement: S1 Table — Data from M. Finnlund and P. Saurola. Difference between bream and pike and between cyprinid and predatory fish is statistically significant (p<0.001, Fischer’s Exact Test). (DOCX) [file pone.0308227.s019.docx]

**S1 Table. A dataset on prey fish species of ospreys in Northern Quark and Vanajanselkä area.** Data from M. Finnlund and P. Saurola. Difference between bream and pike and between cyprinid and predatory fish is statistically significant (p<0.001, Fischer’s Exact Test).

|  |  |  |  |  |  |
| --- | --- | --- | --- | --- | --- |
|  |  | **Northern Quark** | | **Vanajanselkä Area** | |
|  |  | 1974-2006 | | 1972-2023 | |
| **Species** | | **Count** | **%** | **Count** | **%** |
| Bream*^a^* | *Abramis brama* | 5 | 2.0 | 141 | 50.4*** |
| Crucian carp*^a^* | *Carassius carassius* | 11 | 4.3 | 29 | 10.4 |
| Roach*^a^* | *Rutilus rutilus* | 34 | 13.3 | 27 | 9.6 |
| Pike*^b^* | *Esox lucius* | 104 | 40.8 | 18 | 6.4*** |
| Rainbow trout*^b^* | *Oncorhynchus mykiss* | 1 | 0.4 | 17 | 6.1 |
| Perch*^b^* | *Perca fluviatilis* | 89 | 34.9 | 13 | 4.6 |
| Ide*^a^* | *Leuciscus idus* | 2 | 0.8 | 11 | 3.9 |
| White bream*^a^* | *Blicca bjoerkna* |  |  | 6 | 2.1 |
| Pikeperch*^b^* | *Sander lucioperca* |  |  | 6 | 2.1 |
| Blue bream*^a^* | *Ballerus ballerus* |  |  | 5 | 1.8 |
| Tench | *Tinca tinca* |  |  | 4 | 1.4 |
| Rudd*^a^* | *Scardinius erythrophtalmus* |  |  | 3 | 1.1 |
| Whitefish | *Coregonus lavaretus* | 8 | 3.1 |  |  |
| Sculpins | *Cottidae* | 1 | 0.4 |  |  |
|  | Sum | 255 | 100 | 280 | 100 |
| *^a^*Cyprinid fish |  | 95 | 20.4 | 182 | 79.3*** |
| *^b^*Predatory fish |  | 194 | 76.1 | 54 | 19.3*** |
|  |  |  |  |  |  |
